# Single-plant-omics reveals the cascade of transcriptional changes during the vegetative-to-reproductive transition

**DOI:** 10.1093/plcell/koae226

**Published:** 2024-08-09

**Authors:** Ethan J Redmond, James Ronald, Seth J Davis, Daphne Ezer

**Affiliations:** Department of Biology, University of York, Wentworth Way, Heslington, York YO10 5DD, UK; Department of Biology, University of York, Wentworth Way, Heslington, York YO10 5DD, UK; School of Molecular Biosciences, College of Medical, Veterinary and Life Sciences, University of Glasgow, Glasgow G12 8QQ, UK; Department of Biology, University of York, Wentworth Way, Heslington, York YO10 5DD, UK; Department of Biology, University of York, Wentworth Way, Heslington, York YO10 5DD, UK

## Abstract

Plants undergo rapid developmental transitions, which occur contemporaneously with gradual changes in physiology. Moreover, individual plants within a population undergo developmental transitions asynchronously. Single-plant-omics has the potential to distinguish between transcriptional events that are associated with these binary and continuous processes. Furthermore, we can use single-plant-omics to order individual plants by their intrinsic biological age, providing a high-resolution transcriptional time series. We performed RNA-seq on leaves from a large population of wild-type Arabidopsis (*Arabidopsis thaliana*) during the vegetative-to-reproductive transition. Though most transcripts were differentially expressed between bolted and unbolted plants, some regulators were more closely associated with leaf size and biomass. Using a pseudotime inference algorithm, we determined that some senescence-associated processes, such as the reduction in ribosome biogenesis, were evident in the transcriptome before a bolt was visible. Even in this near-isogenic population, some variants are associated with developmental traits. These results support the use of single-plant-omics to uncover rapid transcriptional dynamics by exploiting developmental asynchrony.

## Teaser

Transcriptome sequencing of leaves from many wild-type plants during bolting revealed transcriptional changes corresponding to binary and gradual developmental traits at high temporal resolution.

**IN A NUTSHELL**

A detailed sequence of transcriptional events that occur immediately before and after bolting is revealed by sequencing a large population of asynchronously developing wildtype Arabidopsis.

## Introduction

Plants experience many gradual developmental changes, such as leaf and biomass accumulation. However, at certain critical points, they undergo rapid “phase changes” that act as switches. These switches include the juvenile-to-vegetative transition and the vegetative-to-reproductive transition (Rankenberg et al. 2021). Transcriptional programs coordinate both these gradual and binary developmental processes. Understanding rapid transcriptional changes, requires a high temporal resolution of sample collection (Ezer and Keir 2019), a strategy that has effectively been deployed to detect rapid transcriptional responses to the onset of light (Balcerowicz et al. 2021) and heat (Cortijo et al. 2017). However, this experimental approach does not easily translate to the study of late developmental transitions, because individual plants do not undergo developmental transitions in a synchronized way, and the degree of asynchrony is greater than the required temporal resolution of sampling. This developmental asynchrony—also known as “developmental instability” (Klingenberg 2019)—is caused by the inherently stochastic nature of biochemical processes, such as transcriptional bursting (Leyes Porello et al. 2023). Developmental asynchrony is also affected by genetic factors and heterogeneity in microenvironments (Pertoldi et al. 2006; Klingenberg 2019).

Single-plant-omics offers an innovative way to investigate the transcriptional dynamics at developmental transition points. Single-plant-omics has previously been used to characterize diurnal gene-expression noise (Cortijo et al. 2019), maize (*Zea mays*) transcriptional heterogeneity within a field-grown population (Cruz et al. 2020), and meristem heterogeneity in tomatoes (Meir et al. 2021). By exploiting the developmental asynchrony across individual plants, we can reconstruct the order of transcriptional cascades that occur during a rapid developmental transition.

We focus on the vegetative-to-reproductive transition, an irreversible phase change in angiosperms that includes the formation of a bolt and eventually leads to flowering. Climate change has already been shown to reduce the synchrony of flowering time, leading to ecological and agricultural repercussions (Zohner et al. 2018). To support the initiation of reproductive growth, multiple processes are tightly coordinated within leaves during this transition. Of particular importance is the onset of leaf senescence: a transcriptionally regulated and tightly controlled process that leads to cell death in leaves (Kim et al. 2018) and nutrient reallocation to reproductive structures (Havé et al. 2017). Bolting-associated leaf senescence is an important contributor to the nutrient quality of grains (Gregersen et al. 2008; Havé et al. 2017).

In this paper, we utilize single-plant-omics of a population of wild-type plants to reconstruct the transcriptional cascades that control development during the onset of bolting in Arabidopsis leaves. We found that the majority of genes were differentially expressed during bolting, reflecting a binary shift in transcriptional states. Next, we identified a subset of genes that are more closely associated with the gradual transcriptional changes associated with growth. Finally, we order the plants on the basis of their biological age, which enables us to uncover the sequence of events that occur during the bolting process, which begins with a shutdown of ribosome production and ends with the shutdown of photosynthesis. The gene network that we infer can serve as a reference for understanding the transcriptional regulation pathways that underlie this critical developmental transition. Additionally, we identified genetic variants that are associated with biological age. We show that single-plant-omics aids understanding of complex developmental transitions.

## Results

### Widespread transcriptional changes occur after bolting in *Arabidopsis thaliana*

Bolting functions as an indicator of the vegetative-to-reproductive developmental transition and occurs simultaneously with the onset of leaf senescence and the cessation of leaf development (Möller-Steinbach et al. 2010; Hinckley and Brusslan 2020). To investigate the gene-expression changes that arise during bolting, we measured gene expression from leaves of individuals within a population of wild-type Arabidopsis (*A. thaliana*) plants, grown under uniform, controlled conditions (*n* = 68 after filtering; see Materials and methods, Supplementary Fig. S1 and Data Set 1) and under inductive conditions (16 h light/8 h dark). These were sampled when 23 of the plants had bolted. To explore the connection between asynchronous gene expression and the major developmental changes occurring around the vegetative-to-reproductive transition, we recorded key developmental physiological traits from each plant.

We performed hierarchical clustering of plants, based on the Pearson correlation of gene expression between samples (Supplementary Fig. S2). This analysis showed that the samples can be separated into 2 clusters. One cluster contains the majority of the nonbolted plants (35 out of 45) and the other contains the majority of the bolted plants (18 out of 23; Supplementary Fig. S2 and Data Set 2). We thus sought to characterize the transcriptomic changes occurring simultaneously with bolting. We found differentially expressed genes (DEGs) between nonbolted and bolted plants, taking into account the large number of samples (see Materials and methods, Supplementary Data Set 3 and Fig. S3). Overall, 3,734 genes were upregulated, and 6,967 genes were downregulated after bolting, accounting for 55% of the entire transcriptome after initial filtering (Supplementary Fig. S1). We performed gene ontology (GO) term enrichment analysis on the DEGs (Raudvere et al. 2019). Significantly, enriched terms cover a range of senescence-associated processes, consistent with previous observations that transcriptional changes during bolting are associated with senescence (Supplementary Data Set 4; Hinckley and Brusslan 2020).

**Figure 1.**
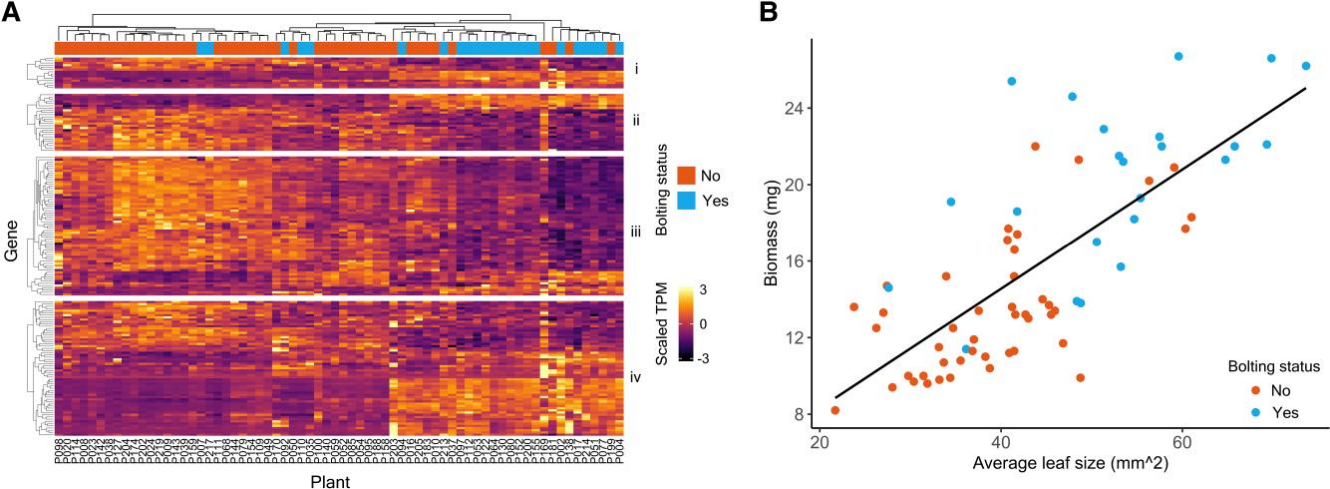
Gene-expression values and physiology. **A)** Gene-expression values across all samples for genes annotated with selected GO terms. Only genes that were identified as differentially expressed are shown. Hierarchical clustering across each sample shows that plants with the same bolting status often cluster together. Additionally, hierarchical clustering within each group of genes indicates the nature of the large changes between nonbolted and bolted plants. (i) Programmed cell death (GO:0012501). Adjusted *P*-values: up = 2.68e−9, down = 1. (ii): Regulation of cell cycle (GO:0051726). Adjusted *P* values: up = 1, down = 8.82e−15. (iii) Photosynthesis (GO:0015979). Adjusted *P*-values: up = 1, down = 3.84e−8. (iv) Response to jasmonic acid (GO:0009753). Adjusted *P*-values: up = 1.97e−8, down = 1. Note that we scaled TPM values (to have a mean of 0 and Sd of 1 across all samples) then clipped any value >3 or <−3. Adjusted *P*-values were calculated by g:Profiler and relate to test of GO term overrepresentation in either up- or downregulated genes (Raudvere et al. 2019). For all hierarchical clustering, we used the “complete” linkage method. **B)** Biomass and leaf size are plotted per individual plant. The black line represents the line of best fit (linear regression, *R*^2^ = 0.55, *P* = 1.59e−12). *n* = 68 total sample size.

First, programmed cell death (GO:0012501) was overrepresented among genes that were upregulated in bolted plants (Fig. 1A (i)). Conversely, the regulation of cell cycle (GO:0051726) is overrepresented within downregulated genes (Fig. 1A (ii)). These results are consistent with changes in cell cycles within maturing leaves. A shift occurs from cell division to cell elongation (marked by endoreduplication) in young to mature leaves, and programmed cell death eventually occurs in senescent tissue (Del Prete et al. 2019; Woo et al. 2019). Second, bolting correlates with a reduction of gene expression for genes associated with each step of transcription and translation, including RNA polymerase complex formation (GO:0030880), RNA processing (GO:0006396), and structural constituents of the ribosome (GO:0003735). In contrast, genes related to posttranscriptional modification, such as ubiquitination (GO:0016567) and protein phosphorylation (GO:0006468), were more highly expressed in bolted plants. Third, photosynthesis-related (GO:0015979) gene expression was reduced in bolted plants (Fig. 1A (iii)), consistent with chlorophyll catabolism during senescence (Woo et al. 2019). Finally, we observed an overrepresentation of genes associated with jasmonic acid (GO:0009753), abscisic acid (GO:0009737), and salicylic acid (GO:0009751). These hormone signaling pathways were previously associated with leaf senescence (Woo et al. 2019). These results indicate that the onset of bolting coincides with a large-scale transcriptomic shift toward senescence.

### Physiological traits have distinct correlations with biological processes

Bolting is a rapid developmental transition that occurs over a 2- or 3-d period, but other plant traits accumulate gradually over a plant's lifetime, including leaf area and biomass. We observed that biomass and leaf size were correlated across the population (linear regression, *R*^2^ = 0.55, *P* = 1.59e−12). Additionally, bolted plants had significantly higher biomass (Mann–Whitney *U* test, *W* = 111.5, *P* = 1.46e−7) and leaf size (Mann–Whitney *U* test, *W* = 185, *P* = 6.33e−6) than nonbolted plants (Fig. 1B, Supplementary Data Set 2).

**Figure 2.**
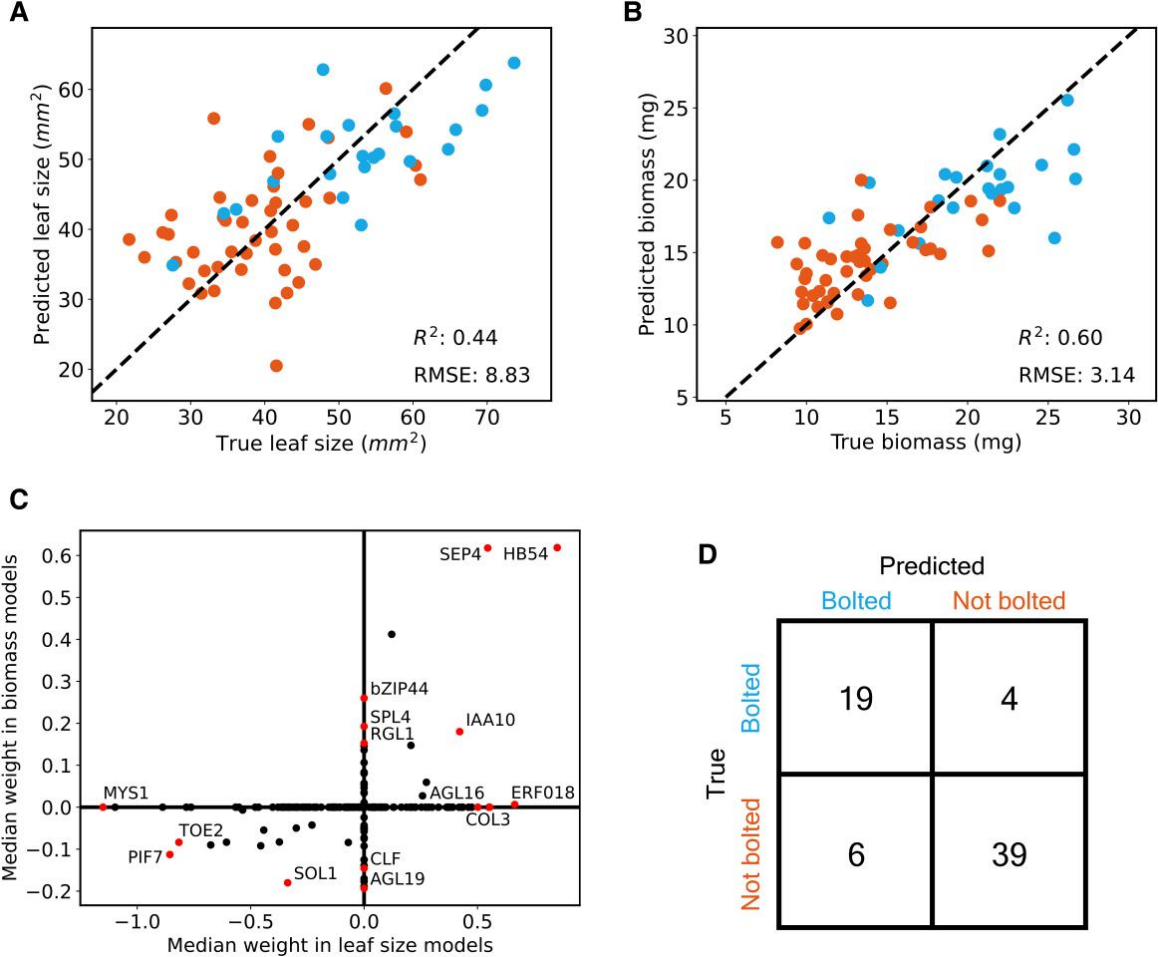
Predicting physiology from gene expression. For **A** and **B**, red represents nonbolted plants (*n* = 45) and blue represents bolted plants (*n* = 23). **A)** Performance of leaf size prediction from regulatory genes. Predicted values were produced from an elastic net model, trained on all data except for the relevant sample. **B)** A repeat of **A** for biomass values. **C)** Comparison of the median weight of predictors from biomass and leaf size models. **D)** Confusion matrix of the logistic regression models trained to predict bolting status from gene expression. Predicted values were produced from a logistic regression model, trained on all data except for the relevant sample. Each model was regularized using the elastic net method. The accuracy across all logistic regression models is 85.3%. (See Physiology trait prediction with elastic nets section for more information on cross-validation, hyperparameter selection, and performance metrics.) RMSE, root mean squared error.

While we previously observed widespread transcriptional changes associated with the bolting transition, we hypothesized that there would be separate transcriptional programs associated with leaf area and biomass. To investigate this, we used regularized linear regression, via elastic net models, to predict physiology from gene expression for all the plants in our population (Zou and Hastie 2005). We used leave-one-out-cross-validation (LOOCV) to validate the generalizability of our models (Hastie et al. 2009; Supplementary Data Set 5). We chose to focus on models trained on regulatory genes only (see Materials and methods, for a more complete definition), which enabled us to interpret the predictors from these models more easily (Fig. 2C). Despite the slightly worse *R*^2^ and relative mean squared error (RMSE) scores for leaf models, the biomass models performed better when restricted to only regulators (see Fig. 2, A and B, Supplementary Fig. S4, A and B, and Data Set 6).

We were able to identify genes that were consistently identified as predictors of both leaf area and biomass in multiple models during the LOOCV process (Fig. 2C, Supplementary Data Set 7). Since gene expression was standardized before input to all models, it was possible to compare the coefficients of different genes within a model. Overall, 180 regulatory genes had a nonzero median coefficient across all models (Supplementary Data Set 7). Of these, 8 were identified as positive predictors of biomass and leaf size and 13 were identified as negative predictors of both traits (Supplementary Data Set 7). The gene with the highest positive coefficient for both traits was *HOMEOBOX PROTEIN 54* (*HB54*), involved in a nitrogen-signaling cascade linked to plant growth (Ariga et al. 2022). An auxin-responsive gene, *INDOLEACETIC ACID–INDUCED PROTEIN 10* (*IAA10*), was identified as a positive regulator of leaf size and biomass (Liscum and Reed 2002).

We identified 3 AGAMOUS-LIKE MADS-box TFs with high positive or negative coefficients for these traits. Orthologs of similar TFs in rapeseed were highlighted in a single-plant-omics study as predictors of yield phenotypes (De Meyer et al. 2023). *SEPALLATA 4* (*SEP4*), also known as *AGAMOUS-LIKE 3* (*AGL3*), had a high positive coefficient for biomass and for leaf models. *AGAMOUS-LIKE 16* (*AGL16*) had a high positive coefficient for leaf size but not biomass. *AGAMOUS-LIKE 19* (*AGL19*), which is a known component in the *FLOWERING LOCUS C (FLC)-*independent vernalization pathway, had a highly negative median coefficient for biomass models but not leaf models (Schönrock et al. 2006). Interestingly, *CURLY LEAF* (*CLF*) also had a highly negative median coefficient for biomass models but not leaf models. *CLF* represses expression of *AGL19* in an age-related manner (Schönrock et al. 2006). Additionally, *CLF* has been shown to control leaf morphogenesis (Kim et al. 1998).

We identified genes with highly negative coefficients for both traits. *TARGET OF EARLY ACTIVATION TAGGED (EAT) 2* (*TOE2*) is a highly negative predictor of both traits, suggesting that there is a link between leaf size, biomass, and the microRNA/SPL-mediated juvenile to mature transition (Werner et al. 2021). Other highly negative predictors of both traits include transcription factors controlling stomatal development (*TSO1-LIKE CXC DOMAIN–CONTAINING PROTEIN 1; SOL1*) and shade avoidance response (*PHYTOCHROME-INTERACTING FACTOR 7; PIF7*; Galvāo et al. 2019; Simmons et al. 2019).

We also sought to understand important predictors of 1 trait which were not predictive of the other trait. For leaf size, strongly positive predictors included: an ethylene response factor (ERF) TF family gene (*ETHYLENE RESPONSE FACTOR 18*; *ERF018*); a positive regulator of photomorphogenesis and regulator of flowering (*CONSTANS-LIKE 3, COL3*); and, as mentioned above, *AGL16* (Nakano et al. 2006; Tripathi et al. 2017; Liu et al. 2021). Conversely, *MYB-SHAQKYF 1* (*MYS1*), a transcription factor involved in leaf wax biosynthesis, was identified as a strongly negative predictor of leaf size but not biomass (Liu et al. 2022). For biomass, strongly positive predictors included: a basic region/leucine-zipper motif (bZIP) TF family gene, identified as affecting germination (*BASIC LEUCINE-ZIPPER 44; bZIP44*); *SQUAMOSA PROMOTER*–*BINDING PROTEIN-LIKE 4* (*SPL4*), which is repressed by *microRNA 156 (miR156)*; and *RGA-LIKE 1* (*RGL1*), which is a negative regulator of gibberellin responses (Wen and Chang 2002; Iglesias-Fernández et al. 2013; Xu et al. 2016). Conversely, strong negative predictors of biomass included *CLF* and *AGL19*, as mentioned above.

In addition, we used regularized logistic regression models to predict bolting status (see Materials and methods). Similar to biomass models, these models had slightly higher accuracy when trained on only regulators, rather all genes (Fig. 2D, Supplementary Fig. S4C).

### Single-plant-omics can generate a high-resolution time series

**Figure 3.**
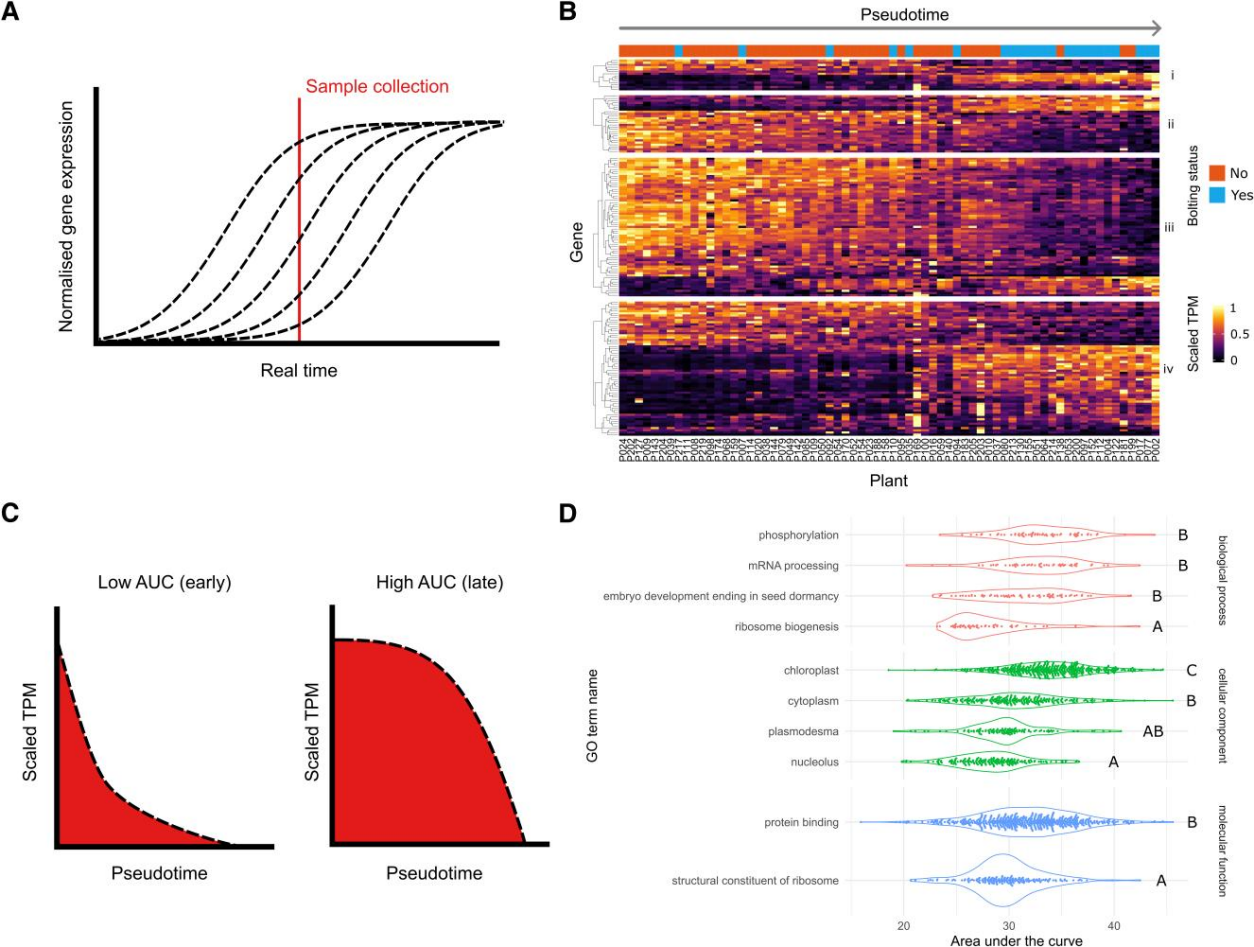
Pseudotemporal ordering of individual plants. **A)** A theoretical interpretation of our sampling method. The dashed curves represent gene expression from individual plants across real time since germination. The red line represents the time when all samples were collected. Due to the desynchronization of gene expression between plants, the red line captures a variety of gene-expression values across samples. **B)** A repeat of the heat map from Fig. 1, reordered over pseudotime and with a different scaling of TPM values (see the Pseudotime inference section). Gene-expression dynamics are mostly monotonic and smoothly changing. **C)** A diagram which demonstrates the AUC values, for genes with decreasing gene expression. Dashed lines represent the theoretical gene-expression values (after they have been scaled). The red region measures AUC. Genes whose expression decreases early on in pseudotime will have a low AUC value. Conversely, genes which decrease later on in pseudotime will have a high AUC value. **D)** AUC plots of key GO terms. These have been grouped by GO category (biological process, cellular component, and molecular function). Each dot represents a single gene annotated with that GO term. Letters depict significantly different GO terms based on Supplementary Data Set 12 (“kwAllPairsNemenyiTest” post hoc test, with the χ^2^ approximation, from the PMCMRplus v1.9.7 R package; Nemenyi 1963; Pohlert 2023). AUC, area under the curve; GO, gene ontology; TPM, transcripts per million.

The above results suggest that some genes are associated with continuous changes, such as determinants of biomass and leaf area. Other processes are associated with the “binary” shift in gene expression from nonbolted to bolted plants (Graphical Abstract). However, we expected that even a rapid developmental transition will involve continuous changes in gene expression near the transition point but observing these changes would require sampling many time points during the rapid transition period (Ezer and Keir 2019). Although the individual plants in our population were sampled at the same chronological age, they developed asynchronously. Thus, we hypothesized that individuals within a population of plants would demonstrate asynchronous gene-expression dynamics (Fig. 3A). Here, we introduce a new method to order the plants by their biological age, based solely on gene-expression data.

This problem of ordering samples based on gene-expression data alone is related to *pseudotime inference* in the analysis of single-cell RNA-seq data. In that context, “pseudotime” refers to a pseudotemporal ordering of single cells, which is assumed to contain information about biologically meaningful developmental trajectories, such as cell differentiation (Saelens et al. 2019; Ding et al. 2022). Although multiple methods exist for pseudotime inference, the most popular approaches require large numbers of samples (cells) due to their unsupervised approach (Saelens et al. 2019; Ding et al. 2022). Notably, our sample size of 68 plants is lower than the hundreds or thousands of cells required for unsupervised methods. To circumvent this restriction, we incorporated an assumption that the expression of most genes across the vegetative-to-reproductive transition was either monotonically increasing or decreasing. Our first basis for making this assumption was our earlier observation that the majority of genes are differentially expressed during bolting, suggesting that they primarily increase or decrease in expression (‘Widespread transcriptional changes occur after bolting in Arabidopsis thaliana’). Our second basis was that previous longitudinal studies of leaves show that many genes change their expression monotonically over development (Breeze et al. 2011; Woo et al. 2016). We were able to consistently order our plants along pseudotime to maximize the monotonicity of gene expression, using distinct subsets of genes. Finally, we compiled a consensus pseudotime of samples (Materials and methods; Supplementary Fig. S5).

We observed that pseudotime captures the sequence of changes in gene expression within bolting-related processes. We visualized genes associated with programmed cell death, regulation of cell cycle, photosynthesis, and response to jasmonic acid over pseudotime (Fig. 3B). Within each of these groups, there were clusters of genes with different expression timings, with changes in expression occurring earlier or later in the bolting process. We used a simple metric to describe whether genes were early or late changing: “area under the curve” (AUC; Fig. 3C; Materials and methods; Supplementary Data Sets 8 to 10). Note, this should be distinguished from the commonly used area under the receiver operating characteristic curve statistic, that is used to judge performance of diagnostic tests (Hanley and McNeil 1982). For further analysis, we selected the top genes that were monotonic and varied smoothly over pseudotime (see Materials and methods and Supplementary Fig. S6). Clustering of genes indicates that different expression dynamics, corresponding to different AUC values, are represented in the filtered dataset (Supplementary Figs. S7 and S8; Materials and methods). For the majority of genes, the residuals between the fit and the real data are consistent across pseudotime (Supplementary Fig. S9).

To better characterize the timing of different biological processes across the bolting transition, we tested for GO terms with significant differences between AUC values (Fig. 3D, Supplementary Figs. S10 to S12 and Data Sets 10 to 13; Materials and methods). To understand the order of processes shutting down during the onset of senescence, we focused on genes that decreased their expression over pseudotime. First, we found that ribosome biogenesis decreased earlier than processes related to phosphorylation, mRNA processing, and embryo development, leading to seed dormancy, suggesting that ribosomal production is slowed even before the onset of a visible bolt (Fig. 3D). Similarly, terms for different cellular components decreased in the following order: nucleolus, cytoplasm, and then chloroplast. Interestingly, the term plasmodesma decreases significantly earlier than chloroplast, suggesting that intercellular processes shut down before photosynthesis-related processes (Fig. 3D, Supplementary Data Set 12). Finally, we observed that mRNA binding decreases sooner than protein binding. These results suggest that ribosome production is one of the first processes to shut down at the onset of senescence, while photosynthesis is one of the last processes.

### Pseudotime assists in the inference of gene regulatory networks

**Figure 4.**
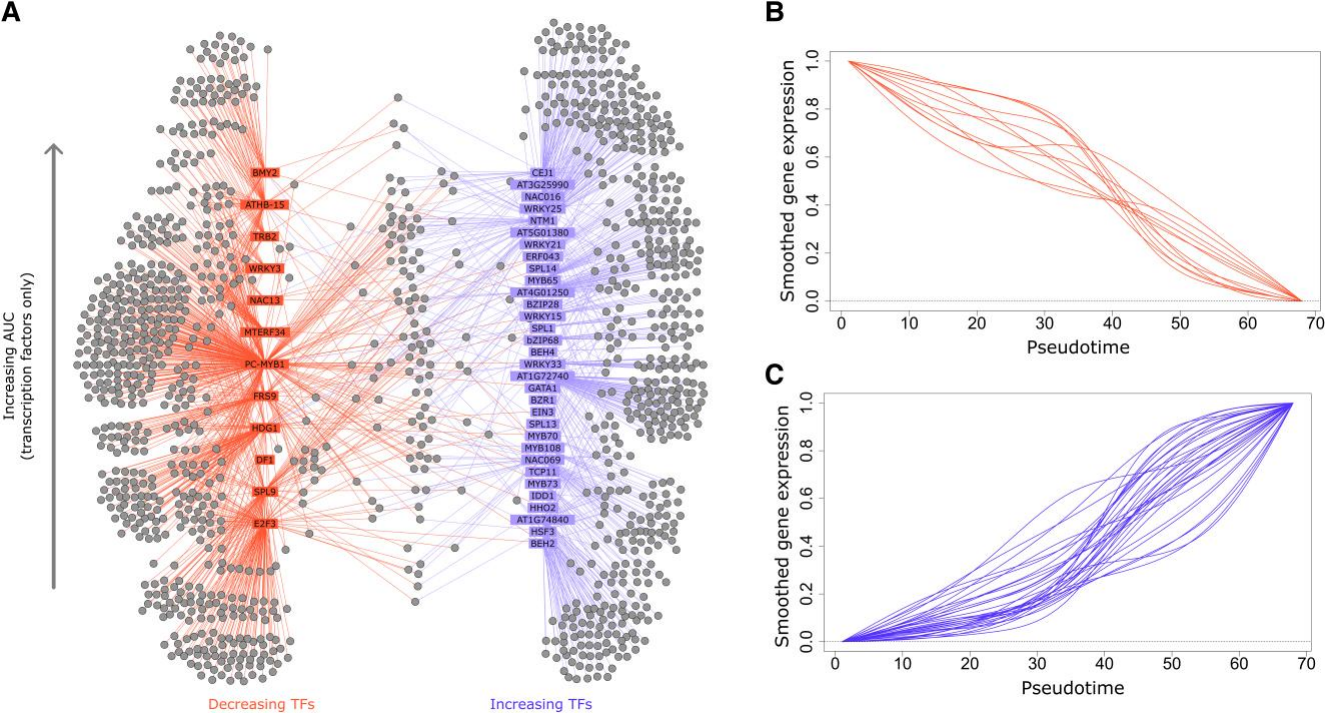
GRN based on pseudotemporal ordering. **A)** A visualization of the GRN predicted by DynGENIE3, after filtering with DAP-seq data (see main text). Decreasing regulatory transcription factors are colored in red, increasing ones in blue. Additionally, transcription factors are ordered bottom to top by increasing ranked AUC value. The gray dots represent predicted targets (genes) of the TFs. Each line indicates a predicted interaction from a TF to a target. **B)** The smoothed gene-expression values over pseudotime of decreasing TFs from the GRN. **C)** The smoothed gene-expression values over pseudotime of increasing TFs from the GRN. AUC, area under the curve; GRN, gene regulatory network; TF, transcription factor.

Our next aim was to identify the regulatory cascade that led to the large-scale transcriptional changes during bolting. We inferred gene regulatory networks (GRNs) from the gene-expression data over pseudotime (Fig. 4A, Supplementary Data Set 14 and Fig. S13). To enrich for direct regulatory interactions, we filtered for potential regulatory links that were validated by DAP-seq (O’Malley et al. 2016; see Materials and methods). We only included transcription factors in our final network whose expression changed smoothly over pseudotime (see the Pseudotime inference section) and that had DAP-seq data available for validation. The transcription factors in our network displayed varied expression patterns over pseudotime (Fig. 4, B and C), and they are ordered by their AUC in the network in Fig. 4A. We identified 4 members of the SQUAMOSA PROMOTER–BINDING PROTEIN-LIKE (SPL) family as potential regulators in our GRN. Two of these genes (*SQUAMOSA PROMOTER*–*BINDING PROTEIN-LIKE 9*, *SPL9*, and *SQUAMOSA PROMOTER*–*BINDING PROTEIN-LIKE 13B*, *SPL13B*) are known to be targeted by miR156 during the vegetative-to-reproductive transition (Xu et al. 2016). We found that 15 out of 44 regulators identified in the GRN analysis were also found to be differentially expressed within a mature-to-senescent time series of leaf development (Woo et al. 2016; Supplementary Data Sets 9 and 14). These regulators included members of the NAC (NAM, ATAF1/2, and CUC2) and WRKY transcription factor families: *NAC DOMAIN–CONTAINING PROTEIN 13* (*NAC13*), *NAC DOMAIN–CONTAINING PROTEIN 16 (NAC016*), *NAC WITH TRANSMEMBRANE MOTIF1* (*NTM*), *WRKY DNA*–*BINDING PROTEIN 15* (*WRKY15*), and *WRKY DNA*–*BINDING PROTEIN 25* (*WRKY25*) (Olsen et al. 2005; Phukan et al. 2016).

### A small number of genetic variants are associated with pseudotime

Developmental asynchrony can be caused by environmental and genetic factors, as well as random stochasticity. Even in a nearly isogenic wild-type population, there will be a number of genetic variants. Within our population, we identified a total of 1,047 high-confidence variants (see Materials and methods for details on variant calling, Supplementary Data Set 15). We observed that a low amount of genetic variation was explained by principal component analysis (PCA) (Supplementary Fig. S14), highlighting the complex population structure. The analysis of a neighbor-joining tree, trained on variants, showed that bootstrap trees had low agreement with the original tree (Supplementary Fig. S15A). An alternative method suggested that 2 subgroups may be present in the population based on the variant data (Supplementary Fig. S15B). The seeds used in this experiment are the progeny of 15 different plants and those parents likely constitute 2 or more lineages with fixed nucleotide changes. By fitting linear models linking these 2 subgroups of samples with gene expression, we found that 13,545 genes had an association with the variant-based subgroups of samples (adjusted *P*-value <0.05; Supplementary Fig. S14 and Data Set 16). This is consistent with the facts that the subgroups largely separate out bolted and nonbolted plants and that 55% of the transcriptome is differentially expressed between the bolted and nonbolted plants. This analysis also suggests that there is a potential for false-positive gene expression–trait associations that result from multiple lineages with different fixed variants present within the experiment.

**Figure 5.**
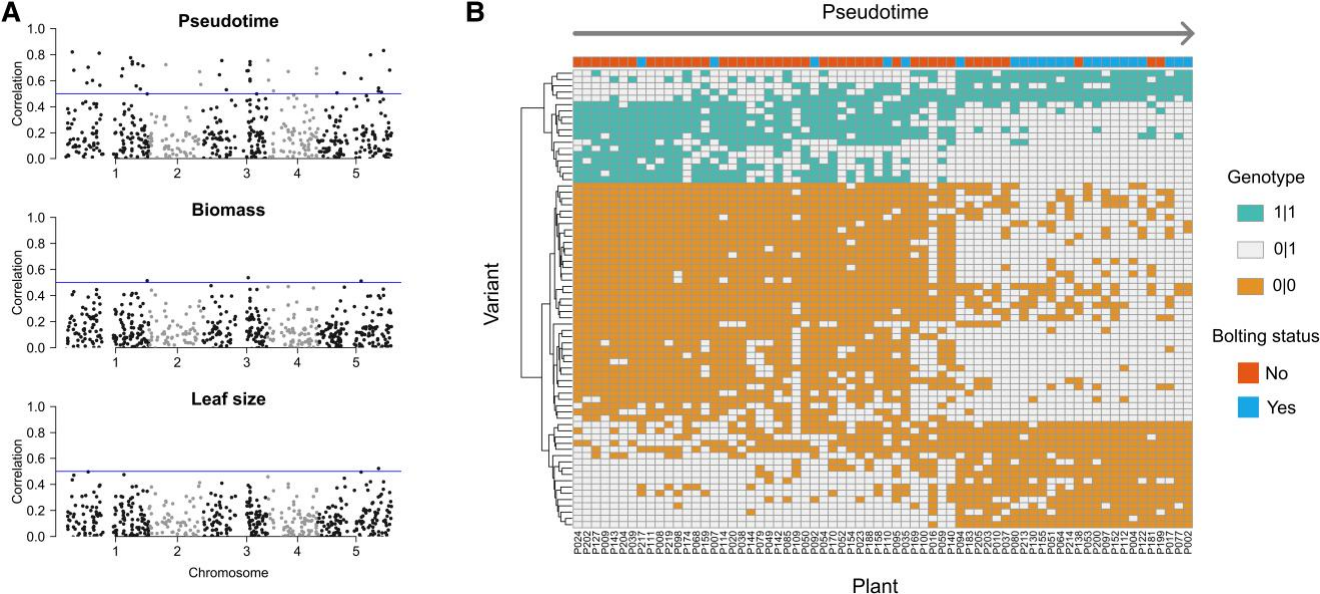
Comparison of variants to pseudotime and physiological traits. In this figure, 0|0 corresponds to homozygous for the reference allele, 0|1 corresponds to heterozygous, and 1|1 corresponds to homozygous for the mutant allele. **A)** Correlation of variants to pseudotime, biomass, and leaf size. Correlation was calculated by assigning to 0 to 0|0, 1 to 0|1, and 2 to 1|1, then computing the absolute value of Pearson correlation between genotype and the measurement of interest. The blue line corresponds to a correlation of 0.5. *n* = 68 plants shown, *n* = 1,047 variants shown. **B)** Genotypes of variants with a high correlation (>0.5) to pseudotime. Columns (plants, *n* = 68) are ordered by increasing pseudotime. Rows (variants, *n =* 73) are ordered by hierarchical clustering.

We next sought to understand whether variants could be linked to pseudotime or physiological traits. Interestingly, there were more variants that were highly correlated (absolute correlation > 0.5) with pseudotime than with either of the complex physiological markers (Fig. 5A). The variants that were most correlated to pseudotime were not co-localized across the genome, indicating that they were not part of the same linkage disequilibrium block. In addition, we trained models to predict physiology on variant data, to judge whether the variants were more or less informative than the gene-expression data. The variant-based Elastic Net models for biomass and leaf size performed worse on both *R*^2^ and RMSE metrics (Fig. 2, Supplementary Figs. S4 and S16). The variant-based logistic regression model for bolting status performed slightly worse (76.5% accuracy) than the logistic regression models that used the full transcriptome (80.8% accuracy) or the transcriptome of regulatory genes (85.2% accuracy; Fig. 2, Supplementary Figs. S4 and S16). Since nonbolted plants are mostly earlier in pseudotime compared with bolted plants, this result is consistent with our observation that a small number of variants (73 out of 1047) correlate strongly with pseudotime (absolute Pearson correlation >0.5) (Fig. 5B). Furthermore, the presence of these variants helps to explain why unbolted plants cluster with bolted plants and vice versa. These results further validate the biological relevance of pseudotime, by indicating that genetic variants are more closely associated with it than with complex measured traits, such as biomass and leaf size.

## Discussion

Single-plant-omics enabled us to identify genes associated with the binary developmental transition and gradual developmental traits. More than half of all Arabidopsis genes significantly change their expression levels at the onset of bolting. By increasing the number of biological replicates, our single plant-omics approach allows us to detect many more differentially expressed transcripts than in previous studies (Woo et al. 2016). In addition, we were able to identify a distinct set of transcriptional regulators that were predictive of leaf size and biomass accumulation. These included members of the AGAMOUS-LIKE MADS-box transcription factor family, whose orthologs in rapeseed have been highlighted as predictors of yield phenotypes in a recent single-plant-omics study (De Meyer et al. 2023). These results suggest that single-plant-omics can help us distinguish between the transcriptional processes associated with fast and slow developmental processes.

However, even though developmental transitions are rapid, the transcriptional regulation that occurs during these transitions is governed by biochemical processes that will change smoothly over time. One of the main benefits of the single-plant-omics approach is that it enables us to piece together the sequence of changes in what initially appears to be a switch-like transition process. We developed a new method of ordering individual plants by their biological age, which we refer to as their pseudotime. We could consistently identify an ordering of plants when utilizing independent subsets of genes, removing the need for marker gene selection (Meir et al. 2021). In addition, we show that it is biologically meaningful because genetic variants in the population of wild-type plants are more closely associated with pseudotime than they are to measured traits like biomass and leaf area. While other methods for pseudotime inference are widely used (Saelens et al. 2019; Ding et al. 2022), these are designed for single-cell RNA-seq datasets with hundreds or thousands of cells, while our method is effective for smaller sample sizes.

By using pseudotime, we were able to dissect the order of events that took place during bolting at a higher resolution than other studies that relied on RNA-seq time series (Breeze et al. 2011; Woo et al. 2016). Although it is well known that the timing of bolting is closely associated with leaf senescence in different Arabidopsis accessions and mutants (Balazadeh et al. 2008; Upadhyay et al. 2014; Yan et al. 2017), here we show that many senescence-related processes are concurrent with bolting. Indeed, some of the early processes associated with senescence, such as shutting down ribosome production, appear at a transcriptional level before the visible onset of bolting, which challenges preconceived notions about the order of events that occur during bolting. We identified 43 key transcriptional regulators and identified the order in which they increase or decrease their expression during the vegetative-to-reproductive transition. Our resulting gene network will be a valuable resource for exploring the regulation underpinning this fundamental developmental transition.

We suggest that single-plant-omics provides numerous benefits for the understanding of rapid developmental transitions, such as (i) increased sensitivity to DEGs, (ii) the ability to distinguish between gene expression changes associated with fast and slow developmental processes, and (iii) the capacity to reconstruct the sequence of transcriptional events that occur during a rapid developmental process. Single-plant-omics coupled with pseudotime inference will be widely applicable to investigate other rapid developmental processes, such as germination, the juvenile-to-vegetative transition, and floral development.

Our work also highlights that near-isogenic lab strains may still contain genetic variants that are associated with traits of interest, suggesting that experiments need to be designed to robustly handle heterogeneity between individual plants. The complex population structure of near-isogenic lines of laboratory strains warrants further consideration by the research community.

## Materials and methods

### Plant growth conditions, library preparation, and RNA-seq

Seeds from the Arabidopsis (*A. thaliana*) ecotype Wassilewskija (Ws-2) (Anwer et al. 2020) came from a collection of ∼15 parents. These were surface sterilized and plated onto 1 × Murashige and Skoog basal salts (Duchefa Biochemie) supplemented with 1% w/v sucrose (Thermo Fisher), 0.5% w/v MES (Melford Bioscience), and 1.5% w/v phytoagar (Duchefa Biochemie). After 4 d of stratification at 4 °C, plates were transferred to long-day photoperiods (16 h light/8 h dark), next to vertical, cool white, fluorescent lights (Osram Cool White 16W/840) with a light intensity of 50 *µ*mol m^−2^ s^−1^ and a constant temperature of 21 °C for 10 d. On Day 10, 225 seedlings were individually transferred to soil (F2 + 5% sand, Levington) and grown for a further 10 d under a long-day photoperiod (16 h light/8 h dark), under cool white, fluorescent top lighting (Phillips Master TL-D 36W/840) at an intensity of 70 *μ*mol m^−2^ s^−1^ with a constant temperature of 21 °C. Throughout growth, plants were shuffled within the growth chambers approximately every 3 d. The 3rd and 4th true rosette leaves were tracked during growth. On Day 20, 75 plants were selected from the population to ensure a diversity of bolting statuses. For each of these 75 plants, they were classified as bolting if there was a visible bolt ∼1 cm above the rosette, and not bolting otherwise. Images of the rosettes were taken at a consistent height. Five plants were not sequenced since one or more leaves were not visible in the relevant image. On Day 21 at ZT4 (4 h after lights on), the 3rd and 4th leaves from individual plants were harvested together, pooled, and snap-frozen in liquid nitrogen. The rosette biomass (not including the 3rd and 4th leaves) was then weighed (Supplementary Data Set 2). Later, the mean area of the 3rd and 4th leaves was calculated using the Polygon selection tool in Fiji with a ruler used for scale (Schindelin et al. 2012; Supplementary Data Set 2). Throughout this work, we refer to each plant individually (e.g. “P002”)—since gene expression and physiological measurements were measured separately per plant.

Total RNA was isolated from leaf tissue using the Qiagen RNeasy Plant Mini Kit (Cat no. 74904). Residual genomic DNA was removed using the Invitrogen Turbo DNA-free kit (Cat no. AM1907), according to the manufacturer's protocol. Libraries for RNA sequencing were prepared with the NEBNext Ultra II Directional Library Prep Kit for Illumina (Cat no. E7765), using the NEBNext poly(A) magnetic isolation module (Cat no. E7490). Quality control was performed with the Agilent 2100 Bioanalyzer instrument (Part no. G2939BA). Finally, a total of 70 bar-coded libraries were pooled and sequenced, via Novagene, using 1 lane on an Illumina NovaSeq system. Before analysis of the raw sequencing data, FastQC v0.11.7 (Andrews 2010) was used to assess read quality. Illumina adapters were trimmed using CutAdapt v3.4 (Martin 2011). Reads were quantified using Salmon v1.6.0 (Patro et al. 2017) and the TAIR10 transcriptome (Berardini et al. 2015).

For further analysis except differential gene expression, transcripts per million (TPM) was used as the measure of relative gene expression across samples. Additionally, TPM levels per transcript isoform were combined to leave only gene-level expression data. One sample (P158) was sequenced again due to low read depth in the initial sequencing, and the initial data were discarded. Two samples (P128 and P196) were discarded as outliers during initial clustering of the data (Supplementary Fig. S1). Finally, TPM values were filtered to remove genes with very low expression, to avoid biassing clustering and other analyses. Genes were not considered for further analysis if they met either of these 2 criteria: (i) TPM of 0 in 10 or more samples, or (ii) TPM below 0.5 in all samples. This left 19,283 genes for further analysis. After this filtering, samples clustered into 2 large groups (Supplementary Fig. S2).

### Differential gene expression and GO term overrepresentation

Due to the large number of samples, we used the nonparametric Mann–Whitney *U* test to perform differential gene-expression analysis, as recommended by Li et al. (2022). However, since we expected >30% of the transcriptome to be differentially expressed, we did not perform the trimmed mean of *M* values normalization (Robinson and Oshlack 2010). Counts per million (CPM) was used as input to this test, and *P*-values were corrected according to the Benjamini and Hochberg method to control the False Discovery Rate (Benjamini and Hochberg 1995). Salmon produces “NumReads” as an estimate of the number of reads corresponding to a transcript, so these values were combined to the gene level (as above) and then normalized to produce CPM values (Patro et al. 2017). Genes were classified as differentially expressed if both of the following conditions were true: (i) the adjusted *P*-value was <0.05, and (ii) the log_2_-fold change between the means with the nonbolted and bolted plants was >0.1 or <−0.1 (Supplementary Fig. S3 and Data Set 3). GO term overrepresentation was performed using the gprofiler2 R package (version 0.2.1; Kolberg et al. 2020). This called the g:Profiler server (version e111_eg58_p18_30541362), which utilizes the g:SCS multiple testing correction method, and we then applied a significance threshold of 1e−4 (Kolberg et al. 2023; Supplementary Data Set 4).

### Physiology trait prediction with elastic nets

Due to the large number of possible predictors (i.e. genes) vs. outputs (i.e. biomass and leaf area), we needed to regularize the linear regression models and logistic regression models used in prediction. The aim of this was to remove the many low-impact predictors, whose individual effect on the model was very small. We chose the elastic net as a regularization method (Zou and Hastie 2005). TPM values were *z*-scored before input. A set of known and potential regulatory genes was curated by selecting genes annotated with the following GO terms: “DNA-binding transcription factor activity” (GO:0003700), “signal transducer activity” (GO:0004871), and “regulation of transcription—DNA templated” (GO:0006355), which were the same as those in a previous single-plant-omics publication (Cruz et al. 2020). These were used as the set of possible predictors for all figures in the main text.

Since the elastic net requires predetermined settings to balance regularization with prediction accuracy, we used a tiered cross-validation approach to find optimal hyperparameters. This was implemented using “scikit-learn” in Python v3.10.4 using the “ElasticNetCV” function for leaf and biomass models and “LogisticRegressionCV” for bolting models (Pedregosa et al. 2011). First, LOOCV was used to produce a 68 separate test-train splits, where the testing set only consisted of a single sample (Hastie et al. 2009). Then, for each training set, 5-fold cross-validation was used to pick optimal “alpha” and “l1_ratio,” with possible inputs for “l1_ratio” of 0.1, 0.5, 0.7, 0.9, 0.95, 0.99, and 1.0 (Supplementary Data Set 5). Parameters relating to “alpha” (“ElasticNetCV”) were kept at their default values. For logistic models, 10 different “C” values were tested, by setting “Cs” to 10 in “LogisticRegressionCV.” The output of this process was a set of optimal hyperparameters and a trained elastic net model for each test-train split (Supplementary Data Set 5). Finally, we needed to select a single “l1_ratio” value to apply across every leaf and biomass model, since this value controls the amount of variable selection and we wanted to compare the selected predictors (Fig. 2C). We chose an “l1_ratio” of 0.5 for these since this was the most common value across all LOOCV folds in leaf size models with all genes and leaf size models with regulators only (Supplementary Data Set 5). For bolting models, the “l1_ratio” was set to the mode value selected in the hyperparameter tuning process (0.5 for models trained on all genes, and 0.1 for models trained on regulatory genes only). The predictions and coefficients from optimal models, with fixed “l1_ratio” values across folds, are summarized in Supplementary Data Sets 6 and 7, respectively.

### Pseudotime inference

Since our sample size was too small to infer an ordering of samples using unsupervised techniques, which is typical in single-cell RNA-seq data analysis (Saelens et al. 2019; Ding et al. 2022), we developed a new pseudotime inference method. We applied the following steps to genes identified as differentially expressed (see above). First, we processed the gene-expression values for each gene as follows: (i) *z*-score TPM values per gene and truncate any values >3 or <−3; (ii) linearly scale these values, so that the minimum value was 0 and maximum value was 1 for each gene; and (iii) if the gene was identified as downregulated, change the gene expression after step (ii), *x* to be 1−x. To validate this assumption, we partitioned genes into 100 distinct groups and repeated the pseudotime inference method for each group (Supplementary Fig. S5). Specifically, define $x_{i,j,k}$ as the normalized expression of the *i* th gene in group *j* in the *k* th sample, where j=1,…,100, $i\;=\;1,\ldots ,n_{j}$, and k=1,…,68. Then, for each group *j*, the *k* samples were ordered based on $\sum_{i=1}^{n_{j}}x_{i,j,k}$, i.e. the sum of normalized expression of every gene within a fixed group and sample. Finally, we combined the individual predicted pseudotemporal orderings, by assigning each sample to its most common predicted position between the separate orderings.

Following pseudotime inference, gene-expression values were smoothed and filtered based on the goodness of fit of the smoothed curves. Cubic B splines were used as basis functions to fit to the data and the smoothing process was penalized with the second derivative. The fitting process was repeated, varying the number of basis functions and the smoothing parameter (“lambda”), to choose optimal hyperparameters, i.e. those that minimized the sum of the generalized cross-validation (GCV) across all genes (Kokoszka and Reimherr 2017; Supplementary Fig. S6A and Data Set 8). Finally, the 4,000 genes with lowest GCV were chosen for further analysis (Supplementary Fig. S6B and Data Set 9). The smoothed functions were again rescaled to ensure they had a minimum of 0 and maximum of 1, allowing for comparisons between time series. Additionally, some genes had a few samples where the TPM was much higher than in other samples, and these genes had inappropriately smoothed curves. Genes were, therefore, removed if the ratio between their range and their interquartile range was too high (Supplementary Figs. S6, C and D and Data Set 9). A total of 3,906 genes remained after these filtering steps.

### Analysis of gene expression over pseudotime

We summarized the expression dynamics of each monotonically increasing or decreasing gene by its AUC. First, we filtered for genes whose smoothed expression was monotonic over pseudotime. Specifically, a gene was considered monotonic if the derivative of its B-spline-fitted expression over pseudotime (see the Pseudotime inference section) never crossed 0. (Due to the potential for numerical errors when calculating the derivative, we set all derivative values within ±1e−3 to 0, to avoid spuriously removing monotonic genes.) Then, we summarized these genes by calculating their AUC—i.e. the integral of the curve between the start and end of pseudotime. Crucially, since these selected genes are monotonic, a gene with smaller AUC changes more rapidly earlier on in pseudotime (see Fig. 3C). For increasing genes, the AUC was calculated for (1 − normalized gene expression) values, so that a smaller AUC also indicated changes earlier on in pseudotime.

We clustered the monotonic genes using a shape-based metric from dtwclust (version 5.5.12), using the “tsclust” function with “distance” set to “sbd” (Sardá-Espinosa 2019). This is an implementation of the *k*-shape algorithm (Paparrizos and Gravano 2016). To select an appropriate number of clusters of visualization, we calculated the within cluster sum of squares, which is a metric to evaluate the tightness of clusters. This can be calculated using $\sum_{i=1}^{k}\;\sum_{g\in C_{i}}\;d(g,\;\mu_{i}{)}^{2}$, where *k* is the number of clusters, $C_{i}$ is the set of genes in cluster *i*, $\mu_{i}$ is the centroid of $C_{i}$, and $d(g_{1},\;g_{2})$ is the distance between $g_{1}$ and $g_{2}$ as calculated by the *k*-shape algorithm. We then selected k=25 as the optimal number of clusters, after repeating the algorithm 5 times for values of k=2,3,…,50 (Supplementary Fig. S7).

After we calculated AUC values, we chose to group genes by GO term, to find which GO terms had significantly different average AUC values. We used the nonparametric Kruskal–Wallis test to see whether there were any significant differences between GO terms, i.e. if the *P*-value was <0.01. We compared GO terms which had similar numbers of genes (Supplementary Data Set 11 and Fig. S10), to avoid comparing very detailed GO terms with fewer labeled genes (such as “cellular response to DNA damage stimulus”) with broader GO terms (such as “translation”). This was then followed by a nonparametric post hoc test to find significantly contrasting GO terms, i.e. where the corrected *P*-value was <0.01. Specifically, we used the command “kwAllPairsNemenyiTest,” with the χ^2^ approximation, from the PMCMRplus v1.9.7 R package (Nemenyi 1963; Pohlert 2023). Adjusted *P*-values (via the “single-step” method) are summarized for every test in Supplementary Data Sets 12 and 13. Additionally, we visualized the distribution of AUC values for all GO terms within the same size boundaries (Supplementary Figs. S11 and S12).

### GRN analysis

After pseudotime inference and filtering for monotonic genes, we applied a gene regulatory inference method (DynGENIE3) designed for time-series gene-expression data (Huynh-Thu and Geurts 2018). Since this method required a prespecified list of potential regulatory genes, we selected TFs included in a previous DAP-seq experiment (O’Malley et al. 2016). In addition, we filtered for TFs which passed pseudotime filtering (see the Pseudotime inference section). This resulted in a potential 44 transcription factors. After running DynGENIE3, we kept only edges with a predicted weight of at least 1e−10, and then further selected the top 5% of all edges by weight (Supplementary Fig. S13).

To select for high-quality predicted interactions, we filtered the results against the DAP-seq dataset (O’Malley et al. 2016). Specifically, the putative regulatory targets (with FRiP ≥5%) were downloaded for all sequenced TF-binding sites. This was retrieved on June 1, 2023 from http://neomorph.salk.edu/dap_web/pages/browse_table_aj.php. Overall, this GRN included 1,668 genes and 2,248 interactions (Supplementary Data Set 14).

For graph visualization, Gephi v0.9.7 was used (Bastian et al. 2009). Note that for Fig. 4, not all TFs were monotonic over pseudotime (see the Analysis of gene expression over pseudotime section). Therefore, a TF was classified as “decreasing” if the initial value of the smooth curve over pseudotime was greater than the final value and classified as “increasing” otherwise.

### Variant calling

To analyze genetic variation within the population, we followed GATK guidelines for short variant discovery from RNA-seq data. We used STAR (v2.7.10b; 2-pass mode) to align reads to TAIR10 genome, revision 56 (Dobin et al. 2013). We then preprocessed the aligned reads using the commands “MarkDuplicates” and “SplitNCigarReads” from GATK (v4.3.0.0) (Poplin et al. 2018). We used the command “HaplotypeCaller” to produce genomic variant calling format (gVCF) files per sample. Finally, we produced identified single-nucleotide variants (SNVs) and insertions/deletions (indels) which were confidently called across the whole population, using “GenotypeGVCFs.”

For further processing of these initial SNVs and indels, we followed the filtering guidelines suggested by Cruz et al. (2020). Specifically, we selected only biallelic variants with a minimum genotype quality of 40, and which were called in at least 80% of all samples, using VCFtools (v0.1.16; Danecek et al. 2011). We then imputed missing genotypes using Beagle (v5.4, 22Jul22, 46e) on default settings (Browning et al. 2018). Finally, we selected variants with a minor allele frequency of at least 0.05. Note, due to preprocessing by Beagle, the VCF file includes 2 versions of the same heterozygous haplotype (“0|1” and “1|0”; Browning et al. 2018; Supplementary Data Set 15). These were combined into a single heterozygous haplotype, and any variants with only heterozygous haplotypes across all samples were removed.

For analysis by PCA and as inputs to elastic net and logistic regression models, each haplotype was converted into an integer: the homozygous reference allele was assigned 0; the heterozygous allele was assigned 1; and the homozygous alternate allele was assigned 2. Elastic Net and logistic regression models were trained using cross-validation, as explained in the Physiology trait prediction with elastic nets section, except the hyperparameter “l1_ratio” was allowed to vary to maximize prediction accuracy. The neighbor-joining algorithm was performed using the ape package (version 5.7-1) in R, using the commands “nj,” “dist.gene” to count the number of differences in variants between samples after representation as integers, and “boot.phylo” (with “*B* = 1,000”) to repeat the analysis using bootstrapping (Efron et al. 1996; Paradis and Schliep 2019). A second analysis of population structure was performed using the SNPRelate package (version 1.32.2) in R, using “snpgdsIBS” to calculate the Identity-by-state distance matrix, “snpgdsHCluster” to perform hierarchical clustering, and “snpgdsCutTree” to determine an appropriate number of subgroups using a permutation test (Zheng et al. 2012).

To assess the relationship between the suggested population subgroups and gene expression, we ran linear models to predict log-transformed gene expression (i.e. $\log_{2}\;(x+1)$, where *x* is measured as TPM) from the subgroups of samples, based on variants (Supplementary Data Set 16). This closely follows the linear mixed effects models used to assess the effect of population structure in Cruz et al. (2020), except that we did not need to account for effects such as harvest date, sequencing batch, or spatial autocorrelation. We separated out the 2 subgroups, based on the 2 large clusters from the variant-based PCA (Supplementary Fig. S14). Note, these subgroups were consistent with the 2 subgroups predicted by the SNPRelate analysis (Supplementary Fig. S15B). We adjusted the *P*-values for the associated *t*-tests, using the Benjamini and Hochberg method to control the false discovery rate (Benjamini and Hochberg 1995).

### Accession numbers

Sequence data from this article can be found in the GenBank/EMBL data libraries under the following accession numbers: HB54 (AT1G27045), IAA10 (AT1G04100), SEP4 (AT2G03710), AGL16 (AT3G57230), AGL19 (AT4G22950), CLF (AT2G23380), TOE2 (AT5G60120), SOL1 (AT3G22760), PIF7 (AT5G61270), ERF018 (AT1G74930), COL3 (AT2G24790), MYS1 (AT2G38300), bZIP44 (AT1G75390), SPL4 (AT1G53160), RGL1 (AT1G66350), SPL9 (AT2G42200), SPL13B (AT5G50670), NAC13 (AT1G32870), NAC016 (AT1G34180), NTM (AT4G01540), WRKY15 (AT2G23320), WRKY25 (AT2G30250).

## Supplementary Material

koae226_Supplementary_Data

## Data Availability

Raw and processed sequencing data have been deposited in the NCBI Gene Expression Omnibus database under accession number GSE242681 (https://www.ncbi.nlm.nih.gov/geo/query/acc.cgi? acc=GSE242681). Scripts used to produce the figures have been deposited in a GitHub repository (https://github.com/stressedplants/SinglePlantOmics).

## Supplementary data

The following materials are available in the online version of this article.

**Supplementary Figure S1.** Plots related to filtering of the RNA-seq dataset.

**Supplementary Figure S2.** Clustering of samples, based on correlation of log-scaled TPMs.

**Supplementary Figure S3.** Volcano plot to show differentially expressed genes, based on the bolting status phenotype.

**Supplementary Figure S4.** Performance of elastic net and logistic regression models trained on all genes.

**Supplementary Figure S5.** Summary of the bootstrapping process used to infer pseudotime.

**Supplementary Figure S6.** Selections of hyperparameters and filtering of genes after B-spline smoothing.

**Supplementary Figure S7.** Selection of number of clusters for Supplementary Figs. S8 and S9.

**Supplementary Figure S8.** Overall visualization of gene expression over pseudotime, separated into 25 clusters.

**Supplementary Figure S9.** Examples of genes from each cluster.

**Supplementary Figure S10.** Selection of GO term sizes.

**Supplementary Figure S11.** Area under the curve values for GO terms. Filtered to only include genes which decrease over pseudotime.

**Supplementary Figure S12.** Area under the curve values for GO terms. Filtered to only include genes which increase over pseudotime.

**Supplementary Figure S13.** Edge weights of predicted gene–gene interactions by DynGENIE3.

**Supplementary Figure S14.** Variant-based PCA.

**Supplementary Figure S15.** Two approaches of variant-based tree building.

**Supplementary Figure S16.** Elastic net and logistic regression models trained on variants.

**Supplementary Data Set 1.** Processed RNA-seq values.

**Supplementary Data Set 2.** Physiology data.

**Supplementary Data Set 3.** Differentially expressed genes.

**Supplementary Data Set 4.** g:Profiler results.

**Supplementary Data Set 5.** Hyperparameters for physiology models.

**Supplementary Data Set 6.** Predictions from optimal models.

**Supplementary Data Set 7.** Coefficients in optimal models.

**Supplementary Data Set 8.** GCV values with different hyperparameters.

**Supplementary Data Set 9.** Values for filtering out genes, based on smoothing.

**Supplementary Data Set 10.** AUC values.

**Supplementary Data Set 11.** Manually defined thresholds for the sizes of GO terms.

**Supplementary Data Set 12.** Statistics for decreasing GO terms.

**Supplementary Data Set 13.** Statistics for increasing GO terms.

**Supplementary Data Set 14.** Link list for the GRN.

**Supplementary Data Set 15.** Variant calling file.

**Supplementary Data Set 16.** Statistics for linear models based on variant subgroups.
