# Supplementary material for: Single-plant-omics reveals the cascade of transcriptional changes during the vegetative-to-reproductive transition
Source: Plant Cell. 2024 Aug 9;36(10):4594–606. doi: 10.1093/plcell/koae226 (PMC11449079; doi:10.1093/plcell/koae226)
Supplement: koae226_Supplementary_Data [file koae226_supplementary_data.zip › Supplementary Material Singleplantomics Redmond et al 2023 The Plant Cell Revised Jul 24 Final.pdf]

Supplementary Material: Single-plant-omics reveals the cascade of transcriptional changes during the vegetative-to-reproductive transition

Authors: Ethan J. Redmond[1], James Ronald[1, 2], Seth J. Davis[1], Daphne Ezer[1*]

1: Department of Biology, University of York, Wentworth Way, Heslington, York, YO10 5DD
2: School of Molecular Biosciences, College of Medical, Veterinary and Life Sciences, University of Glasgow, Glasgow, G12 8QQ
*: Corresponding author, daphne.ezer@york.ac.uk

Summary of Supplementary Data Sets

Supplementary Data Set S1 – Processed RNA-seq values

Each Excel sheet contains information about gene expression values, where rows indicate genes labelled by TAIR codes and columns indicate the samples. In summary:

- 'TPM unfiltered' is the summary of the predicted transcripts per million (TPM) values from Salmon, after being summed across isoforms per gene (see Plant growth conditions, library preparation, and RNA-seq in Methods).
- 'TPM filtered' contains the same values, but only for genes which passed initial filtering (Supplementary Figure S1).
- 'Normalized – pseudotime' contains the normalized TPM values for pseudotime inference and the columns are ordered by increasing pseudotime (see Pseudotime inference in Methods).

Supplementary Data Set S2 – Physiology data

Physiological measurements for individual plants. For leaf size, the mean value was taken for the 3rd and 4th true rosette leaves and the units are mm^2. For biomass, the units are mg.

Supplementary Data Set S3 – Differentially expressed genes

This contains all the genes identified as differentially expressed (see "Differential expression and GO term overrepresentation" in main text). The columns are:

- id = the TAIR code for the gene
- log2FoldChange = the log2-transformed fold change (i.e. mean of gene expression within bolted plant divided by the mean within non-bolted plants)
- p_adj = the adjusted P value for the Wilcox text between non-bolted and bolted plants
- diffexpressed = "up" if the gene is up-regulated and "down" if the gene is down-regulated

- gene_name = the name of the gene as found by biomaRt

Supplementary Data Set S4 – gprofiler results

This data set contains results from gProfiler (Kolberg et al. 2020) to find overrepresented GO terms within UP and DOWN groups.

Supplementary Data Set S5 – hyperparameters for physiology models

We used a hierarchical cross-validation approach to validate our models to predict physiological data from gene expression data (see “Physiology trait prediction with elastic nets” in Methods for details). This data set contains the optimal parameters chosen within each fold of the LOOCV, after each sample was individually removed from the training data. The different Excel sheets contain these values for the nine different classes of model (leaf size, biomass, and bolting status; predicted by all gene expression, with only regulatory genes, or variants).

Supplementary Data Set S6 – Predictions from optimal models

Similar to data set S5, these contain the predictions for each of the optimal models chosen in each fold of the LOOCV. Again, different sheets contain these values for the nine different classes of model.

Supplementary Data Set S7 – coefficients in optimal models

Similar to data set S5, these contain the coefficients and summary statistics for each of the optimal models chosen in each fold of the LOOCV. Again, different sheets contain these values for the nine different classes of model.

Supplementary Data Set S8 – generalized cross-validation (GCV) values with different hyperparameters

This data set contains the sum of GCV across all genes, when fitting B-splines with varying numbers of basis functions and applying a different level of regularization (“Pseudotime inference” in Methods; also visualized in Supplementary Figure S6A). The optimal hyperparameters chosen were: 9 basis functions and a regularization parameter of 1389.495, since these values produced the lowest sum of GCV across all genes.

Supplementary Data Set S9 – values for filtering out genes, based on smoothing

This data set contains values which were used to filter for genes which varied smoothly over pseudotime (“Pseudotime inference” in Methods). ‘gcv_value’ corresponds to the GCV of the B-spline fit to each gene (visualized in Supplementary Figure S6B). ‘iqr_value’ corresponds to the ratio between the range and interquartile range of the scaled TPM data (not the smoothed values; visualized in Supplementary Figure S6C). ‘woo_differentially_expressed’ is: TRUE if any isoform for this gene was identified as differentially expressed during the M-to-S transition in (Woo et al. 2016), i.e. if any isoform had the value ‘DET’ in column ‘M-to-S DET’ from Supplementary Table 3 from this reference; and ‘FALSE’ if all isoforms were not differentially expressed, or if it did not appear in Supplementary Table 3. ‘regulator_in_GRN’ is TRUE if this

gene was included as a potential regulator for the DynGENIE3 algorithm and FALSE otherwise.

Supplementary Data Set S10 – area under the curve (AUC) values

This data set contains 'area under the curve' values for all genes that passed GCV and IQR filtering. It includes a column 'AUC_value' for this value, and 'monotonicity' is either: increasing, decreasing, or nonmonotonic (see 'Analysis of gene expression over pseudotime').

Supplementary Data Set S11 – Manually defined thresholds for the sizes of gene ontology (GO) terms

This data set specifies the boundaries between GO terms containing medium and high numbers of genes. The GO term category (molecular function, biological process, cellular component) is specified and whether decreasing or increasing genes were selected. The same boundaries, overlaid with the sizes of individual GO terms, are presented in Supplementary Figure S10.

Supplementary Data Set S12 – Statistics for decreasing GO terms

Individual Excel sheets contain the p-values was two types of statistical tests, which were applied to GO terms within GO term categories and of similar sizes (see "Analysis of gene expression over pseudotime" in Methods). "kw_p_value" represents the result for the Kruskall-Wallis test. For the values of the "kwAllPairsNemenyiTest" test, each adjusted p-value is reported between each pair of compared GO terms (Nemenyi 1963; Pohlert 2023).

Supplementary Data Set S13 – Statistics for increasing GO terms

This has the same structure as data set S12, but contains information about increasing GO terms.

Supplementary Data Set S14 – Link list for the gene regulatory network (GRN)

This data set contains the list of edges and related edge weights for the final GRN. The columns include:

- 'weight' for the relative weight of the edge, as specified by the DynGENIE3 package (Huynh-Thu and Geurts 2018).
- 'regulatory gene' for the source node of the edge. The values will be one of the 46 possible transcription factors.
- 'target gene' for the target node of the edge.

Supplementary Data Set S15 – Variant calling file

This file is a standard variant calling format (VCF v4.2) file, containing information about the haplotype of each sample, for the final filtered variants.

Supplementary Data Set S16 – Statistics for linear models based on variant subgroups

This Excel sheet contains a summary of the linear model trained per gene. The response variable was the log-transformed gene expression, and the covariates included a constant term (to account for mean gene expression) and the variant subgroups (as defined in Supplementary Figure S14). This Excel sheet contains values produced by ‘summary.lm’ in R, namely:

- the estimate and standard error for the subgroup coefficient, and
- the t-value, two-sided p-value and adjusted p-value (using the Benjamini and Hochberg method to control the false discovery rate) for the associated t-test (Benjamini and Hochberg 1995).

Supplementary Figures

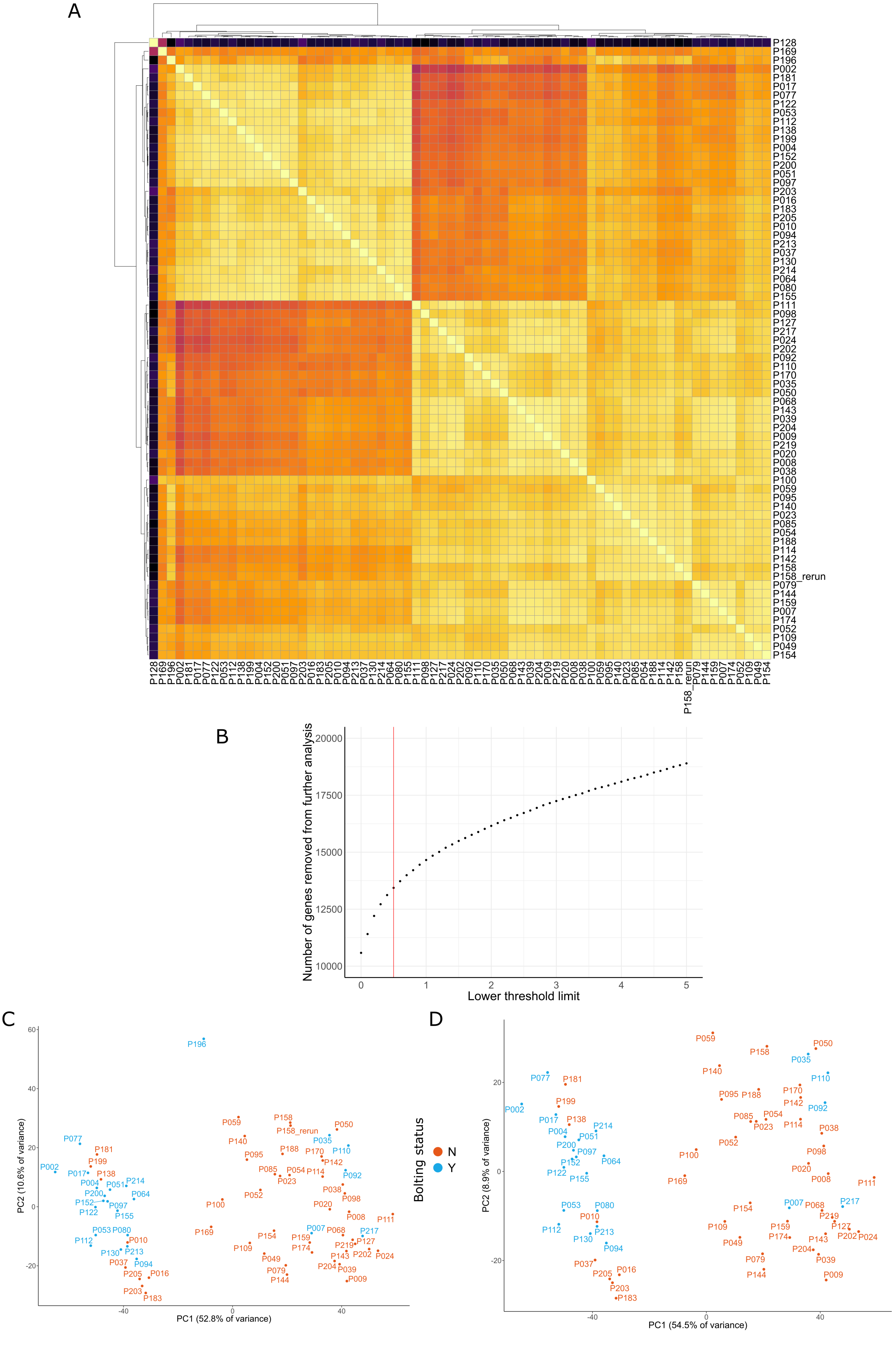

Supplementary Figure S1: **Plots related to filtering of the RNA-seq dataset. Supports Figure 1. A**: Pearson correlation of each sample based on the entire unfiltered transcriptome (after log transformation). P128 is a clear outlier, with low correlation with every other sample. Rows and columns of the heatmap have been reordered according to hierarchical clustering. **B**: The number of genes which would be filtered out based on different low expression thresholds. **C:** PCA of samples based on log-transformed TPM values, after the initial removal of sample P128 and low-expressed genes. One further sample was removed (P196), since it was far away from all other samples. Additionally, P158 and P158_rerun are adjacent in the reduced dimensionality space, justifying the replacement of P158 with P158_rerun. **D:** PCA of samples after final filtering, with the same conditions as **C**. (PC, principal component; N, not bolting; Y, bolting)

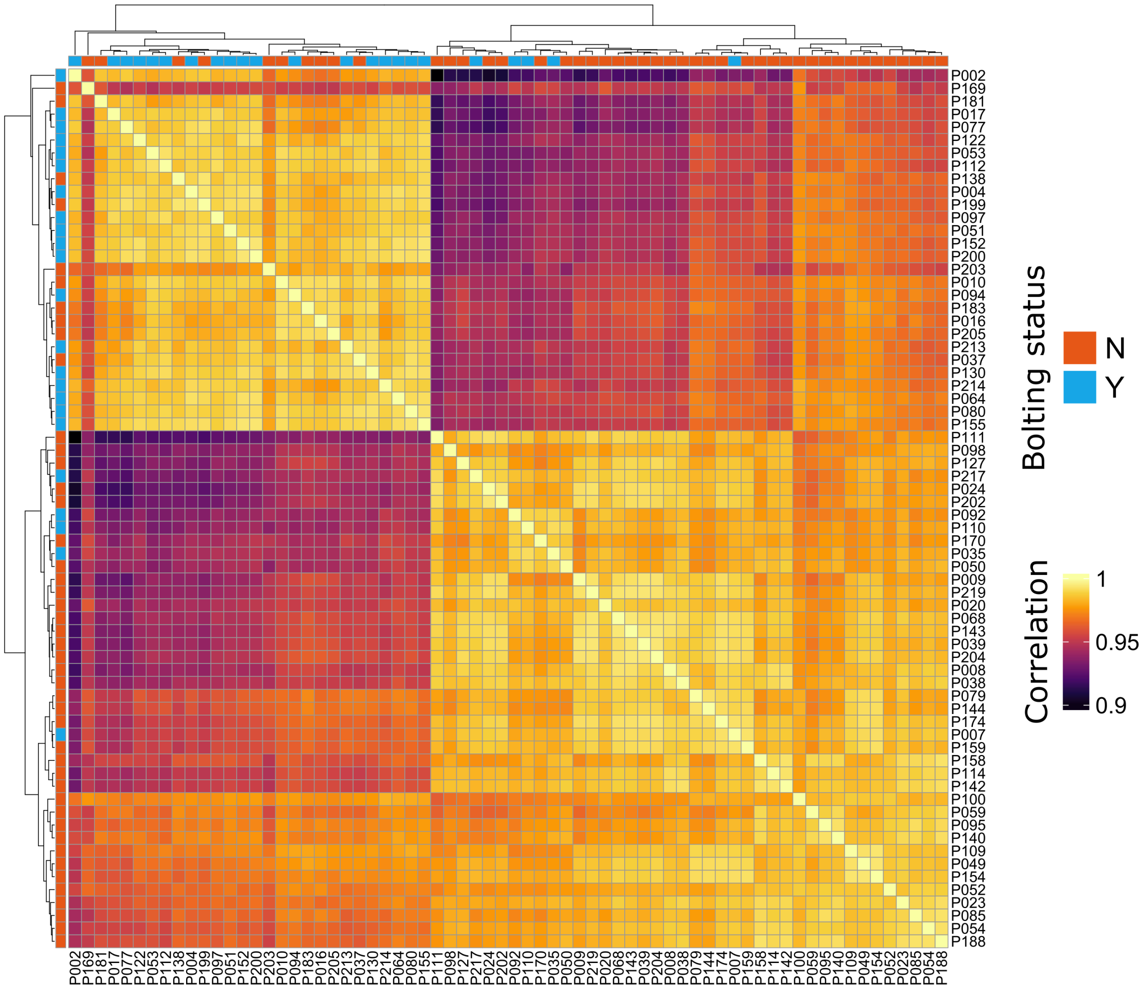

Supplementary Figure S2: **Clustering of samples, based on correlation of log-scaled TPMs. Supports figure 1.** All comparisons between samples show a Pearson correlation coefficient > 0.89. Hierarchical clustering (with the ‘complete’ linkage method) shows that the samples can be separated into two clusters. One cluster contains mostly non-bolted plants and the other contains mostly bolted plants. (N, not bolting; Y, bolting)

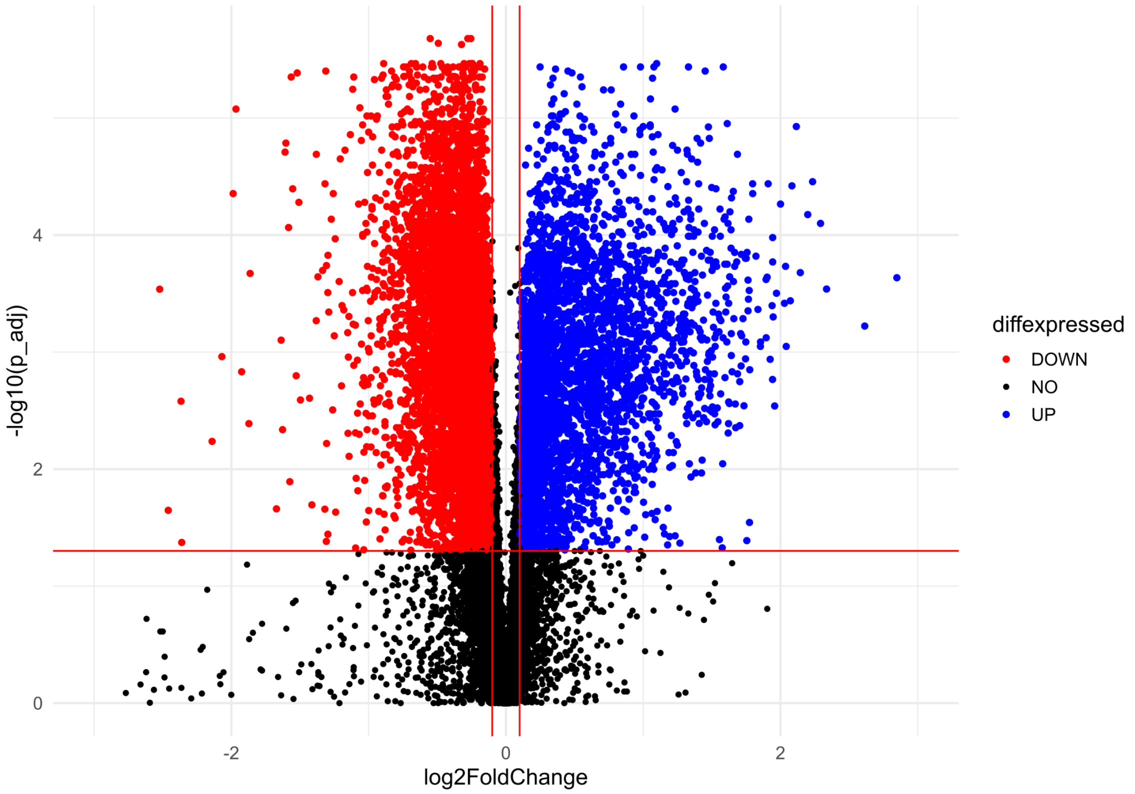

Supplementary Figure S3: **Volcano plot to show differentially expressed genes, based on the bolting status phenotype. Supports Figure 1.** We identified genes that: (i) had an adjusted p value of at least 0.05 and (ii) showed a log fold change of more than 0.1 or less than -0.1. (Red lines indicate the boundaries for these decisions.) Out of the 19 283 genes used in this analysis, we identified 6 967 down-regulated genes and 3 734 up-regulated genes (see Supplementary Data Set S3).

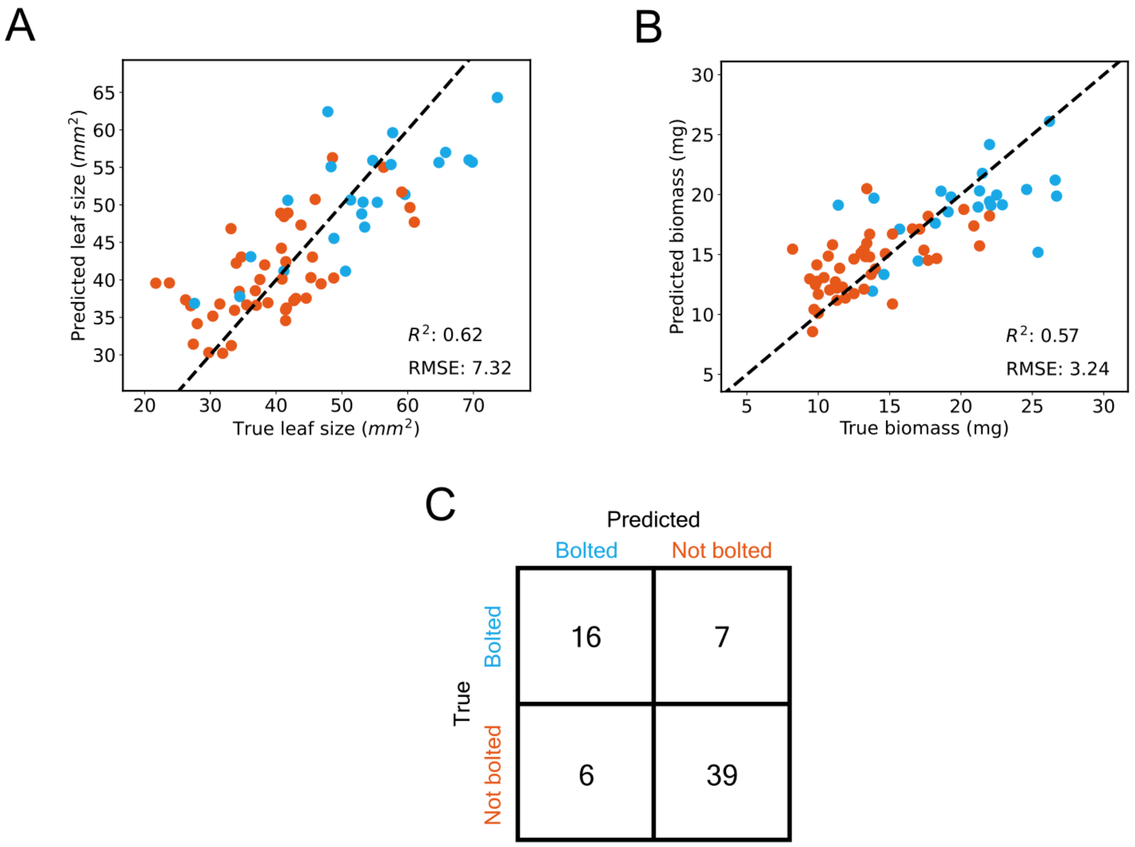

Supplementary Figure S4: **Performance of elastic net and logistic regression models trained on all genes. Supports Figure 2. A, B:** The same as Figures 2A, B from the main text, except these models included all genes as potential predictors. **C:** The same as Figure 2D from the main text, except these models included all genes as potential predictors. The accuracy across all logistic regression models is 80.9%. (*n* = 68 total sample size; RMSE, root mean squared error)

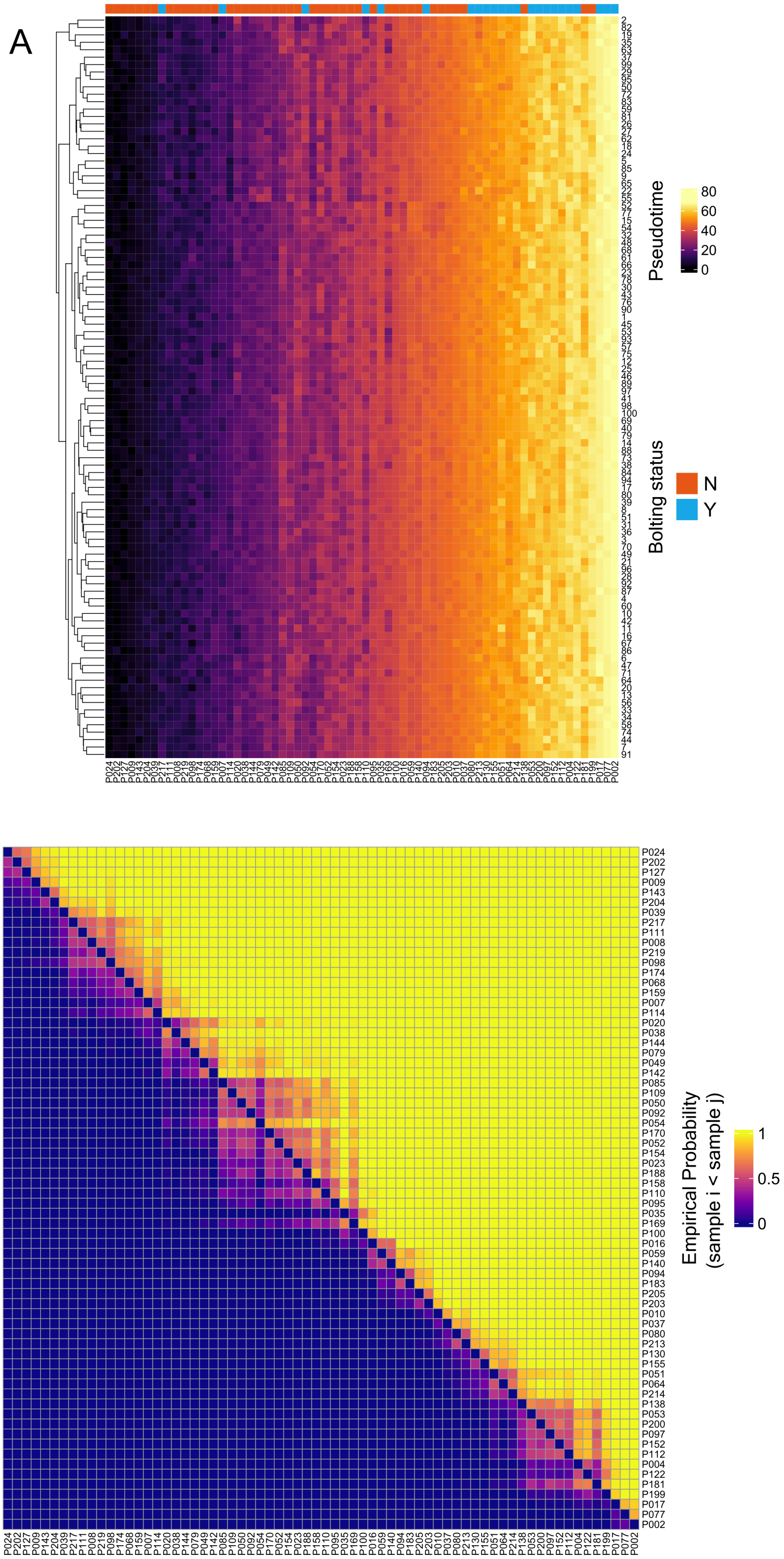
A
Pseudotime
80
60
40
20
0
Bolting status
N
Y
B
Empirical Probability
(sample i < sample j)
1
0.5
0

Supplementary Figure S5: **Summary of the bootstrapping process used to infer pseudotime. Supports Figure 3. A**: Our pseudotime inference method produces consistent results across different groups of input genes for most samples. Values correspond to predicted pseudotime for each sample (columns), as predicted within each partition of the genes (rows). Columns are ordered by the final consensus pseudotime. **B**: There are samples which show some uncertainty in pseudotime prediction - as shown by the clusters of squares around the diagonal. Values correspond to the empirical probability (proportional to the number of observed events in bootstrapping) that the sample of that row is earlier than the sample of that column. Rows (up-to-down) and columns (left-to-right) are ordered by consensus pseudotime. (N, not bolting; Y, bolting)

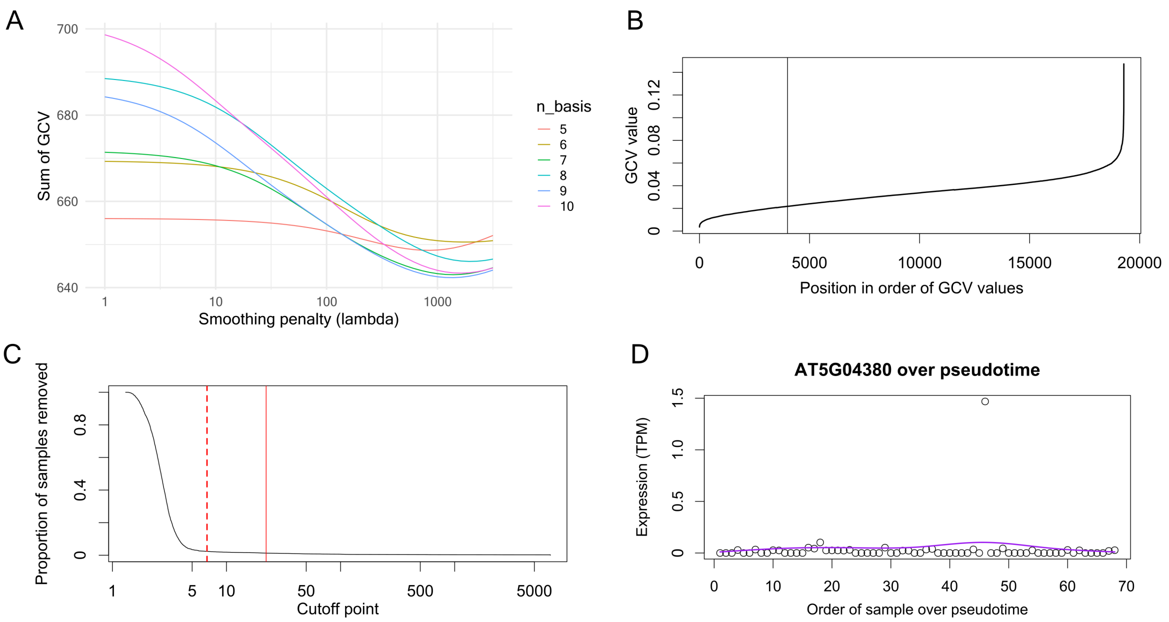

Supplementary Figure S6: **Selections of hyperparameters and filtering of genes after B-spline smoothing. Supports Figure 3. A**: Selection of optimal parameters for B-spline smoothing. Values correspond to the sum of GCV (generalized cross-validation) across all genes, after B-spline fitting with the fixed number of basis functions (n_basis) and the value of the second derivative smoothing penalty (lambda). *Note:* for reference, the optimal parameters chosen were n_basis = 9 and lambda = 1390 (3 s.f.). **B**: Selection of threshold based on GCV value of each gene. All genes which passed initial filtering are displayed. Genes with high GCV are assumed to not vary over pseudotime and they are therefore removed before further analysis. We selected the 4000 genes with lowest GCV (indicated by the vertical line). **C**: Selection of threshold for interquartile range. Some genes had low GCV, despite displaying poor behavior over pseudotime (for example, only 1 or 2 samples with high TPM). These were removed by only keeping genes with a low range to interquartile range ratio. Genes with a ratio higher than 6.75 were removed (indicated by the dashed red line). The solid red line corresponds to AT5G04380. **D**: An example of a gene (AT5G04380) removed by the previous filtering. This gene had a ratio of 22.3 (3 s.f.). The purple line is the B-spline smoothed curve and the points represent TPM values in each sample. (*n* = 68 total sample size)

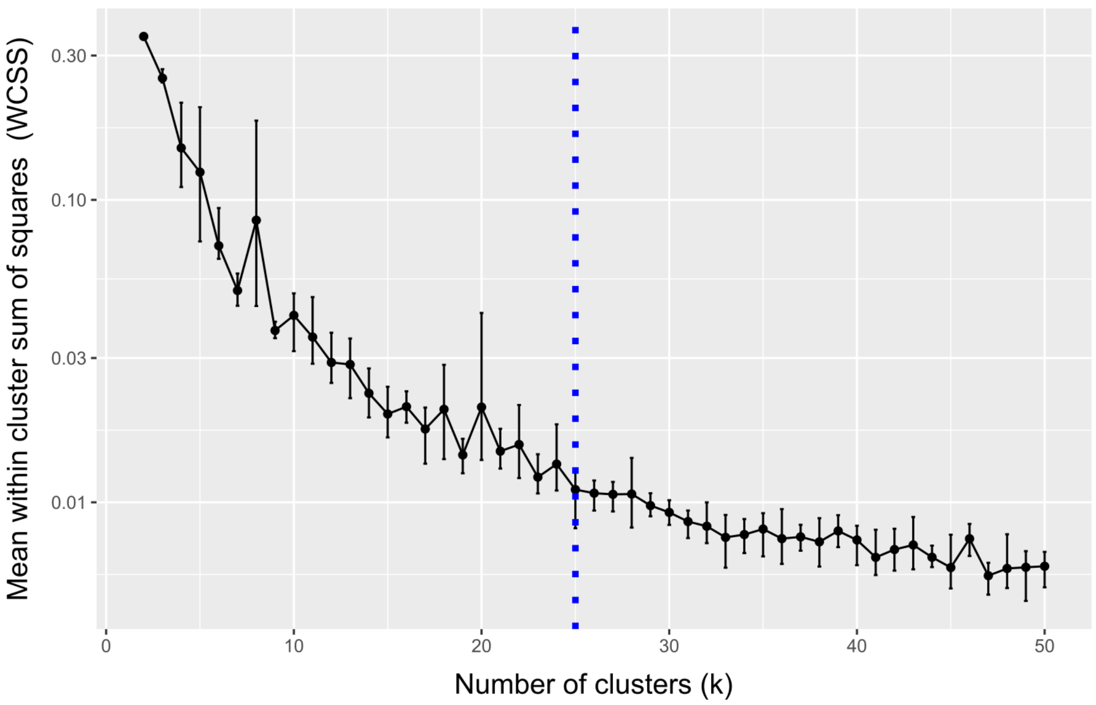

Supplementary Figure S7: **Selection of number of clusters for Supplemental Figures 8 and 9. Supports Figure 3.** The k-Shape clustering process was repeated for 5 fixed seeds, per predefined number of clusters (k). The points represent mean WCSS calculated across the 5 repeats per k (see Methods for details). Error bars represent the range from minimum to maximum WCSS per k. The blue dashed line represents the number of clusters chosen (k = 25) for Supplemental Figures 8 and 9, since the decrease in WCSS values after k = 25 is more gradual than before. (WCSS, within cluster sum of squares)

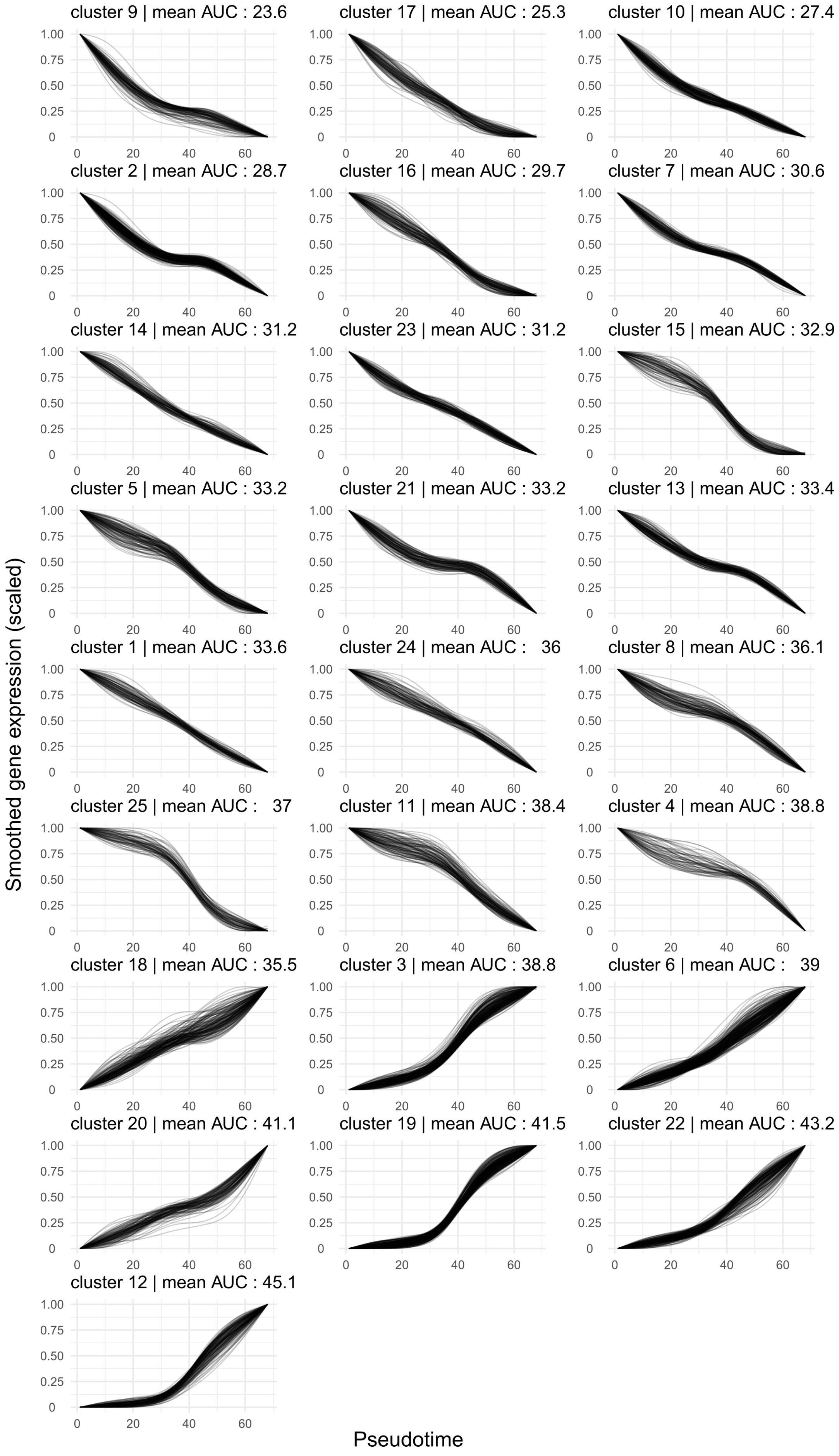

cluster 9 | mean AUC : 23.6
cluster 17 | mean AUC : 25.3
cluster 10 | mean AUC : 27.4
cluster 2 | mean AUC : 28.7
cluster 16 | mean AUC : 29.7
cluster 7 | mean AUC : 30.6
cluster 14 | mean AUC : 31.2
cluster 23 | mean AUC : 31.2
cluster 15 | mean AUC : 32.9
cluster 5 | mean AUC : 33.2
cluster 21 | mean AUC : 33.2
cluster 13 | mean AUC : 33.4
cluster 1 | mean AUC : 33.6
cluster 24 | mean AUC : 36
cluster 8 | mean AUC : 36.1
cluster 25 | mean AUC : 37
cluster 11 | mean AUC : 38.4
cluster 4 | mean AUC : 38.8
cluster 18 | mean AUC : 35.5
cluster 3 | mean AUC : 38.8
cluster 6 | mean AUC : 39
cluster 20 | mean AUC : 41.1
cluster 19 | mean AUC : 41.5
cluster 22 | mean AUC : 43.2
cluster 12 | mean AUC : 45.1
Smoothed gene expression (scaled)
Pseudotime

Supplementary Figure S8: **Overall visualisation of gene expression over pseudotime, separated into 25 clusters. Supports Figure 3.** After filtering for monotonic genes, each gene was assigned into one of 25 clusters. Gene expression was normalized between 0 and 1 for the AUC process. These clusters have been ordered to show decreasing clusters first (from lowest to highest mean AUC) then increasing clusters second. The mean AUC ranges from 23.6 to 38.8 for decreasing clusters and 35.5 to 45.1 for increasing clusters. (AUC, area under the curve)

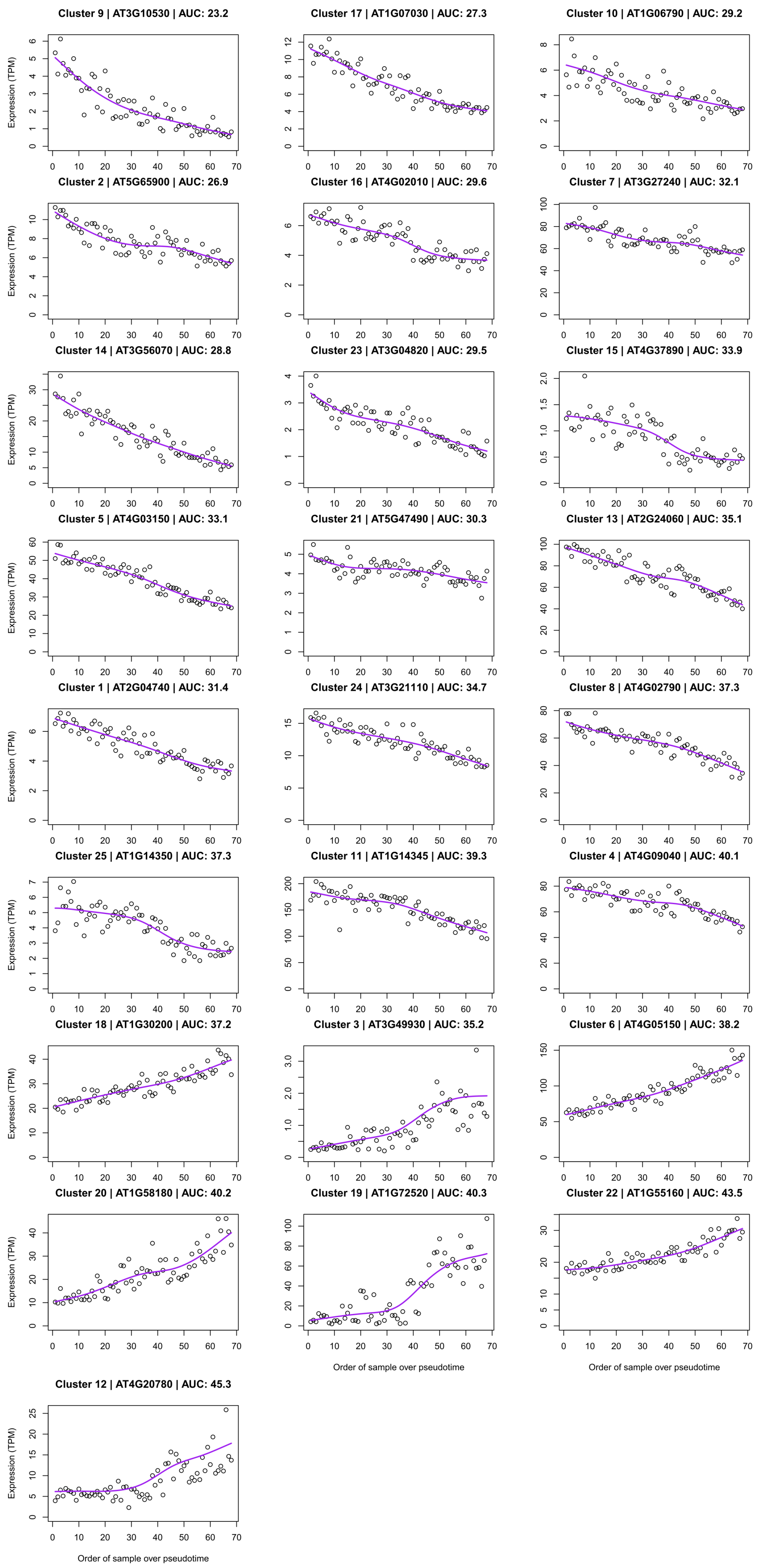

Cluster 9 | AT3G10530 | AUC: 23.2
Cluster 17 | AT1G07030 | AUC: 27.3
Cluster 10 | AT1G06790 | AUC: 29.2
Cluster 2 | AT5G65900 | AUC: 26.9
Cluster 16 | AT4G02010 | AUC: 29.6
Cluster 7 | AT3G27240 | AUC: 32.1
Cluster 14 | AT3G56070 | AUC: 28.8
Cluster 23 | AT3G04820 | AUC: 29.5
Cluster 15 | AT4G37890 | AUC: 33.9
Cluster 5 | AT4G03150 | AUC: 33.1
Cluster 21 | AT5G47490 | AUC: 30.3
Cluster 13 | AT2G24060 | AUC: 35.1
Cluster 1 | AT2G04740 | AUC: 31.4
Cluster 24 | AT3G21110 | AUC: 34.7
Cluster 8 | AT4G02790 | AUC: 37.3
Cluster 25 | AT1G14350 | AUC: 37.3
Cluster 11 | AT1G14345 | AUC: 39.3
Cluster 4 | AT4G09040 | AUC: 40.1
Cluster 18 | AT1G30200 | AUC: 37.2
Cluster 3 | AT3G49930 | AUC: 35.2
Cluster 6 | AT4G05150 | AUC: 38.2
Cluster 20 | AT1G58180 | AUC: 40.2
Cluster 19 | AT1G72520 | AUC: 40.3
Cluster 22 | AT1G55160 | AUC: 43.5
Cluster 12 | AT4G20780 | AUC: 45.3
Expression (TPM)
Order of sample over pseudotime

Supplementary Figure S9: **Examples of genes from each cluster. Supports Figure 3.** A random gene from within each cluster (as shown by the TAIR ID). For the majority of genes, the residuals between the fit and the real data are consistent across pseudotime. The order of the clusters matches Supplementary Figure S8. Black circles represent unnormalised TPM values. The purple line shows the B-spline fit before rescaling to have a minimum of 0 and maximum of 1. The AUC value was calculated on the rescaled B-spline fit. (AUC, area under the curve)

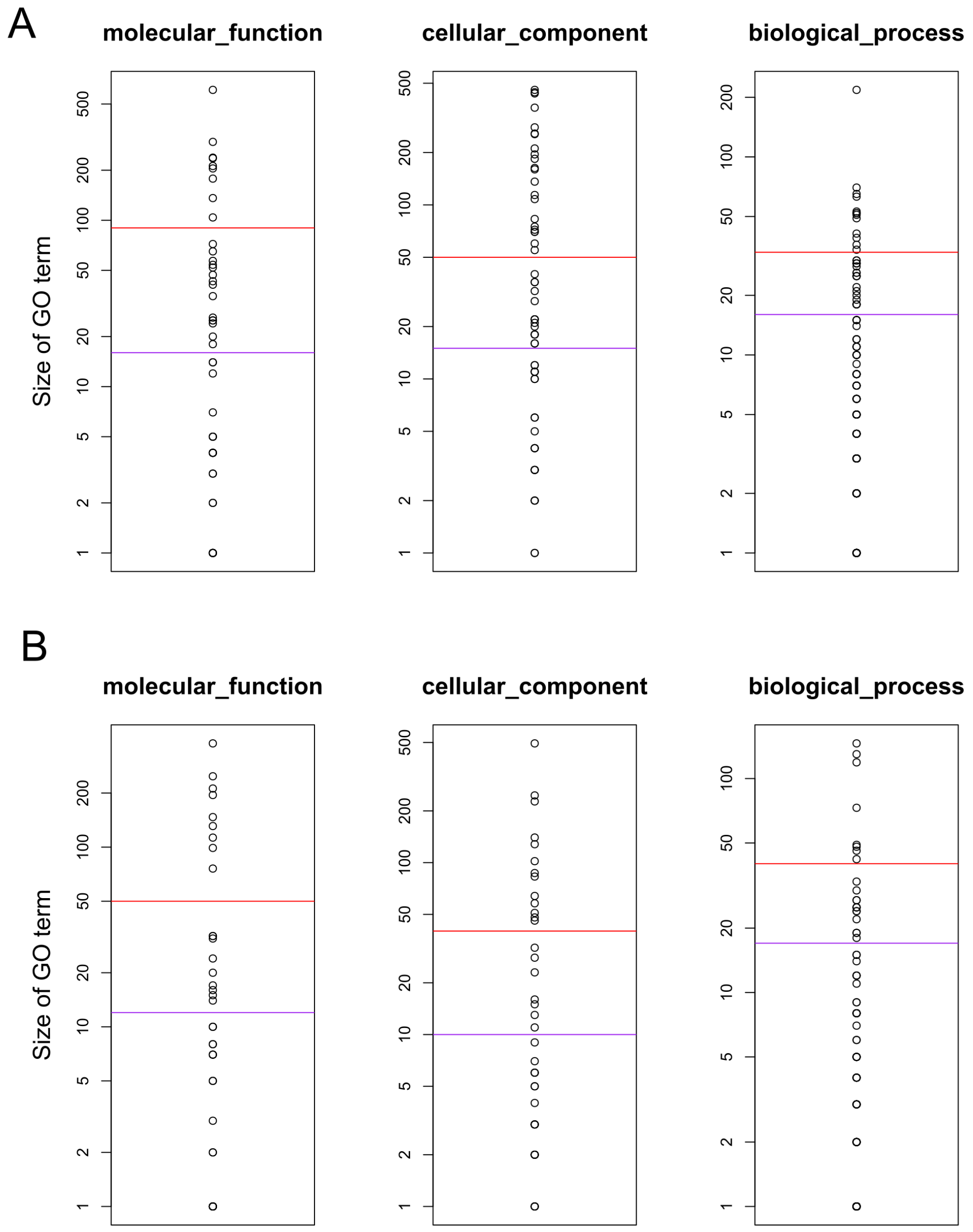

Supplementary Figure S10: **Selection of GO term sizes. Supports Figure 3.** In order to compare ‘area under the curve’ values between groups of GO terms, we first needed to select GO terms of approximately the same size (in terms of the genes which passed all previous filtering steps). Thus, we manually selected thresholds for ‘large’ (depending on condition, red line) and ‘medium’ (depending on condition, purple line) GO terms (Supplementary Data Set S11). Also, these values needed to be chosen independently for decreasing and increasing genes. **A**: Thresholds chosen for decreasing genes, per GO term. 2018 genes were classified as ‘decreasing’. **B**: Thresholds chosen for increasing genes, per GO term. 1352 genes were classified as ‘increasing’. (GO, gene ontology)

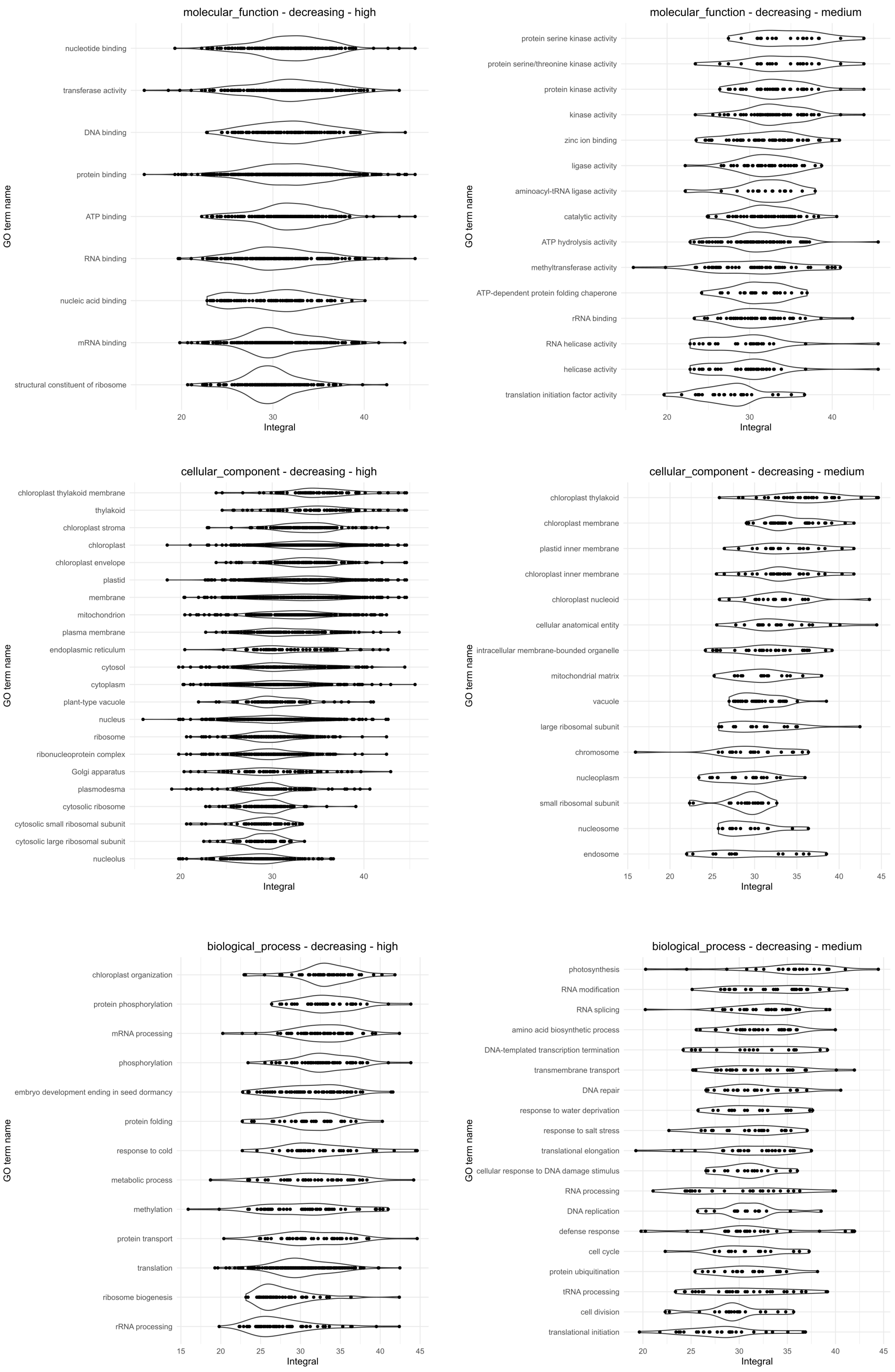

Supplementary Figure S11: **Area under the curve values for GO terms. Filtered to only include genes which decrease over pseudotime. Supports Figure 3.** Each violin plot is

titled by the category of GO term (molecular function, cellular component, or biological process) and the relative size of the GO term (high or medium; see Supplementary Figure S10). 2018 genes were classified as ‘decreasing’. *Note:* Kruskal-Wallis tests and the related post-hoc tests were performed. See Supplementary Data S12 for details. (GO, gene ontology)

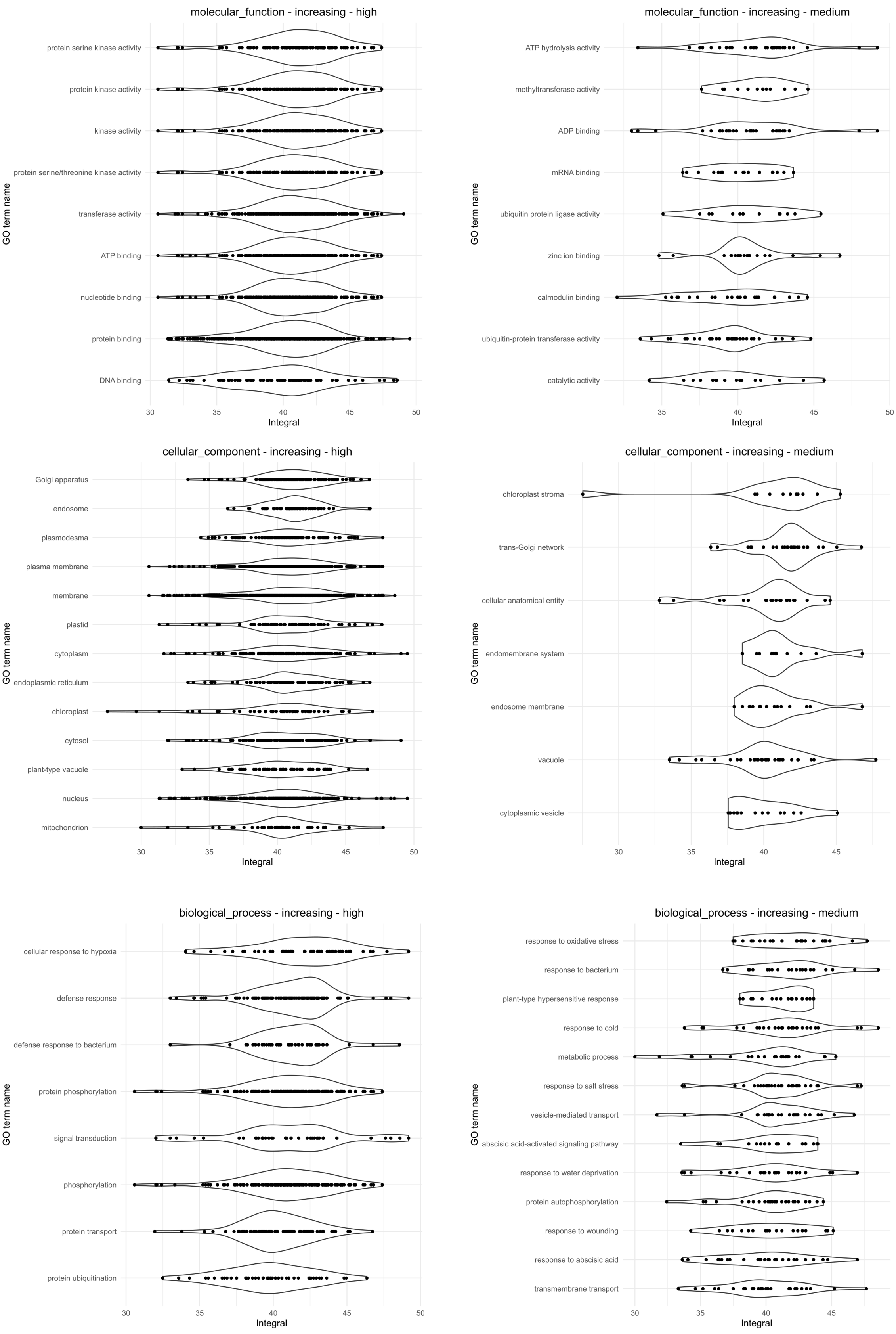

Supplementary Figure S12: **Area under the curve values for GO terms. Filtered to only include genes which increase over pseudotime. Supports Figure 3.** Identical structure to Supplementary Figure S11, except these are for increasing genes. 1352 genes were classified as ‘increasing’. *Note:* most of the Kruskal-Wallis tests and all of the post-hoc tests were not

significant (adjusted p value > 0.01) in each of these cases. See Supplementary Data Set S13 for details. (GO, gene ontology)

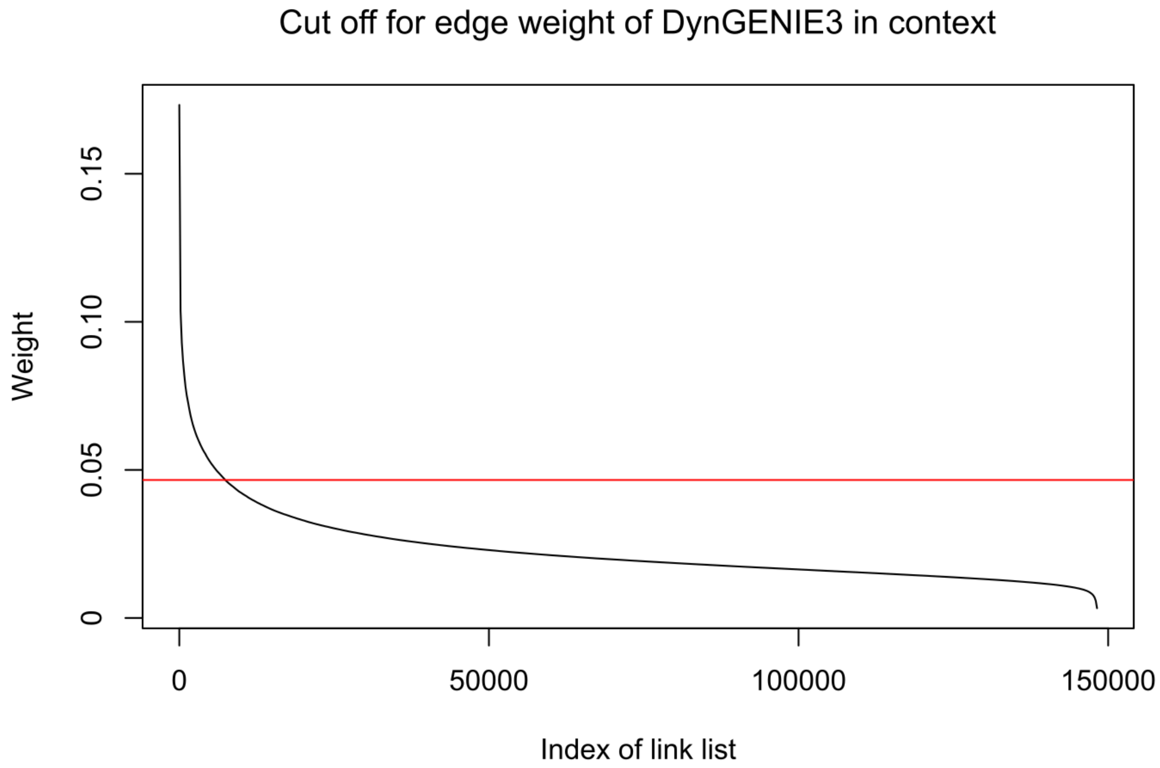

Supplementary Figure S13: **Edge weights of predicted gene-gene interactions by DynGENIE3. Supports Figure 4.** The ‘index of the link list’ is defined by decreasing edge weight. The top 5% of edges by weight were kept. The horizontal red line indicates the weight threshold; only edges with weight greater than this were included in the final network. *Note:* these weights are only meaningful in a relative sense and have no other statistical significance.

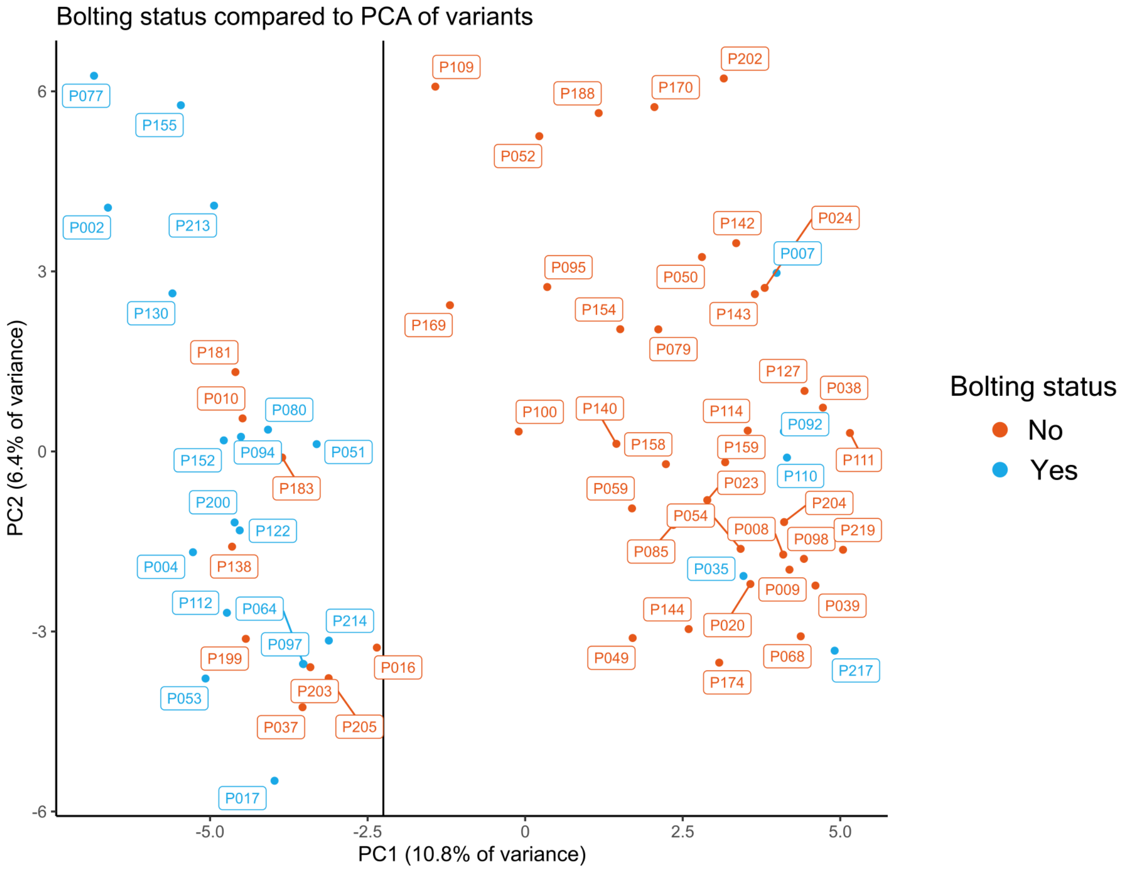

Supplementary Figure S14: **Variant-based PCA. Supports Figure 5.** PCA plot of individual plants based on variants. Before PCA was performed, the homozygous reference allele was assigned 0; the heterozygous allele was assigned 1; and the homozygous alternate allele was assigned 2. The vertical line follows PC1 = -2.25. Samples to the left of the line were assigned to subgroup 1 for the linear model analysis and contained mostly bolting samples (see Methods and 'A small number of variants are associated with pseudotime'), and the other samples were assigned to subgroup 2 and contained mostly non-bolting samples. (PC, principal component)

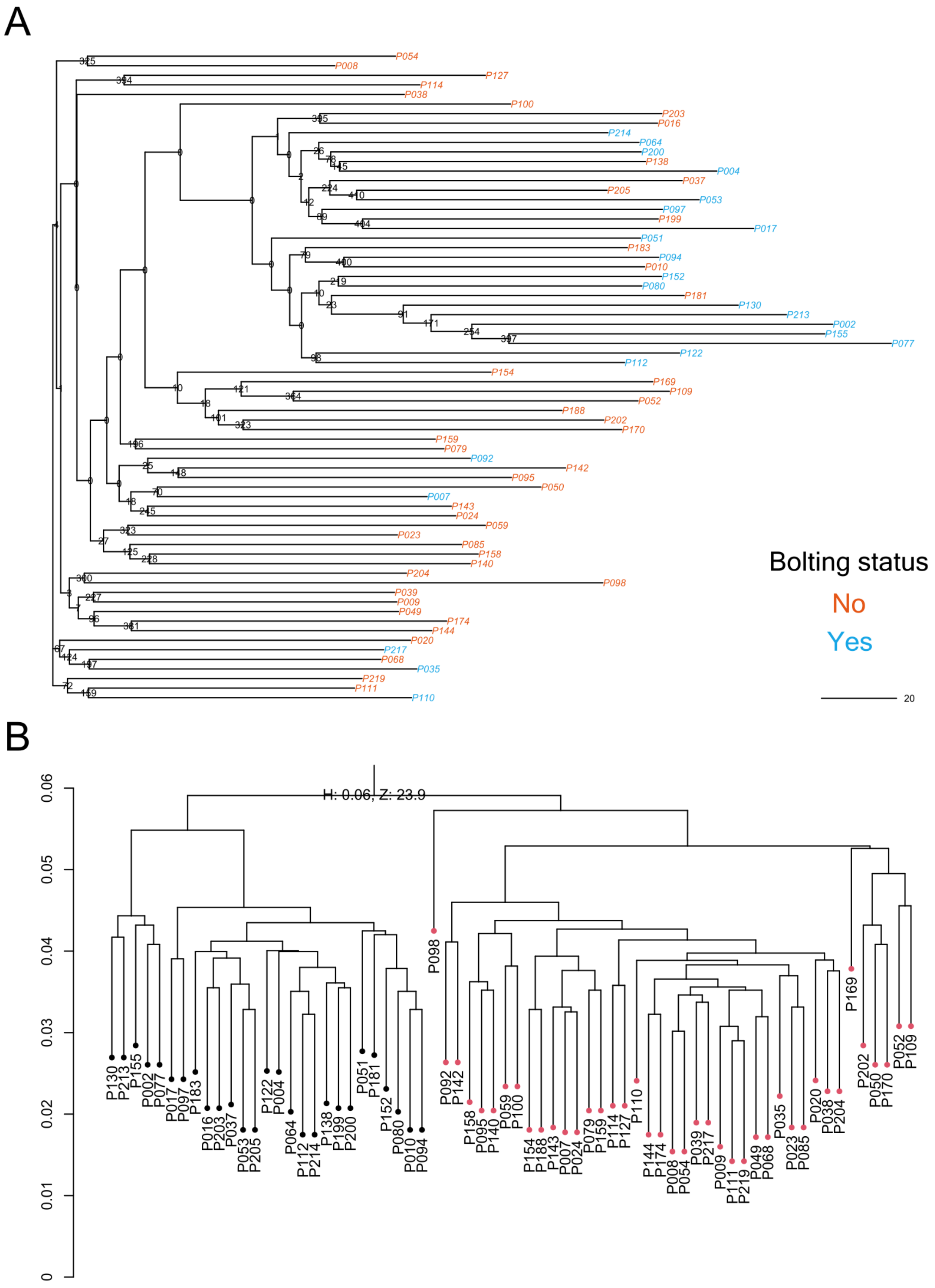

Supplementary Figure S15: **Two approaches of variant-based tree-building. Supports Figure 5. A:** Neighbor-joining tree on individual plants, followed by bootstrapping with 1000 repeats (Saitou and Nei 1987). Branch lengths (and the corresponding scale bar) represent the pairwise distance between samples – i.e. the number of variants where they differed. The labels on nodes represent how often that branch was observed when repeating the neighbor-joining method. **B:** An alternative approach to population structure analysis, using Identity-by-state analysis, from the SNPRelate package (Zheng et al. 2012). This differs to **A** since the method uses the 'average' metric during hierarchical clustering and performs a permutation test to find the number of distinct clusters. The Z-score on the top branch was 23.9, greater than the Z threshold of 15, indicating that two subgroups were present. These separate

subgroups are indicated by the top branch and the black and red dots next to each sample name.

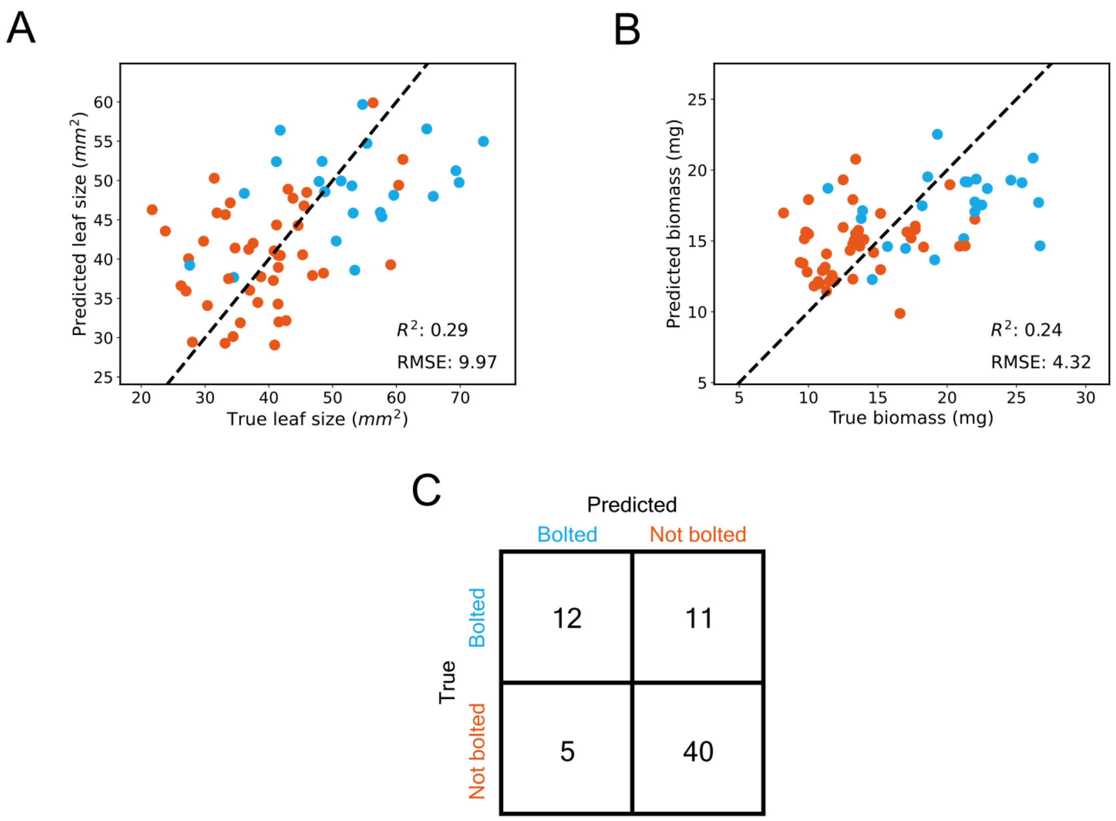

Supplementary Figure S16: **Elastic net and logistic regression models trained on variants. Supports Figures 2 and 5. A, B:** The same as Figures 2A, B from the main text, except these models use variants as potential predictors. **C:** The same as Figure 2D from the main text, except these models use variants as potential predictors. The accuracy across all logistic regression models is 76.5%. *Note:* in all models, a number was assigned to each allele to be used as input data. Specifically, the homozygous reference allele was assigned 0; the heterozygous allele was assigned 1; and the homozygous alternate allele was assigned 2. (*n* = 68 total sample size; RMSE, root mean squared error)

Supplementary references

Benjamini Y and Hochberg Y. Controlling the False Discovery Rate: A Practical and Powerful Approach to Multiple Testing. J R Stat Soc Series B Stat Methodol. 1995:**57**(1):289–300.

Huynh-Thu VA and Geurts P. dynGENIE3: dynamical GENIE3 for the inference of gene networks from time series expression data. Sci Rep. 2018:**8**(1):3384. https://doi.org/10.1038/s41598-018-21715-0

Kolberg L, Raudvere U, Kuzmin I, Vilo J, and Peterson H. gprofiler2 -- an R package for gene list functional enrichment analysis and namespace conversion toolset g:Profiler. F1000Res. 2020:**9**. https://doi.org/10.12688/f1000research.24956.2

Nemenyi PB. Distribution-free Multiple Comparisons. 1963.

Pohlert T. PMCMRplus. 2023. https://CRAN.R-project.org/package=PMCMRplus

Saitou N and Nei M. The neighbor-joining method: a new method for reconstructing phylogenetic trees. Mol Biol Evol. 1987:**4**(4):406–425. https://doi.org/10.1093/oxfordjournals.molbev.a040454

Woo HR, Koo HJ, Kim J, Jeong H, Yang JO, Lee IH, Jun JH, Choi SH, Park SJ, Kang B, et al. Programming of Plant Leaf Senescence with Temporal and Inter-Organellar Coordination of Transcriptome in Arabidopsis. Plant Physiol. 2016:**171**(1):452–467. https://doi.org/10.1104/pp.15.01929

Zheng X, Levine D, Shen J, Gogarten SM, Laurie C, and Weir BS. A high-performance computing toolset for relatedness and principal component analysis of SNP data. Bioinformatics. 2012:**28**(24):3326–3328. https://doi.org/10.1093/bioinformatics/bts606
