# Supplementary material for: Single-plant-omics reveals the cascade of transcriptional changes during the vegetative-to-reproductive transition
Source: Plant Cell. 2024 Aug 9;36(10):4594–606. doi: 10.1093/plcell/koae226 (PMC11449079; doi:10.1093/plcell/koae226)
Supplement: koae226_Supplementary_Data [file tpc.00965.2023-s22.pdf › tpc.00965.2023-s22.pdf]

THE PLANT CELL

# Single-plant-omics reveals the cascade of transcriptional changes during the vegetative-to-reproductive transition

Ethan Redmond, James Ronald, Seth Davis, and Daphne Ezer

**Review Timeline:**

| | |
|---|---|
| Submission Date: | 13-Oct-2023 |
| Editorial Decision: | 15-Jan-2024 |
| Revision Received: | 12-Apr-2024 |
| Editorial Decision: | 13-May-2024 |
| Revision Received: | 11-Jun-2024 |
| Editorial Decision: | 20-Jun-2024 |
| Revision Received: | 26-Jun-2024 |
| Accepted: | 01-Jul-2024 |

Dr. Daphne Ezer
University of York
Biology
Heslington YO310RT
United Kingdom

Dear Dr. Ezer:

First, we sincerely apologize for the length of time that your manuscript was under consideration. A reviewer failed to return their comments, even after we had waited to receive these.

We have now received reviews of your manuscript entitled "Single-plant-omics reveals the cascade of transcriptional changes during the vegetative-to-reproductive transition." Thank you for submitting your best work to The Plant Cell. The editorial board agrees that the work you describe is substantive, falls within the scope of the journal, and may become acceptable for publication, pending revision and potential re-review.

In addition to addressing each point that the reviewers have made, we ask you to pay special attention to the following points in preparing your revision:

There is an overriding concern that many of the results obtained may be skewed, because there may in fact be two genetically distinct subpopulations of plants incorporated in this study, rather than one genetically nearly homogeneous population. Therefore, it is important to provide convincing evidence that this is not the case. Please also special care to address all additional concerns about the stated assumptions and methodology.

Please contact us if there are ambiguous comments or if you wish to discuss the revision.

Given the nature of the comments, we are offering you 60 days from when we have issued this decision to complete the revision. If you need an extension, we encourage you to contact us at any point before the 60 days have passed.

When you are ready to submit the revised version, please upload a highlighted copy that indicates all changes made in response to the editor and reviewer recommendations. Include an itemized list of all changes made in response to each of the reviewer's suggestions in the "Response to Reviewers" section; please note that reviewers do not have access to your cover letter, nor was this decision letter shared with them.

Thank you for the privilege of reviewing your work. We look forward to receiving your revised manuscript.

Sincerely,

The Plant Cell Editorial Board

**Please note the following:**

**-The Plant Cell now requires authors to complete and submit an author revisions checklist upon submission of a revised manuscript. The aim of the checklist is to aid authors in preparing a high-quality manuscript, facilitate the review and assessment of revised manuscripts, and help to ensure that journal standards are maintained across the board. If your manuscript is accepted, the completed checklist will be published as supplemental material attached to the article online. Please download a copy of the checklist (pdf fillable form) at this link, for submission with your revised manuscript: https://tpc.msubmit.net/html/Author_Revisions_Checklist.pdf.**

**-Supplemental materials should be restricted to large datasets and tables, presentation of replicates, and validation of reagents, methods, or genotypes. Any data that are used to support the major claims must be in the main manuscript. Supplemental figure legends must indicate what figure in the main manuscript is supported by the supplemental data presented. Please justify how each of the supplemental figures meet the criteria.**

**-Sampling methods and nature of "biological replicates" should be described precisely (i.e. different plants, parts of plants, pooled tissue, independent pools of tissue, sampled at different times, etc), along with a clear description of and rationale for any statistical analyses conducted. The reader should know exactly what was sampled; what forms**

the basis of the calculation of any means and statistical parameters reported. This is also necessary to ensure that proper statistical analysis was conducted.

-Want to add this revision deadline to your calendar? Click below!

------------------------------------------------------------------------ Reviewer comments:

Reviewer #2 (Comments for the Author):

The authors present a study in which they use a single-plant -omics profiling strategy to unravel the timing of events during the vegetative-to-reproductive phase transition in Arabidopsis thaliana. The overall workflow of the study is very similar to the workflow followed in ref 9 (Cruz, De Meyer et al. (2020) Mol. Syst. Biol. 16, e9667), but focused on studying a particular developmental transition. The major innovative aspect of the study in my opinion is the use of a trajectory inference algorithm (as in single-cell data analysis) to order the individual plants along a pseudotime trajectory, which allows for inference of gene regulatory networks from the resulting pseudotime series data (in this case using DynGENIE3). For full disclosure: my group has similarly been using single-cell techniques such as trajectory and GRN inference on single-plant -omics data to study the vegetative-to-reproductive phase transition in Brassica napus, inspired by observations we made in a previous single-plant -omics study on field-grown rapeseed (De Meyer, Cruz et al. (2023) PLoS Comput Biol, https://doi.org/10.1371/journal.pcbi.1011161). We already presented results on this work at several seminars and conferences, but we are still about 3 months away from submitting the associated manuscript.

Perhaps not surprisingly given my background in single-plant -omics, I really like the concept of using subtle timing differences in a population of genetically nearly identical plants to study plant developmental processes. I'm also rather excited that the single-plant profiling concept, which I have been advocating for the past decade (Bhosale et al. (2013) Plant Cell, https://doi.org/10.1105/tpc.113.112268) is being picked up. I do however still have a number of comments on this manuscript.

Major comments

1) The PCA analysis in Supplemental Figure 6 (which I assume is based on all transcripts, although this is not clear from the methods or figure legends) shows two clear subgroups of plants. Similarly, the clusterings in Figure 1A, Supplemental Figures 1A and 2A suggest two subgroups, and in particular the SNP profiles in Figure 5B suggest that there may be two genetically different subgroups of plants in the dataset, with a substantial number of SNP profiles correlating strongly with gene expression profiles and the bolting status of the plants. If so, the single-plant character of the study may be compromised in the sense that the differences between subgroups may dominate/swamp the differences between individual plants in each subgroup, and many of the results obtained may have to be viewed as the result of a treatment vs control experiment with a lot of replicates, rather than a single-plant omics experiment on a genetically nearly homogeneous population. We previously encountered similar population structure issues in both the ref. 9 study and the aforementioned Bhosale et al. (2013) Plant Cell study, and corrected for these (or at least their linear effects) before performing downstream analyses. In the present case, the effects of potential genetic substructure may be of particular concern given the strong correlation this substructure (if confirmed) may have with the bolting phenotype and hence the progress of the vegetative-to-reproductive phase transition under study. In the worst case, the population of plants could essentially consist of a group of plants of genotype 1 in which the vegetative-to-reproductive phase transition has barely started and another group of plants of genotype 2 where the transition has been finalized, so essentially an early and a late developmental timepoint with little in between (although this would also depend on the within-group variance in developmental timing). This may in turn jeopardize the pseudotime trajectory inference performed and its interpretation (see also comment 3). Subgroup structure may also affect e.g. the elastic net performances for the quantitative leaf size and biomass traits in Figure 2 if the subgroups would have substantially different leaf size and biomass on average. In order to check for genetic substructure in the population, you could perform SNP-based tree building and SNP-based PCA analysis, and visualize SNPs associated with any population substructure uncovered along the chromosomes, as in ref. 9.
2) Figure 2 : why was bolting status not predicted from the transcriptome, next to leaf size and biomass ? This would be a classification rather than a regression problem but should be easy to do, and my guess is this classification model would actually perform much better even than the regression models shown for leaf size and biomass.
3) I appreciate the effort to build a trajectory inference algorithm tailored to the small size of the dataset at hand, but the approach followed has a number of drawbacks. First, the approach used assumes that all genes (or at least those that are DE in bolted versus non-bolted plants) are monotonically increasing or decreasing, which is not necessarily the case in developmental trajectories (e.g. when they exhibit different intermediate stages). Second, and perhaps more importantly, the pseudotimes inferred in the end are essentially a ranking of samples from 1 to 65. This means the samples are spaced equidistantly over pseudotime, while in reality some samples may cluster closer together in developmental time than others (which may especially be the case if there would be plant subgroups, cfr. comment 1).

This also has implications for downstream analyses such as the smoothing performed, the GRN inference with DynGENIE3 and in particular the discussion of changes in expression occurring earlier or later in the bolting process. Sentences like on line 174: '...pseudotime captures the continuous changes in gene expression of bolting-related processes, demonstrating that the transcriptional shift during bolting is not a binary process.' may not be accurate in this respect.
4) I really appreciate the effort to try and make GRNs by analyzing the pseudotime series, but I miss a bit the biological interpretation of the GRN shown in Figure 4. Does the order of TFs increasing/decreasing over pseudotime make sense given what is known on the vegetative-to-reproductive transition in A. thaliana (and more particularly the transition processes happening in leaves rather than SAMs) ?
5) Line 337-338 : Five samples were discarded as outliers based on the fact that they form a separate cluster when the clustering dendrogram in Supplemental Figure 1C is cut at some (arbitrary ?) threshold. Only one of them (P128) can be considered a real outlier, removal of the other plant samples is unnecessary as their expression profiles are highly correlated with plenty of other samples (see Supplemental Figure 1A).
6) line 347: it is unclear where the CPM counts come from, and whether they were properly normalized across samples (as done e.g. in the edgeR or DESeq2 protocols). Salmon does not produce CPMs to my knowledge. The Salmon TPMs are likely also not the best possible expression measures to use for e.g. training elastic nets, even after z-scoring, see e.g. https://doi.org/10.1186/s12967-021-02936-w . Am I right in assuming that these TPM values were not log-transformed for use in e.g. the elastic nets?

Minor comments

7) Line 79-80: 'large population' : please give a number of plants
8) Some statements in the text are not adequately supported by statistics :
a. Line 87-88: '...plants with the same bolting status had more closely correlated transcriptomes than plants with differing bolting statuses.'
b. Line 193-194: 'Interestingly, the term plasmodesma decreases significantly earlier than chloroplast,'
9) Table S2 : it is unclear whether p-values have been adjusted for multiple testing or not (I assume yes)
10) line 139-140 and first section of supplemental material: it would be interesting to compare the predictors found and the processes they are involved in (in particular for leaf area) with those found in rapeseed in a similar setup at a comparable developmental time point (vegetative-to-reproductive phase transition), see De Meyer, Cruz et al. (2023) PLoS Comput Biol, https://doi.org/10.1371/journal.pcbi.1011161 .
11) Supplemental Figure 7 legend : 'Genes with a ratio lower than 6.75' : lower should be higher.
12) Supplemental Figure 8 legend : 'we first needed to select GO terms of approximately the same size' : why is this ?

Steven Maere

Reviewer #3 (Comments for the Author):

Review: Single-plant-omics reveals the cascade of transcriptional changes during the vegetative-to-reproductive transition

This article uses RNA-seq on leaves from bolted and unbolted plants to identify differentially expressed genes and those associated with leaf size and biomass. A pseudo-time ordering of plants based on sum of standardized gene expressions is developed and used to estimate and compare time trends in gene expressions associated with the bolting-related processes.

The paper is well-written and nicely organized. There is a wealth of analytical results provided and the authors are to be commended on making the data and code publicly available for reproducibility purposes. The code is well documented and includes instructions for setting up the environment used to conduct the analysis to facilitate reproduction. Below are some comments that need to be addressed regarding the methodology and analytical results related to the smoothness and monotonicity assumptions, modeling approaches used to achieve smooth and monotonic time trends, statistically significant clusterings, and bootstrapping. There are also minor comments on the writing and structure.

Methodological and analytical
1. Page 10 line number 164-169: It is hard to understand why having a majority of genes being differentially expressed during bolting would be able to serve as the basis for the assumption that the change in expression levels is monotone over pseudotime. Of course, assumptions are necessary, especially when sample sizes are small, but this justification for the assumption seems weak. While fitting a monotone trend is probably the best one could hope to accomplish given the limited data, there could be many possible non-monotone trajectories that could lead to changes in expression associated with bolting. Are there any longitudinal studies that can be referenced to further justify this assumption? Are there any mechanistic explanations that could also justify this assumption? Since this assumption is

the basis for much of the methodology and results presented, it is worth a closer look.

2. Results Section 3 page 10 mentions that the ordering to generate the pseudo time series of gene expression relies on a monotonicity assumption, and the Discussion on page 14 says the ordering relies on a smoothness assumption. These are very different types of assumptions. A function can be smooth but not monotone and vice versa. If both are needed, both need to be clearly stated.

3. Page 19 line number 395-408: The B-spline basis will not enforce the monotonicity assumption described on page 10 and will lead to non-monotone (but smooth) gene expression trajectories, some of which can be seen in Figure 4B-4C. Monotone spline bases, like I-splines (Ramsay, 1988), should be used to achieve both smoothness and monotonicity. This will align your analyses with your assumptions and avoid the need to filter out non-monotonic gene expression trajectories (page 20 line number 410-420).

4. Supplementary Figure 1B-1C: How was the low expression threshold determined? How was the height of 11,500 determined in order to remove the further samples? There are methods that can be used to determine the statistical significance of a particular clustering given a dendrogram (Kimes et al., 2017). Please consider using this for identifying meaningful clusterings.

5. Isn't it possible that a gene with a quick increase in pseudotime can have a similar AUC to a gene exhibiting a slow decrease in pseudotime? If so, then genes should be grouped by increasing vs. decreasing expression levels and analyzed separately.
According to Page 19-20 line number 382-383, down-regulated genes were transformed (1-x), which should make the trends all going up over pseudotime, right? Wouldn't that change the interpretation of the AUC calculation, since a slow decrease (which should have large AUC) would be transformed to a quick increase (which will have a small AUC)? Please clarify.

6. Page 19 line number 384: bootstrapping in the traditional sense involves random re-sampling of the data with replacement (Efron, 1992). The procedure used in this manuscript is not bootstrapping in this sense, since genes are partitioned into 100 distinct groups and then the pseudotime orderings are evaluated on each mutually exclusive group of genes. Please consider using random re-samples of genes with replacement to align with the traditional notion of bootstrapping to estimate ordering.

7. Page 4 line number 90: to examine differentially expressed genes associated with bolting status, the plants in the bolted and non-bolted groups should be exchangeable (i.e. no differences across groups in plant type, growing conditions, chronological age, planting times, etc.). Were there any quantitative comparisons made between the two groups to explore this? It is stated that the plants are of the same type and were grown under uniform, controlled conditions, which is helpful. It would be more helpful to show comparisons if possible and add commentary to explain why it is reasonable that the two groups can be considered as exchangeable and how possible sources of non-uniform treatment were controlled during the experiment. Some of this already appears to be on page 16.

8. Figure 1B: The regression line shows the association between leaf size and biomass. However, there is no quantitative support provided here to demonstrate that leaf size and biomass are significantly larger for bolted plants. Hypothesis tests or significance of coefficients from a logistic regression model (bolting status ~ leaf size + biomass) would be helpful here.

9. Page 19 line number 401-402: Was there any investigation into the genes for which the GCV was high to see why the fit was poor? Since you are ordering based on the sum of normalized expression, perhaps these are genes with smaller normalized expressions that didn't influence the ordering as much?

10. Page 18 line number 371: what were the possible `alpha' values used for 5-fold CV?

Writing, structure, organization
1. Supplementary Figure 1A: It might be helpful to note that the genes have been ordered along the x and y axes according to the hierarchical clustering algorithm resulting in the block structures in the correlation matrix.

2. Page 5 line number 92, Figure 1, Supplementary Figure 3: what kind of multiple comparison adjustment is being used (e.g. Benjamini and Hochberg, 1995)? Also, what kind of multiple testing error is being controlled (e.g. false discovery rate)? These should be reported here and cited properly.

3. Figure 3C-3D: area under the curve and the acronym `AUC' is commonly used in statistics to refer to a measure of the accuracy of a classifier (area under the receiver operating characteristic curve) (Hanley and McNeil, 1982). To avoid confusion, it would be helpful to acknowledge this and explain that the notion of AUC used in this paper is different as it refers to area under the pseudotime expression curve.

4. Page 8 line number 136: It only makes sense to consider the largest positive coefficient if the variables are standardized, which is the case according to page 18 line number 358-359. Consider mentioning that the variables were standardized on Page 8 to avoid concern.

Minor typographical, grammatical, and visual issues:
• Abstract: "...high-resolution transcriptional time series."
• Page 8 line number 144: "...a high-resolution time series."
• Page 9 line number 160: "...contain information about a biologically meaningful developmental trajectories, such as..."

References:
Benjamini, Y., & Hochberg, Y. (1995). Controlling the false discovery rate: a practical and powerful approach to multiple testing. Journal of the Royal Statistical Society Series B: Statistical Methodology, 57(1), 289-300.

Efron, B. (1992). Bootstrap methods: another look at the jackknife. In Breakthroughs in statistics: Methodology and distribution (pp. 569-593). New York, NY: Springer New York.

Hanley, J. A., & McNeil, B. J. (1982). The meaning and use of the area under a receiver operating characteristic (ROC) curve. Radiology, 143(1), 29-36.

Kimes, P. K., Liu, Y., Neil Hayes, D., & Marron, J. S. (2017). Statistical significance for hierarchical clustering. Biometrics, 73(3), 811-821. Ramsay, J. O. (1988). Monotone regression splines in action. Statistical Science, 425-441.

*Please see a copy of our cover letter below:*

Thank you for your consideration of our manuscript entitled: 'Single-plant-omics reveals the cascade of transcriptional changes during the vegetative-to-reproductive transition'. We have incorporated most of the reviewer suggestions, which have improved the quality of the manuscript. These additional requested analyses have substantially strengthened our original claims. The most substantial changes are outlined below:

- Reviewer #2 (Comment **R2.2**) suggested that we investigate whether there were two population sub-groups which might have contributed to the heterogeneity in bolting times. We found that although there are several individual variants that are tightly associated with bolting (as previously shown in Figure 5A,B), **overall there is little evidence of two distinct sub-populations in our sample**. We discovered this by performing a very similar analysis to what Reviewer #2 did in their manuscript (Bhosale et al. (2013) Plant Cell) by: (1) performing a PCA on the variants and (2) building a bootstrapped neighbour-joining tree. The first PC only explained 10.8% of the variance in our sample. Additionally, the phylogenetic tree had weak bootstrapping support for separating out the bolting vs unbolting clades (less than 0.1%).
- To further confirm that our elastic net model predictions were not driven by the genetic structure of the population (also in reference to **R2.2**), we built complementary elastic net models that used variants as predictors, and this performed worse than our original analysis that used transcriptomes as predictors, confirming the value of our original analysis.
- To further confirm that our pseudotime inference is not simply capturing the presence of two distinct subpopulations (also in reference to **R2.2**), we clustered gene expression over pseudotime and found that many genes have sequential changes over expression, rather than 'big jumps' between bolted and unbolted samples. This further confirms that pseudotime is a useful approach.
- **R2.6** and **R3.6** suggested that we were too stringent in our criteria for filtering out outlier samples. We agreed with the reviewers and included three more samples in our analysis, bringing our total population size to 68. As a result, all of our figures have slightly changed, but all of the main conclusions have remained consistent.
- As per the suggestion of **R2.3**, we have also performed logistic regression to predict bolting status from the transcriptome. This is an additional analysis, but does not influence the main narrative.
- Two individuals who read our bioRxiv paper requested additional supplementary tables that we now include: (i) the raw physiological data (ii) the AUC values.

We thank the reviewers for their helpful suggestions and think that these changes have helped bolster our main conclusions and increased our confidence in our results. Please see the response to reviewers below. All line numbers refer to line numbers in the original manuscript. Please note that Supplemental figure numbers have changed from the original manuscript and we will refer to the new figure numbers in the text below. **Blue** indicates our comments and green indicates the modified text.

Best wishes,
Dr. Daphne Ezer
University of York

*********************

# Response to Editor (E)

The response letter asked for special consideration for some areas of reviewer concern.  In bold, we have highlighted where be have addressed these concerns:

**E.1:** There is an overriding concern that many of the results obtained may be skewed, because there may in fact be two genetically distinct subpopulations of plants incorporated in this study, rather than one genetically nearly homogeneous population. Therefore, it is important to provide convincing evidence that this is not the case **(see R2.2)**. Please also take special care to address all additional concerns about the stated assumptions and methodology **(see R2.4, R2.6, R2.7 and R3.1-R3.9)**.

**We have taken these concerns seriously and our changes have strengthened the manuscript, confirming our original claims.**

**E.2:** The Plant Cell now requires authors to complete and submit an author revisions checklist upon submission of a revised manuscript. The aim of the checklist is to aid authors in preparing a high-quality manuscript, facilitate the review and assessment of revised manuscripts, and help to ensure that journal standards are maintained across the board. If your manuscript is accepted, the completed checklist will be published as supplemental material attached to the article online. Please download a copy of the checklist (pdf fillable form) at this link, for submission with your revised manuscript: https://tpc.msubmit.net/html/Author_Revisions_Checklist.pdf.

**This is complete.**

**E.3:** Supplemental materials should be restricted to large datasets and tables, presentation of replicates, and validation of reagents, methods, or genotypes. Any data that are used to support the major claims must be in the main manuscript. Supplemental figure legends must indicate what figure in the main manuscript is supported by the supplemental data presented. Please justify how each of the supplemental figures meet the criteria.

**This is complete.**
**All supplemental figures now include the phrase 'Supports Figure …' to demonstrate which part of the main manuscript they are related to.  Please note that we also updated references to supplementary figures and data in the legends of Supplementary Figures 11 and 12.**

- **Supplemental Figure 1 explains the pre-processing of data for further analysis.**
- **Supplemental Figure 2 supports Figure 1 by showing the population structure between non-bolted and bolted plants.**
- **Supplemental Figure 3 supports the differential gene expression analysis, which was used to select genes for Figure 1A.**

- **Supplemental Figure 4 supports Figure 2 by contextualising the use of regulatory genes as predictors of biomass and leaf size.**
- **Supplemental Figure 5 supports Figure 3 by demonstrating the consistency of our pseudotime inference.**
- **Supplemental Figure 6 supports Figure 3 by explaining the process for smoothing gene expression over pseudotime and why genes were filtered out at this stage.**
- **Supplemental Figure 7 supports Figure 3 by demonstrating reasonable numbers of clusters showing the different categories of gene expression over pseudotime.**
- **Supplemental Figure 8 supports Figure 3 by showing that our data fit the assumptions for the "area under the curve" analysis.**
- **Supplemental Figure 9 supports Figure 3 by showing specific examples of genes to demonstrate how they fit the assumptions for the "area under the curve" analysis.**
- **Supplemental Figure 10 supports Figure 3 by showing the boundaries selected for large and medium-sized GO terms.**
- **Supplemental Figures 11 and 12 support Figure 3 by showing the full extent of AUC variation for all GO terms (not just the ones included for the main figure).**
- **Supplemental Figure 13 supports Figure 4 by demonstrating the weight of edges in the gene regulatory network, in the context of the less reliable predicted edges.**
- **Supplemental Figure 14 supports Figure 5 by demonstrating the genetic structure within the population - which provides context for the correlation between variants and pseudotime, biomass, and leaf size.**
- **Supplemental Figure 15 supports Figures 2 and 5 by demonstrating that variants are less predictive of biomass and leaf size, using an alternative method.**

**E.4:** Sampling methods and nature of "biological replicates" should be described precisely (i.e. different plants, parts of plants, pooled tissue, independent pools of tissue, sampled at different times, etc), along with a clear description of and rationale for any statistical analyses conducted. The reader should know exactly what was sampled; what forms the basis of the calculation of any means and statistical parameters reported. This is also necessary to ensure that proper statistical analysis was conducted.

**We analysed data from 68 individual plants (after removing two outliers) that are near isogenic and grown in near identical conditions. However, we only have one set of measurements per plant, and we treat each individual as a separate sample, so statistically we treat our samples as if there is only 1 biological replicate. The nature of how we selected the 68 plants is described on lines 318-321. To clarify that we treat each plant as an individual sample, we added the following text:**

Lines 318-321: Throughout this work, we refer to each plant individually (e.g. 'P002') – since gene expression and physiological measurements were measured separately per plant.

# Reviewer Comments:

## Reviewer #2 (R2):

**R2.1:** The authors present a study in which they use a single-plant -omics profiling strategy to unravel the timing of events during the vegetative-to-reproductive phase transition in Arabidopsis thaliana. The overall workflow of the study is very similar to the workflow followed in ref 9 (Cruz, De Meyer et al. (2020) Mol. Syst. Biol. 16, e9667), but focused on studying a particular developmental transition. The major innovative aspect of the study in my opinion is the use of a trajectory inference algorithm (as in single-cell data analysis) to order the individual plants along a pseudotime trajectory, which allows for inference of gene regulatory networks from the resulting pseudotime series data (in this case using DynGENIE3). For full disclosure: my group has similarly been using single-cell techniques such as trajectory and GRN inference on single-plant -omics data to study the vegetative-to-reproductive phase transition in Brassica napus, inspired by observations we made in a previous single-plant -omics study on field-grown rapeseed (De Meyer, Cruz et al. (2023) PLoS Comput Biol, https://doi.org/10.1371/journal.pcbi.1011161). We already presented results on this work at several seminars and conferences, but we are still about 3 months away from submitting the associated manuscript.

Perhaps not surprisingly given my background in single-plant -omics, I really like the concept of using subtle timing differences in a population of genetically nearly identical plants to study plant developmental processes. I'm also rather excited that the single-plant profiling concept, which I have been advocating for the past decade (Bhosale et al. (2013) Plant Cell, https://doi.org/10.1105/tpc.113.112268) is being picked up. I do however still have a number of comments on this manuscript.

**We thank the reviewer for being honest and open about potential conflicts of interest, and for providing some very useful feedback that has substantially increased the quality of our manuscript.  We agree that single-plant-omics is an under-utilised tool in the plant biology field and hope that both our work can help promote its further use.  We look forward to seeing his upcoming manuscript.  Please note that we have been presenting progress on this work since ICAR 2022.**

Major comments

**R2.2:** The PCA analysis in Supplemental Figure 6 (which I assume is based on all transcripts, although this is not clear from the methods or figure legends) shows two clear subgroups of plants. Similarly, the clusterings in Figure 1A, Supplemental Figures 1A and 2A suggest two subgroups, and in particular the SNP profiles in Figure 5B suggest that there may be two genetically different subgroups of plants in the dataset, with a substantial number of SNP profiles correlating strongly with gene expression profiles and the bolting status of the plants. If so, the single-plant character of the study may be compromised in the sense that the differences between subgroups may dominate/swamp the differences between individual plants in each subgroup, and many of the results obtained may have to be viewed as the result of a treatment vs control experiment with a lot of replicates, rather

than a single-plant omics experiment on a genetically nearly homogeneous population. We previously encountered similar population structure issues in both the ref. 9 study and the aforementioned Bhosale et al. (2013) Plant Cell study, and corrected for these (or at least their linear effects) before performing downstream analyses. In the present case, the effects of potential genetic substructure may be of particular concern given the strong correlation this substructure (if confirmed) may have with the bolting phenotype and hence the progress of the vegetative-to-reproductive phase transition under study. In the worst case, the population of plants could essentially consist of a group of plants of genotype 1 in which the vegetative-to-reproductive phase transition has barely started and another group of plants of genotype 2 where the transition has been finalized, so essentially an early and a late developmental timepoint with little in between (although this would also depend on the within-group variance in developmental timing). This may in turn jeopardize the pseudotime trajectory inference performed and its interpretation (see also comment 3). Subgroup structure may also affect e.g. the elastic net performances for the quantitative leaf size and biomass traits in Figure 2 if the subgroups would have substantially different leaf size and biomass on average. In order to check for genetic substructure in the population, you could perform SNP-based tree building and SNP-based PCA analysis, and visualize SNPs associated with any population substructure uncovered along the chromosomes, as in ref. 9.

**We thank the reviewer for this comment and pointing us to the supplements of Bhosale et al. (2013). Indeed, we have now performed a similar analysis as they did and are happy to report that there is <u>very weak evidence for the presence of two distinct sub-populations</u>.**

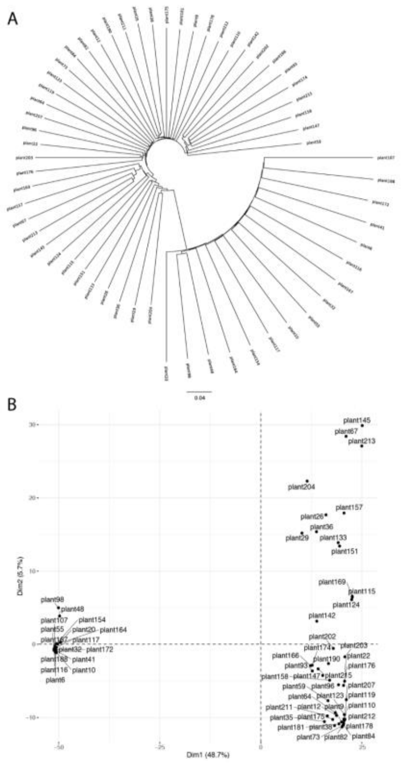


**Appendix Figure S1. SNP analysis of individual plants. (A)** Neighbor-joining tree for the individual plants based on biallelic SNPs in the RNA-seq data. Branch lengths are proportional to 1 - IBS (identity by state). **(B)** PCA plot for the individual plants based on their biallelic SNP profiles.

**We would like to address these concerns by first addressing two sub-questions: (i) What analysis have we now done to confirm that there is very weak evidence of two distinct sub-populations in our sample? (ii) Why did the reviewer suspect the presence of two sub-populations based on Supplemental Figure 6 and Main Figure 5B and why do these two graphs show such profound distinctions between bolted and non-bolted plants?**

**(i) We performed the two analysis performed by Bhosale et al. (2013) to analyse sub-population structure: (a) a PCA of the variants and (b) a phylogenetic tree. For comparison, please see the figure reported by Bhosale et al. (2013) which reported two sub-populations (Appendix Figure S1, included in the response**

**letter for clarity). In our version of this analysis (Supplemental Figure 14), we do not have a distantly separated lineage as in (Appendix Figure S1A) and our <u>bootstrap support for having the bolted plants in a separate clade is extremely weak (less than 0.1%)</u>. Moreover, Bhosale et al. (2013) reports that 46.7% of the variance in the SNP data could be explained by the first principal component (Appendix Figure S1B), which separated the data into two distinct clusters. While we see some weak clustering along the first principal component in our data, <u>this only explains 10.8% of the variance in our data</u>.**

**(ii) The reviewer points out two figures that seemed to suggest that there might be two distinct sub-populations, and we would like to suggest why these figures show such profound distinctions between bolted and unbolted plants: (a) Supplemental Figure 6: a PCA of the <u>transcriptomes</u> that showed that bolted and unbolted plants mostly clustered separately. It is quite likely that the clusters appear more strongly in the transcriptomics PCA plot, because the transcriptome shifts significantly at the onset of bolting. (b) Main Figure 5B which highlighted variants that were associated with bolting and showed two distinct sub-populations. In this figure, we specifically <u>chose to visualise variants that we found were associated with pseudotime,</u> and so we chose variants that would likely cluster by bolting status.**

**Even though there was only weak evidence of two sub-populations, we decided to perform two additional analysis to further validate that our conclusions were not substantially influenced by population substructure:**

- **Referring to the concern that subpopulation structure might "**jeopardize the pseudotime trajectory inference performed**": If pseudotime was only capturing information about two sub-populations, then we would not expect many genes to have gradual changes of expression over pseudotime. Instead, we would expect all or almost all of the genes to have a 'jump' in expression between the bolted and unbolted plants. We clustered the gene expression values and saw that many clusters had genes that displayed gradual changes in expression (see Supplemental Figure 8 and 9).**
- **Referring to the concern that subpopulation structure might impact "**the elastic net performances for the quantitative leaf size and biomass traits**": We performed the elastic net to predict biomass and leaf size based on the variants, which had R^2 values of 0.24 and 0.29, respectively, which was substantially worse than the corresponding models using transcriptomes.**

**Overall, we believe that these additional analyses support our hypothesis that the genetic substructure of the data does not confound our other results.**

*******************************************************************************************************

**Corresponding changes to the text:**

- **Supplemental Figure 14 added with the following legend:** "**A:** PCA plot of individual plants based on variants. Before PCA was performed, the homozygous reference allele was assigned 0; the heterozygous allele was assigned 1; and the homozygous alternate allele was assigned 2. **B:** Neighbour-joining tree on individual plants, followed by bootstrapping with 1000 repeats. Branch lengths represent the pairwise distance between samples – i.e. the number of variants where they differed.

The labels on nodes represent how often that branch was observed when repeating the neighbour-joining method." We found that the proportion of variance explained by PC1 was 10.8% (Supplemental Figure 14) - compared to 48.7% as reported in Appendix Figure S1 from ref. 9 (see below). In addition, we performed bootstrap analysis on the variant-based tree. Out of 1000 repeats, all branches were observed less than 400 times. Most of the branches which separated large numbers of non-bolted and bolted plants were observed 0 times. **Overall, this suggests there is weak evidence of two genetic subpopulations within the population.**

- **Supplemental Figure 8 added with the legend:** "After filtering for monotonic genes, each gene was assigned into one of 25 clusters. Gene expression was normalised between 0 and 1 for the AUC process. These clusters have been ordered to show decreasing clusters first (from lowest to highest mean AUC) then increasing clusters second. The mean AUC ranges from 23.6 to 38.8 for decreasing clusters and 35.5 to 45.1 for increasing clusters."
- **Supplemental Figure 7 added with the legend:** "The k-Shape clustering process was repeated for 5 fixed seeds, per predefined number of clusters (k). The points represent mean WCSS calculated across the 5 repeats per k (see Methods for details). Error bars represent the range from minimum to maximum WCSS per k. The blue dashed line represents the number of clusters chosen (k = 25) for Supplemental Figures 8 and 9, since the decrease in WCSS values after k = 25 is more gradual than before."
- **Supplemental Figure 15 added with the legend:** "**A, B:** The same as Figures 2A, B from the main text, except these models use variants as potential predictors. **C:** The same as Figure 2D from the main text, except these models use variants as potential predictors. The accuracy across all logistic regression models is 76.5%. Note: in all models, a number was assigned to each allele to be used as input data. Specifically, the homozygous reference allele was assigned 0; the heterozygous allele was assigned 1; and the homozygous alternate allele was assigned 2."
- **Corresponding updates to the Methods**:
    - Line 473: For analysis by PCA and as inputs to Elastic Net and logistic regression models, each haplotype was converted into an integer: the homozygous reference allele was assigned 0; the heterozygous allele was assigned 1; and the homozygous alternate allele was assigned 2. Elastic Net and logistic regression models were trained using cross-validation, as explained in 'Physiology trait prediction with Elastic Nets', except the hyperparameter 'l1_ratio' was allowed to vary to maximise prediction accuracy. Tree-building was performed using the ape package (version 5.7-1) in R, using the commands 'nj' for neighbour-joining, 'dist.gene' to count the number of differences in variants between samples, and 'boot.phylo' (with 'B = 1000') to repeat the analysis using bootstrapping (Paradis and Schliep, 2019).
    - Line 172: Clustering of genes indicates that different expression dynamics, corresponding to different AUC values, are represented in the filtered dataset (Supplemental Figures 7, 8; Methods). For the majority of genes, the residuals between the fit and the real data are consistent across pseudotime (Supplemental Figure 9).
    - Line 420: We clustered the monotonic genes using a shape-based metric from dtwclust (version 5.5.12), using the 'tsclust' function with 'distance' set to

‘sbd’ (Sardá-Espinosa, 2019). This is an implementation of the k-Shape algorithm (Paparrizos and Gravano, 2016). To select an appropriate number of clusters of visualisation, we calculated the Within Cluster Sum of Squares (WCSS), which is a metric to evaluate the tightness of clusters. [Followed by a definition of WCSS which cannot be rendered in plain text since it was written with Word’s equation editor.] We then selected k= 25 as the optimal number of clusters, after repeating the algorithm 5 times for values of k = 2, 3, …, 50 (Supplemental Figure 7).

**R2.3:** Figure 2 : why was bolting status not predicted from the transcriptome, next to leaf size and biomass ? This would be a classification rather than a regression problem but should be easy to do, and my guess is this classification model would actually perform much better even than the regression models shown for leaf size and biomass.

**We have added this analysis, using either the transcriptome or the variants to predict the biomass using a logistic regression model. This supplements our analysis, but does not have any repercussions on our main claims.**

*********************************************************************************************************

**Corresponding changes to the text:**

- Line 143: In addition, we used regularised logistic regression models to predict bolting status (see Methods). Similar to biomass models, these models had slightly higher accuracy when trained on only regulators, rather all genes (Figure 2D, Supplemental Figure 4C).
- Figure 2D has been updated to show a confusion matrix of the logistic regression results. The figure legend now reads: **D:** Confusion matrix of the logistic regression models trained to predict bolting status from gene expression. Predicted values were produced from a logistic regression model, trained on all data except for the relevant sample. Each model was regularised using the Elastic Net method. The accuracy across all logistic regression models is 85.3%. (See ‘Physiology trait prediction with Elastic Nets’ in Methods for more information on cross-validation, hyperparameter selection, and performance metrics.)
- Supplemental Figure 4C is similar to Figure 2D, but using the models trained on all genes. The figure legend now reads: **C:** The same as Figure 2D from the main text, except these models included all genes as potential predictors. The accuracy across all logistic regression models is 80.9%.
- Supplemental Figure 13C now includes results for logistic regression models trained to predict bolting status based on the genetic variants. The figure legend reads: **C:** The same as Figure 2D from the main text, except these models use variants as potential predictors. The accuracy across all logistic regression models is 76.5%. Note: in all models, a number was assigned to each allele to be used as input data. Specifically, the homozygous reference allele was assigned 0; the heterozygous allele was assigned 1; and the homozygous alternate allele was assigned 2.
- Tables S4, 5, 6 include information about the training of the logistic models.

**R2.4:** I appreciate the effort to build a trajectory inference algorithm tailored to the small size of the dataset at hand, but the approach followed has a number of drawbacks. First, the approach used assumes that all genes (or at least those that are DE in bolted versus non-

bolted plants) are monotonically increasing or decreasing, which is not necessarily the case in developmental trajectories (e.g. when they exhibit different intermediate stages). Second, and perhaps more importantly, the pseudotimes inferred in the end are essentially a ranking of samples from 1 to 65. This means the samples are spaced equidistantly over pseudotime, while in reality some samples may cluster closer together in developmental time than others (which may especially be the case if there would be plant subgroups, cfr. comment 1). This also has implications for downstream analyses such as the smoothing performed, the GRN inference with DynGENIE3 and in particular the discussion of changes in expression occurring earlier or later in the bolting process. Sentences like on line 174: '...pseudotime captures the continuous changes in gene expression of bolting-related processes, demonstrating that the transcriptional shift during bolting is not a binary process.' may not be accurate in this respect.

**This reviewer comment highlights a longstanding challenge in the wider discipline, and fixing this issue is well beyond the scope of this manuscript. All pseudotime metrics are in units that are inconsistent with traditional 'time' metrics. Gorin et al, (2022) highlights that pseudotime methods based on gene expression data are inherently 'phenomenological' and not associated with 'process time' and that this leads to challenges in interpretation (DOI:10.1371/journal.pcbi.1010492). In response to this concern, we have tempered our language in places, reflecting that we are identifying sequences of transcriptional events. We also decided to only perform GRN on monotonic genes, to minimise the impact of non-equidistant time, which removed several edges from the GRN.**

********************************************************************************************************************************************************************************
**Corresponding changes to the text:**

- Line 174: We observed that pseudotime captures the <u>sequence of changes</u> in gene expression within bolting-related processes.
- Line 251: One of the main benefits of the single-plant-omics approach is that it enables us to piece together the sequence of changes in what initially appears to be a switch-like transition process.
- Line 434: After pseudotime inference and filtering for monotonic genes, …

**R2.5:** I really appreciate the effort to try and make GRNs by analyzing the pseudotime series, but I miss a bit the biological interpretation of the GRN shown in Figure 4. Does the order of TFs increasing/decreasing over pseudotime make sense given what is known on the vegetative-to-reproductive transition in A. thaliana (and more particularly the transition processes happening in leaves rather than SAMs) ?

**We thank the reviewer for suggesting we add more biological interpretation of our GRN. The transcription factors which we find are consistent with those that change during the transition to senescence. Line 212:** We found that 15 out of 44 regulators identified in the GRN analysis were also found to be differentially expressed within a mature-to-senescent time series of leaf development (Woo et al., 2016) (Supplemental Table S8). These regulators included members of the NAC and WRKY transcription factor families: NAC13 (AT1G32870), NAC016 (AT1G34180) NAC WITH TRANSMEMBRANE MOTIF1

(NTM; AT4G01540), WRKY15 (AT2G23320), and WRKY25 (AT2G30250) (Olsen et al., 2005; Phukan et al., 2016).
**Note, this is different from the set of genes that are canonically involved in regulating the timing of reproductive growth (e.g. FLOWERING LOCUS T, FT) because the decision to flower occurs before bolting.**

**R2.6:** Line 337-338 : Five samples were discarded as outliers based on the fact that they form a separate cluster when the clustering dendrogram in Supplemental Figure 1C is cut at some (arbitrary ?) threshold. Only one of them (P128) can be considered a real outlier, removal of the other plant samples is unnecessary as their expression profiles are highly correlated with plenty of other samples (see Supplemental Figure 1A).

**We agree with the reviewer and a similar comment from the additional reviewer (Comment R3.6 below). We no longer remove samples based on hierarchical clustering. Instead, we performed PCA on log-transformed TPM values, to understand the overall structure of our data. Using this approach, we only observed one clear outlier, P196. This improvement means that most of the Figures, Supplemental Figures, and Supplemental Data have been updated to include three additional samples (P079, P098, and P169). Critically, this has not resulted in any significant changes to the main claims of the paper.**

**********************************************************************************************************

**Corresponding changes to the text and figures:**

- We updated Supplemental Figure 1 to show the new PCA. We have updated the legend for Supplemental Figures 1C and 1D to read: **C:** PCA of samples based on log-transformed TPM values, after the initial removal of sample P128 and low-expressed genes. One further sample was removed (P196), since it was far away from all other samples. Additionally, P158 and P158_rerun are adjacent in the reduced dimensionality space, justifying the replacement of P158 with P158_rerun. **D:** PCA of samples after final filtering, with the same conditions as **C**.
- Since Supplemental Figure 1D now shows a reduced dimensionality visualisation of the data, we removed (old) Supplemental Figure 6, which contained very similar plots. We removed "See Supplemental Figure 6 for a visualisation of these results using an unsupervised dimensionality reduction method (principal component analysis; PCA)." from lines 392 to 394.
- All main figures and supplemental Figures 2,3,5,6,10,11,12 and 13 updated to include the three additional samples. Supplementary Tables have been modified to reflect the revised values.
- The following legends of Supplemental figures have been modified:
  - Supplemental Figure 3. Legend has been updated to reflect the new numbers of differentially expressed genes: "Out of the 19 283 genes used in this analysis, we identified 6 967 down-regulated genes and 3 734 up-regulated genes (see Table S2)."
  - Supplemental Figure 6. Legend corrected to include the updated quantities: "**A:** Selection of optimal parameters for B-spline smoothing. Values correspond to the sum of GCV (generalised cross-validation) across all genes, after B-spline fitting with the fixed number of basis functions (n_basis) and the value of the second derivative smoothing penalty (lambda). Note: for

reference, the optimal parameters chosen were n_basis = 9 and lambda = 1390 (3 s.f.). **B**: Selection of threshold based on GCV value of each gene. All genes which passed initial filtering are displayed. Genes with high GCV are assumed to not vary over pseudotime and they are therefore removed before further analysis. We selected the 4000 genes with lowest GCV (indicated by the vertical line). **C**: Selection of threshold for interquartile range. Some genes had low GCV, despite displaying poor behaviour over pseudotime (for example, only 1 or 2 samples with high TPM). These were removed by only keeping genes with a low range to interquartile range ratio. Genes with a ratio higher than 6.75 were removed (indicated by the dashed red line). The solid red line corresponds to AT5G04380. **D**: An example of a gene (AT5G04380) removed by the previous filtering. This gene had a ratio of 22.3 (3 s.f.). The purple line is the B-spline smoothed curve and the points represent TPM values in each sample."

- Quantitative values listed in the main text have been modified to reflect the modified values after the addition of the three additional samples:
    - Line 92: Overall, 3734 genes were up-regulated, and 6967 genes were down-regulated after bolting, accounting for 55% of the entire transcriptome…
    - Line 219: …we identified a total of 1047 high-confidence variants…
    - Line 337: …One sample (P158) was sequenced again due to low read depth in the initial sequencing and the initial data was discarded. Two samples (P128 and P196) were discarded as outliers during initial clustering of the data (Supplemental Figure 1)....This left 19 283 genes for further analysis...
    - Line 407: 3 906 genes remained after these filtering steps.
- We chose to highlight some new genes in the relevant section (which used to be in the Supplemental Text and is now in the Results), given some changes in the coefficients of our elastic net models. Line 134 onwards: "We were able to identify genes that were consistently identified as predictors of both leaf area and biomass in multiple models during the LOOCV process (Figure 2C, Table S5). Overall, 180 regulatory genes had a non-zero median coefficient across all models (Table S5). Of these, 8 were identified as positive predictors of biomass and leaf size and 13 were identified as negative predictors of both traits (Table S5). The gene with the highest positive coefficient for both traits was *HOMEOBOX PROTEIN 54* (*HB54*; AT1G27045), involved in a nitrogen-signalling cascade linked to plant growth (Ariga et al., 2022). An auxin-responsive gene, *INDOLEACETIC ACID-INDUCED PROTEIN 10* (*IAA10*; AT1G04100), was identified as a positive regulator of leaf size and biomass (Liscum and Reed, 2002). We identified three AGAMOUS-LIKE MADS-box TFs with high positive or negative coefficients for these traits. Orthologs of similar TFs in rapeseed were highlighted in a single-plant-omics study as predictors of yield phenotypes (De Meyer et al., 2023). *SEPALLATA 4* (*SEP4*; AT2G03710), also known as *AGAMOUS-LIKE 3* (*AGL3*), had a high positive coefficient for biomass and for leaf models. *AGAMOUS-LIKE 16* (*AGL16*; AT3G57230) had a high positive coefficient for leaf size but not biomass. *AGAMOUS-LIKE 19* (*AGL19*; AT4G22950), which is a known component in the *FLC*-independent vernalization pathway, had a highly negative median coefficient for biomass models but not leaf models (Schönrock et al., 2006). Interestingly, *CURLY LEAF* (*CLF*; AT2G23380) also had a highly negative median coefficient for biomass models but not leaf models. *CLF* represses expression of *AGL19* in an age-related manner (Schönrock et al., 2006).

Additionally, *CLF* has been shown to control leaf morphogenesis (Kim et al., 1998). We identified genes with highly negative coefficients for both traits. *TARGET OF EARLY ACTIVATION TAGGED (EAT) 2* (*TOE2*; AT5G60120) is a highly negative predictor of both traits, suggesting that there is a link between leaf size, biomass, and the microRNA/SPL-mediated juvenile to mature transition (Werner et al., 2021). Other highly negative predictors of both traits include transcription factors controlling stomatal development (*SOL1/TCX3; SOL, TSO1-like; TCX, TSO1-like CXC*; AT3G22760) and shade avoidance response (*PHYTOCHROME-INTERACTING FACTOR 7; PIF7*; AT5G61270) (Simmons et al., 2019; Galvão et al., 2019). We also sought to understand important predictors of one trait which were not predictive of the other trait. For leaf size, strongly positive predictors included: an ERF TF family gene (*ERF018*; AT1G74930); a positive regulator of photomorphogenesis and regulator of flowering (*CONSTANS-LIKE 3, COL3*; AT2G24790); and, as mentioned above, *AGL16* (Nakano et al., 2006; Liu et al., 2021; Tripathi et al., 2017). Conversely, *MYB-SHAQKYF 1* (*MYS1*; AT2G38300), a transcription factor involved in leaf wax biosynthesis, was identified as a strongly negative predictor of leaf size but not biomass (Liu et al., 2022). For biomass, strongly positive predictors included: a bZIP TF family gene, identified as affecting germination (*bZIP44*; AT1G75390); *SQUAMOSA PROMOTER BINDING PROTEIN-LIKE 4* (*SPL4*; AT1G53160), which is repressed by *miR156*; and *RGA-LIKE 1* (*RGL1*; AT1G66350), which is a negative regulator of gibberellin responses (Iglesias-Fernández et al., 2013; Xu et al., 2016; Wen and Chang, 2002). Conversely, strong negative predictors of biomass included *CLF* and *AGL19*, as mentioned above."

**R2.7**: line 347: it is unclear where the CPM counts come from, and whether they were properly normalized across samples (as done e.g. in the edgeR or DESeq2 protocols). Salmon does not produce CPMs to my knowledge. The Salmon TPMs are likely also not the best possible expression measures to use for e.g. training elastic nets, even after z-scoring, see e.g. https://doi.org/10.1186/s12967-021-02936-w . Am I right in assuming that these TPM values were not log-transformed for use in e.g. the elastic nets?

**We thank the reviewer for highlighting the ambiguity in our use of "CPM". Salmon produces a value called 'NumReads' which is an estimate of the number of reads corresponding to a transcript. We used this to calculate CPM values. We clarify this in the text.**

**However, we do not believe that the cited manuscript provides convincing evidence that our use of TPMs is inappropriate:**

- **In brief, this is not a robust benchmarking study, but rather an analysis of a single data set of human-derived cells.**
- **They do not assess the impact of normalisation strategies on a prediction task (like elastic net), but rather they focus on correlation between samples and hierarchical clustering. Therefore the context is very different.**

**In contrast, we believe that our normalisation strategy was appropriate for our elastic net analysis for the following reasons:**

- **Our predictive success in LOOCV suggests that we can make accurate predictions on data that was not part of our training set.**

- **The presence of large numbers of differentially expressed genes breaks the assumption of TMM normalisation (which is used in edgeR and DESeq) that most genes do not change their expression values.**
- **TPM is a more easily interpretable value than TMM, as the units are biologically meaningful– i.e. transcripts per million.**

**************************************************************************************************
**Corresponding change to the text:**

Line 346: "However, since we expected more than 30% of the transcriptome to be differentially expressed, we did not perform the trimmed mean of M values (TMM) normalisation (Robinson and Oshlack, 2010). Counts per million (CPM) were used as inputs to this test and p-values were corrected according to the Benjamini and Hochberg method to control the False Discovery Rate (Benjamini and Hochberg, 1995). Salmon produces 'NumReads' as an estimate of the number of reads corresponding to a transcript, so these values were combined to the gene-level (as above) and then normalised to produce CPM values (Patro et al., 2017)."

Minor comments

**R2.8:** Line 79-80: 'large population' : please give a number of plants

Line 79: (n = 68 after filtering; see Methods, Supplemental Figure 1)

Some statements in the text are not adequately supported by statistics :
**R2.9:** Line 87-88: '...plants with the same bolting status had more closely correlated transcriptomes than plants with differing bolting statuses.'

Line 87 is now more precise: We performed hierarchical clustering of plants, based on the Pearson correlation of gene expression between samples (Supplemental Figure 2). This analysis showed that the samples can be separated into two clusters. One cluster contains the majority of the non-bolted plants (35 out of 45) and the other contains the majority of the bolted plants (18 out of 23)

**R2.10:** Line 193-194: 'Interestingly, the term plasmodesma decreases significantly earlier than chloroplast,'

Line 193 now references the supplementary table containing p-values. We chose not to list all associated p-values in the text itself, because this broke the flow of the text: "(Figure 3D; Table S8)."

**R2.11:** Table S2 : it is unclear whether p-values have been adjusted for multiple testing or not (I assume yes)

**Yes - the p-values have been adjusted for multiple testing, as performed by default by gProfiler. We have made the p-value correction explicit. (Additionally the citation has been updated, as we used a newer version of the g:Profiler).**

Line 351: "GO term overrepresentation was performed using the gprofiler2 R package (version 0.2.1) (Kolberg et al., 2020). This called the g:Profiler server (version e111_eg58_p18_30541362), which utilises the g:SCS multiple testing correction method, and we then applied a significance threshold of 1e-4 (Kolberg et al., 2023) (Table S3)."

**R2.12:** line 139-140 and first section of supplemental material: it would be interesting to compare the predictors found and the processes they are involved in (in particular for leaf area) with those found in rapeseed in a similar setup at a comparable developmental time point (vegetative-to-reproductive phase transition), see De Meyer, Cruz et al. (2023) PLoS Comput Biol, https://doi.org/10.1371/journal.pcbi.1011161 .

**We thank the reviewer for suggesting this paper as a relevant study. We have updated the relevant section (now in the main results from Line 134) to match the new results from the Elastic Net analysis (see the response to comment R2.6). In particular, we have noted that a few predictors overlap with those identified in this paper:** We identified three AGAMOUS-LIKE MADS-box TFs with high positive or negative coefficients for these traits. Orthologs of similar TFs in rapeseed were highlighted in a single-plant-omics study as predictors of yield phenotypes (De Meyer et al., 2023).

**We also updated the discussion to note this. Line 244:** These included members of the AGAMOUS-LIKE MADS-box transcription factor family, whose orthologs in rapeseed have been highlighted as predictors of yield phenotypes in a recent single-plant-omics study (De Meyer et al., 2023).

**R2.13:** Supplemental Figure 7 legend : 'Genes with a ratio lower than 6.75' : lower should be higher.

**This has been done.**

**R2.14:** Supplemental Figure 8 legend : 'we first needed to select GO terms of approximately the same size' : why is this ?

**We added more detail in Line 422 to justify why we made this choice**: We compared GO terms which had similar numbers of genes (Table S10, Supplemental Figure 10), to avoid comparing very detailed GO terms with fewer labelled genes (such as 'cellular response to DNA damage stimulus') with broader GO terms (such as 'translation').

**We also made some minor rewordings of the legend of Supplemental Figure 10 to make it clearer:** R2.7d: "In order to compare 'area under the curve' values between groups of GO terms, we first needed to select GO terms of approximately the same size (in terms of the genes which passed all previous filtering steps). Thus, we manually selected thresholds for 'large' (depending on condition, red line) and 'medium' (depending on condition, purple line) GO terms (Table S7). Also, these values needed to be chosen independently for decreasing and increasing genes. **A**: Thresholds chosen for decreasing genes, per GO term. **B**: Thresholds chosen for increasing genes, per GO term"

Steven Maere

# Reviewer #3 (R3):

Review: Single-plant-omics reveals the cascade of transcriptional changes during the vegetative-to-reproductive transition

**R3.1:** This article uses RNA-seq on leaves from bolted and unbolted plants to identify differentially expressed genes and those associated with leaf size and biomass. A pseudo-time ordering of plants based on sum of standardized gene expressions is developed and used to estimate and compare time trends in gene expressions associated with the bolting-related processes.

The paper is well-written and nicely organized. There is a wealth of analytical results provided and the authors are to be commended on making the data and code publicly available for reproducibility purposes. The code is well documented and includes instructions for setting up the environment used to conduct the analysis to facilitate reproduction. Below are some comments that need to be addressed regarding the methodology and analytical results related to the smoothness and monotonicity assumptions, modeling approaches used to achieve smooth and monotonic time trends, statistically significant clusterings, and bootstrapping. There are also minor comments on the writing and structure.

**We wanted to particularly thank the reviewer for taking the time to not only review our manuscript but also review the documentation of our code and data, as this is something that is really important for reproducibility and is often overlooked in the review process.**

Methodological and analytical
**R3.2:** Page 10 line number 164-169: It is hard to understand why having a majority of genes being differentially expressed during bolting would be able to serve as the basis for the assumption that the change in expression levels is monotone over pseudotime. Of course, assumptions are necessary, especially when sample sizes are small, but this justification for the assumption seems weak. While fitting a monotone trend is probably the best one could hope to accomplish given the limited data, there could be many possible non-monotone trajectories that could lead to changes in expression associated with bolting. Are there any longitudinal studies that can be referenced to further justify this assumption? Are there any mechanistic explanations that could also justify this assumption? Since this assumption is the basis for much of the methodology and results presented, it is worth a closer look.

**We clarified the bases for our assumption on Lines 167 - 169, which is supported by prior literature:** “Our first basis for making this assumption was our observation in Section 1 that the majority of genes are differentially expressed during bolting, suggesting that they primarily increase or decrease in expression. Our second basis was that previous longitudinal studies of leaves show that many genes change their expression monotonically over development (Breeze et al., 2011; Woo et al., 2016).”

**R3.3:** Results Section 3 page 10 mentions that the ordering to generate the pseudo time series of gene expression relies on a monotonicity assumption, and the Discussion on page 14 says the ordering relies on a smoothness assumption. These are very different types of

assumptions. A function can be smooth but not monotone and vice versa. If both are needed, both need to be clearly stated.

**The reviewer is correct that it is important to make sure the assumptions are clear. However, we could not find the place in our discussion where we claim that our pseudotime ordering relies on a smoothness assumption (which would have been incorrect– we only rely on monotonicity). Nevertheless, our textual changes in response to reviewer #2 (R2.4) should have removed any confusion, as we have increased the clarity and precision of the text in question.**

**R3.4:** Page 19 line number 395-408: The B-spline basis will not enforce the monotonicity assumption described on page 10 and will lead to non-monotone (but smooth) gene expression trajectories, some of which can be seen in Figure 4B-4C. Monotone spline bases, like I-splines (Ramsay, 1988), should be used to achieve both smoothness and monotonicity. This will align your analyses with your assumptions and avoid the need to filter out non-monotonic gene expression trajectories (page 20 line number 410-420).

**This is a very intriguing suggestion; however, it is inappropriate for our particular analysis. Critically, our assumption is not that <u>all</u> genes monotonically change over pseudotime, only that <u>a large proportion do.</u> If we were to use an I-spline, then we would be forcing non-monotonic genes to appear to have monotonic expression patterns, which would not be an appropriate way to represent the data. Indeed, our filtering out of non-monotonic gene expression trajectories is a benefit of our approach, because our aim was to find a smaller set of genes that monotonically change over pseudotime, as these are more likely to be bolting-associated genes.**

**R3.5:** Supplementary Figure 1B-1C: How was the low expression threshold determined?

**Low expression threshold was determined by the 'elbow' technique - i.e. at a value where the gradient before is much steeper than the gradient after. This is an acceptable approach and actually goes beyond what most papers do for identifying a threshold (i.e. picking an arbitrary threshold with no justification). See Jung et al (2016) in *Science* for one such example.**

**R3.6:** How was the height of 11,500 determined in order to remove the further samples? There are methods that can be used to determine the statistical significance of a particular clustering given a dendrogram (Kimes et al., 2017). Please consider using this for identifying meaningful clusterings.

**This is a very important point, which we agree with. Indeed, this was also pointed out in R2.6 and as a result we have added three additional samples and repeated our analysis. This has not impacted our main claims, but has led to many minor changes throughout the text, as outlined above in R2.6.**

**R3.7:** Isn't it possible that a gene with a quick increase in pseudotime can have a similar AUC to a gene exhibiting a slow decrease in pseudotime? If so, then genes should be grouped by increasing vs. decreasing expression levels and analyzed separately.

According to Page 19-20 line number 382-383, down-regulated genes were transformed (1-x), which should make the trends all going up over pseudotime, right? Wouldn't that change the interpretation of the AUC calculation, since a slow decrease (which should have large AUC) would be transformed to a quick increase (which will have a small AUC)? Please clarify.

**Thank you for pointing out that this wasn't expressed as clearly as it should have been. In the Method section entitled: 'Analysis of gene expression over pseudotime' (lines 418-420), we wrote:** "For increasing genes, the AUC was calculated for (1 - normalised gene expression) values."

**We performed this operation so that a lower AUC value would be associated with early-changing genes (and vice versa), independent of whether the gene was increasing or decreasing. We have now expanded the text to highlight this point, since it is essential for the understanding of the results:**

Lines 418 to 420 now read: "Crucially, since these selected genes are monotonic, a gene with smaller AUC changes more rapidly earlier on in pseudotime (see Figure 3C). For increasing genes, the AUC was calculated for (1 - normalised gene expression) values, so that a smaller AUC also indicated changes earlier on in pseudotime."

**R3.8**: Page 19 line number 384: bootstrapping in the traditional sense involves random re-sampling of the data with replacement (Efron, 1992). The procedure used in this manuscript is not bootstrapping in this sense, since genes are partitioned into 100 distinct groups and then the pseudotime orderings are evaluated on each mutually exclusive group of genes. Please consider using random re-samples of genes with replacement to align with the traditional notion of bootstrapping to estimate ordering.

**Thank you for the comment. We believe that using 100 distinct groups of genes is sufficient to demonstrate that pseudotime inference is consistent across the entire collection of genes. However, we do accept that this approach is not bootstrapping, so we have updated the text accordingly.**

Line 169: "We were able to consistently order our plants along pseudotime to maximise the monotonicity of gene expression, using distinct subsets of genes. Finally, we compiled a consensus pseudotime of samples (Methods; Supplemental Figure 5)." In the methods, lines 383-385 now read: "To validate this assumption, we partitioned genes into 100 distinct groups and repeated the pseudotime inference method for each group (Supplemental Figure 5)."

**R3.9:** Page 4 line number 90: to examine differentially expressed genes associated with bolting status, the plants in the bolted and non-bolted groups should be exchangeable (i.e. no differences across groups in plant type, growing conditions, chronological age, planting times, etc.). Were there any quantitative comparisons made between the two groups to explore this? It is stated that the plants are of the same type and were grown under uniform, controlled conditions, which is helpful. It would be more helpful to show comparisons if possible and add commentary to explain why it is reasonable that the two groups can be

considered as exchangeable and how possible sources of non-uniform treatment were controlled during the experiment. Some of this already appears to be on page 16.

**We agree with the reviewer that it is extremely important that bolted and non-bolted plants should be exchangeable. Indeed, we have kept the plant type, growing conditions, chronological age, and planting times as similar as possible.**

**We also shuffled the positions of the plants approximately every three days to control for positional effects within the growth chambers, and we have added this information to the methods.**
Line 317: "Throughout growth, plants were shuffled within the growth chambers approximately every 3 days."

**Another potential source of non-exchangeability is population substructure, which we address fully in response to R2.2.**

**R3.10:** Figure 1B: The regression line shows the association between leaf size and biomass. However, there is no quantitative support provided here to demonstrate that leaf size and biomass are significantly larger for bolted plants. Hypothesis tests or significance of coefficients from a logistic regression model (bolting status ~ leaf size + biomass) would be helpful here.

**We performed non-parametric Mann-Whitney U tests to demonstrate that leaf size and biomass are significantly larger for bolted plants than non-bolted plants. We have restated these claims from Line 120 in the results:** We observed that biomass and leaf size were correlated across the population (linear regression, $R^2$ = 0.55, p = 1.59e-12). Additionally, bolted plants had significantly higher biomass (Mann-Whitney U test, W = 111.5, p-value = 1.46e-7) and leaf size (Mann-Whitney U test, W = 185, p-value = 6.33e-6) than non-bolted plants (Figure 1B, Table S1).

**R3.11:** Page 19 line number 401-402: Was there any investigation into the genes for which the GCV was high to see why the fit was poor? Since you are ordering based on the sum of normalized expression, perhaps these are genes with smaller normalized expressions that didn't influence the ordering as much?

**We agree with the reviewer that it is always important to look through some individual examples – in this case gene expression over pseudotime – in order to feel confident that a statistical approach is doing what is intended. Indeed, we see from the examples below that genes with higher GCV have high levels of noise, which implies that the predicted smoothing values are less meaningful. However, we have chosen not to include this in the manuscript, as it does not seem to provide any particular insight into the biological story.**

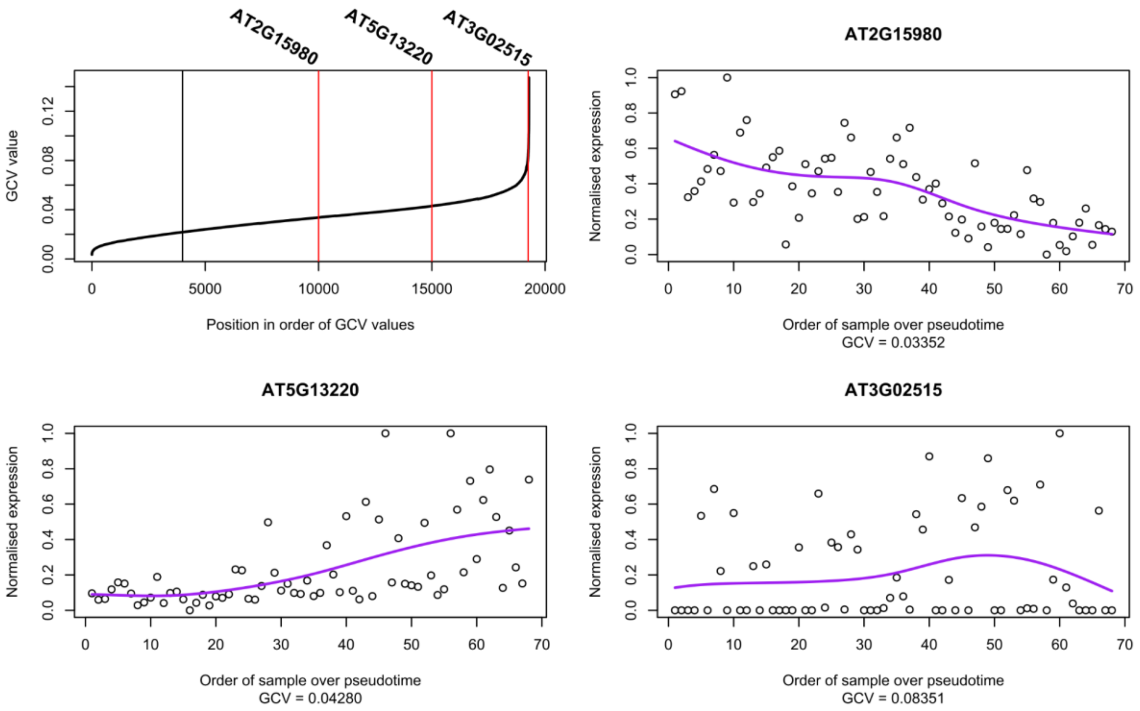


**We'd like to particularly draw your attention to the fact that we initially removed lowly expressed genes and normalised gene expression prior to pseudotime analysis, i.e. by truncating individual samples with very high or low TPM and then scaling gene expression to be between 0 and 1 (see 'Pseudotime inference' in Methods). Together, this ensured each gene had an equal contribution to pseudotime inference.**

**R3.12:** Page 18 line number 371: what were the possible `alpha' values used for 5-fold CV?

**The package we used to find optimal 'alpha' values, 'scikit-learn', does not require pre-specified potential 'alpha' values (Zou and Hastie 2005). By default, for each fixed 'l1_ratio', a path of up to 100 possible 'alpha' values are searched. We have clarified this in Physiology trait prediction with Elastic Nets:**
Line 372: "Parameters relating to `alpha` were kept at their default values."

Writing, structure, organization
**R3.13:** Supplementary Figure 1A: It might be helpful to note that the genes have been ordered along the x and y axes according to the hierarchical clustering algorithm resulting in the block structures in the correlation matrix.

**The legend of Supplemental Figure 1A now reads**: "Rows and columns of the heatmap have been reordered according to hierarchical clustering."

**R3.14:** Page 5 line number 92, Figure 1, Supplementary Figure 3: what kind of multiple comparison adjustment is being used (e.g. Benjamini and Hochberg, 1995)? Also, what kind of multiple testing error is being controlled (e.g. false discovery rate)? These should be reported here and cited properly.

**This information has been added in response to R2.2.7**

**R3.15:** Figure 3C-3D: area under the curve and the acronym `AUC' is commonly used in statistics to refer to a measure of the accuracy of a classifier (area under the receiver operating characteristic curve) (Hanley and McNeil, 1982). To avoid confusion, it would be helpful to acknowledge this and explain that the notion of AUC used in this paper is different as it refers to area under the pseudotime expression curve.

We added the following sentence after Line 181 to make this clear: "Note, this should be distinguished from the commonly used Area Under the ROC (Receiver Operating Characteristic) Curve statistic, that is used to judge performance of diagnostic tests (Hanley and McNeil, 1982)."

**R3.16:** Page 8 line number 136: It only makes sense to consider the largest positive coefficient if the variables are standardized, which is the case according to page 18 line number 358-359. Consider mentioning that the variables were standardized on Page 8 to avoid concern.

Line 135: "Since gene expression was standardised before input to all models, it was possible to compare the coefficients of different genes within a model."

**R3.17:** Minor typographical, grammatical, and visual issues:
• Abstract: "...high-resolution transcriptional time series."
• Page 8 line number 144: "...a high-resolution time series."
• Page 9 line number 160: "...contain information about a biologically meaningful developmental trajectories, such as..."

**Done**

References:

Benjamini, Y., & Hochberg, Y. (1995). Controlling the false discovery rate: a practical and powerful approach to multiple testing. Journal of the Royal Statistical Society Series B: Statistical Methodology, 57(1), 289-300.

Efron, B. (1992). Bootstrap methods: another look at the jackknife. In Breakthroughs in statistics: Methodology and distribution (pp. 569-593). New York, NY: Springer New York.

Hanley, J. A., & McNeil, B. J. (1982). The meaning and use of the area under a receiver operating characteristic (ROC) curve. Radiology, 143(1), 29-36.

Kimes, P. K., Liu, Y., Neil Hayes, D., & Marron, J. S. (2017). Statistical significance for hierarchical clustering. Biometrics, 73(3), 811-821. Ramsay, J. O. (1988). Monotone regression splines in action. Statistical Science, 425-441.

1st Revision - Editorial Decision13-May-2024

Dr. Daphne Ezer
University of York
Biology
Heslington YO310RT
United Kingdom

Dear Dr. Ezer:

We have received reviews of your manuscript entitled "Single-plant-omics reveals the cascade of transcriptional changes during the vegetative-to-reproductive transition." Thank you for submitting your best work to The Plant Cell. The editorial board agrees that the work you describe is substantive, falls within the scope of the journal, and may become acceptable for publication, pending revision and potential re-review.

The revised manuscript was greatly improved and now addresses all of Reviewer #3's concerns. However, Reviewer #2 has a number of detailed questions and concerns remaining, some of these relate to the running of analysis scripts, and we feel that it's important to have the opportunity to address all of these.

Please contact us if there are ambiguous comments or if you wish to discuss the revision.

Given the nature of the comments, we are offering you 60 days from when we have issued this decision to complete the revision. If a revision is not returned within this time frame, and if you have not been granted an extension, we will withdraw the manuscript, which will leave you free to submit the work elsewhere. If you need an extension, we encourage you to contact us at any point before the 60 days have passed.

When you are ready to submit the revised version, please upload a highlighted copy that indicates all changes made in response to the editor and reviewer recommendations. Include an itemized list of all changes made in response to each of the reviewer's suggestions in the "Response to Reviewers" section; please note that reviewers do not have access to your cover letter, nor was this decision letter shared with them.

Thank you for the privilege of reviewing your work. We look forward to receiving your revised manuscript.

Sincerely,

The Plant Cell Editorial Board

**Please note the following:**

**-The Plant Cell now requires authors to complete and submit an author revisions checklist upon submission of a revised manuscript. The aim of the checklist is to aid authors in preparing a high-quality manuscript, facilitate the review and assessment of revised manuscripts, and help to ensure that journal standards are maintained across the board. If your manuscript is accepted, the completed checklist will be published as supplemental material attached to the article online. Please download a copy of the checklist (pdf fillable form) at this link, for submission with your revised manuscript: https://tpc.msubmit.net/html/Author_Revisions_Checklist.pdf.**

**-Supplemental materials should be restricted to large datasets and tables, presentation of replicates, and validation of reagents, methods, or genotypes. Any data that are used to support the major claims must be in the main manuscript. Supplemental figure legends must indicate what figure in the main manuscript is supported by the supplemental data presented. Please justify how each of the supplemental figures meet the criteria.**

**-Sampling methods and nature of "biological replicates" should be described precisely (i.e. different plants, parts of plants, pooled tissue, independent pools of tissue, sampled at different times, etc), along with a clear description of and rationale for any statistical analyses conducted. The reader should know exactly what was sampled; what forms the basis of the calculation of any means and statistical parameters reported. This is also necessary to ensure that proper statistical analysis was conducted.**

**-Want to add this revision deadline to your calendar? Click below!**

------------------------------------------------------------------------- Reviewer comments:

Reviewer #2 (Comments for the Author):

Dear Authors, Dear Editor,

I focused my review of the revised manuscript version on the potential subpopulation issue I flagged in my previous review, as I think resolving this potential issue is of primary importance and my other comments were generally addressed well (although I still believe the pseudotime inference algorithm used by the authors is suboptimal).

The authors performed the analyses suggested in my previous review to check for distinct subpopulations in their sample of individual plants. In my opinion, not all the conclusions they draw from these analyses are equally well-supported however. While I agree that the SNP-based PCA results and the SNP-based phylogeny look less dramatic than in Cruz et al. (2020) (erroneously referred to by the authors as Bhosale et al. (2013)), there still seems to be a clear substructure in both of them.

Regarding the PCA, the authors bring up that their PC1 only explains 10.8% of the variance, while the PC1 in Cruz et al. (2020) explained 48.7% of the variance. The authors take this as evidence that in their case the clustering seen along PC1 is weak. There's still a clear separation in Supplemental Figure 14A along the first PC however, and that this PC only explains 10.8% of the variance in the SNP dataset primarily indicates that their population structure is more complex than the one in Cruz et al. (2020), not that it is not important or influential.

That the population structure is more complex is also suggested by the structure of the phylogenetic tree in Supplemental Figure 14B (what is the unit of branch lengths here ?). There's a distinctive clade containing 18/23 bolted plants, which likely corresponds to the left 'bolted' cluster on the PCA plot (although this cannot be assessed with certainty because the PCA plot is unlabeled). On the other hand, the bootstrap support for this clade is really low, which is a bit at odds with the clear signal on the PCA plot. Could the authors elaborate more on how this tree has been made ? From the methods, I got that 'dist.gene' in the ape package was used as a distance, which should be the number of variant differences between samples. Was this dist.gene calculated on the 0-1-2 haplotype profiles, or directly on the VCF data somehow ? I was also a bit confused by the statement on line 288 that 'Bootstrapping analysis of a neighbour-joining tree trained on variants showed that subsets of variants predicted very different phylogenetic structures (Supplemental Figure
14B).', in the sense that bootstrapping is not the same as subsampling.

The authors state that 'the bootstrap support for having the bolted plants in a separate clade is extremely weak (less than 0.1%)', but I'm not sure what this means. That not all bolted plants belong in a separate clade indeed readily seen already from the PCA, but this is not a strong argument for there being no relationship between population structure and bolting status. Even if not all bolting plants belong to a separate clade, if 18/23 of them do there is likely a relationship between population structure and bolting status. That this clade is not 'a distantly separated lineage' is of little consequence in this matter, as there might be only one or a few causative SNPs involved (possibly along with linked SNPs with no effect). What really matters is whether or not the bolted plants are found clustered in both the PCA and the phylogenetic tree.

In order to further assess whether or not there may be significant genetic subpopulations of plants in the data, I performed a number of analyses on the VCF file the authors provide as Supplemental Table S14. A first analysis using TASSEL5 (as in Cruz et al. 2020) using 1-IBS (identity by state) as the distance measure produced a tree (Figure R1) that was even less informative than the one produced by the authors using ape (TASSEL does not provide bootstrap values), but it did show the same separation in two clades, with the top clade in Figure R1 corresponding to the 'bolted' clade in Supplemental Figure 14B. I also tried SNPhylo as an alternative SNP-based tree building program (maximum likelihood), but unfortunately got the message 'VCF file (/36604_1_supp_1447730_sbsbx2.vcf) is too small to run this script!'.

Figure R1. Archaeopteryx visualization of midpoint-rooted TASSEL5-inferred SNP tree based on filtered VCF file (SNPs that are heterozygous in all individuals removed, distance measure = 1-IBS). The top cluster contains the same samples as the 'bolted' cluster in Supplemental Figure 14B.

As an alternative, I used the SNPRelate R package to perform an identity-by-state analysis on the VCF file in Supplemental Table S14 (without filtering) and to perform a clustering analysis on the resulting pairwise IBS distance matrix, which uses permutation Z-scores to automatically determine subgroups (Figure R2). This analysis did identify a significant split in two subgroups, with one of them identical to the 'bolted' clade in Supplemental Table S14.

**The R commands used for this analysis were the following (see also tutorial at the bottom of the following notebook : https://rpubs.com/adel922/560260) :**

```
library(gdsfmt)
library(SNPRelate)

vcf.fn <- "/36604_1_supp_1447730_sbsbx2.vcf"
snpgdsVCF2GDS(vcf.fn, "test.gds", method="biallelic.only")
# Summary
snpgdsSummary("test.gds")
genofile <- snpgdsOpen("test.gds")
set.seed(100)
ibs.hc <- snpgdsHCluster(snpgdsIBS(genofile, num.thread=2))
# Determine groups of individuals automatically
rv <- snpgdsCutTree(ibs.hc)
plot(rv$dendrogram, leaflab="perpendicular", main="SNPRelate tree")
```

**Figure R2. SNPRelate hierarchical clustering tree. The Z score of 23.9 indicates that 2 groups are detected based on permutation analysis (red and black leaf nodes, Z threshold = 15, outlier threshold = 5). The black cluster contains the same samples as the 'bolted' cluster in Supplemental Figure 14B.**

**Based on this analysis, I think there's still a real risk that the plant population studied here has significant population substructure.**

**The authors suggest that the PCA of the transcriptome data (Supplemental Figure 6 in previous manuscript version) might show a pronounced separation between bolted and unbolted plants because the transcriptome shifts substantially at the onset of bolting. While this is a reasonable hypothesis, it is a bit at odds with the fact that the 'unbolted' cluster in the PCA also contains some bolted plants, and vice versa. Moreover, in the transcriptome PCA figure in the new manuscript version (Supplemental Figure 1), the left 'bolted' cluster exactly reflects the 'bolted' SNP-based phylogenetic clade on Supplemental Figure 14 (see relevant figures reproduced below as Figure R3), suggesting again that the transcriptome structure of the data is related to its SNP structure (regardless of whether or not the transcriptome changes substantially at bolting).**

**Regarding the links between SNP-subpopulations and bolting status/pseudotime visible in Figure 5B, the authors argue that this is due to the fact that they visualized variants that were found associated with pseudotime and hence bolting status. But the mere fact that such variants are found in sizeable proportions indicates that the genetic variation in the population influences the transcriptome and phenotype data. If these are just random SNPs in random individuals, this would not be an issue, but if the plants concerned form groups with distinct SNP profiles (which is at least indicated by the SNPRelate analysis), this is problematic (from the single-plant point of view at least).**

**Figure R3. Transcriptome PCA from Supplemental Figure 1 (left) and SNP-based phylogenetic tree from Supplemental Figure 14 (right).**

**The authors followed my suggestion to also predict the bolting status of plants from the transcriptome, and also made prediction models for leaf size, biomass and bolting status based on the variants. The variant models for leaf size and biomass indeed, as the authors highlight in their rebuttal, perform worse than the transcriptome models (unclear whether the associated R2 values are significantly different from 0). The authors further claim on line 298 that variant-based logistic regression model for bolting status had a comparable performance to gene-expression-based models, but I didn't find any statistics to support this. If variants can predict bolting status better than expected by chance, this may again indicate that population structure is an influential factor.**

**In summary, the new data generated by the authors still indicates that there may be substantial population structure effects in the data. This is in itself not necessarily a problem, nor does it invalidate e.g. the transcripts uncovered as predictive of certain phenotypes, but it is an issue when framed in the context of a 'single-plant -omics' study. For this reason, Cruz et al. (2020) decided to remove the (linear) SNP effects from their dataset, although the population structure was found to only impact the expression of 211 maize genes, 1 metabolite and 0/5 phenotypes at $q <= 0.05$ (linear mixed-effect models, Dataset EV2 of Cruz et al. 2020). As the impact of population structure on transcripts and phenotypes in the present study may be substantially higher than in Cruz et al. (2020), judging from the correlations between SNP structure, transcriptome structure and bolting status, I would certainly recommend the authors to similarly remove the systematic SNP effects from their data. Even though the substructure is much less pronounced**

than in Cruz et al. (2020), it does seem to affect the transcriptome and phenotype data much more than in than in Cruz et al. (2020), which calls for caution.

In fact, given the strong relationship between SNP structure, transcriptome, bolting status and pseudotime, I think it would be really worthwhile to look at which genes are affected by (or closely linked to) the SNPs that highly correlate with pseudotime in Figure 5, to check whether there would be any genes in there with known relationships to e.g. the floral transition. It remains rather puzzling to me however that the SNPs highly correlated with pseudotime and bolting status on Figure 5 do not seem to cluster in particular genomic regions. Could the authors elaborate on where the Ws-2 seeds they used came from?

Reviewer #3 (Comments for the Author):

Thank you for carefully addressing comments from the previous round.

**Reviewer #2 (Comments for the Author):**

**Dear Authors, Dear Editor,**

**I focused my review of the revised manuscript version on the potential subpopulation issue I flagged in my previous review, as I think resolving this potential issue is of primary importance and my other comments were generally addressed well (although I still believe the pseudotime inference algorithm used by the authors is suboptimal).**

We would like to thank Prof. Maere for his comments and the effort he has taken to analyse our data. In summary, we have made the following changes:

Small textual changes:

- We have included several additional changes to the text to provide more context on the genetic variation within the population.
- We have included information about the number of parent plants of the population.

Supplements that contextualise the main text, but do not change any claims:

- We have included the alternative phylogenetic tree provided by Prof. Maere
- We have included a linear-model-based analysis to provide more consistency with the methods of Cruz et al. (2020). These results are consistent with our original analysis.

However, we would like to respectfully disagree that having a complex population substructure influences the framing of the paper as a 'single-plant-omics' study. We are performing single-plant RNA-seq on what any standard plant science lab would call a near-isogenic population. The plants used in this study were bulked under typical conditions (see our response to comment 8 for details). Most *Arabidopsis* researchers would not find the approximately 1000 high-confidence variants which exist within their wild-type stocks, due to the low number of biological replicates used in typical RNA-seq experiments. Altogether, this highlights the potential impact of our dataset in uncovering the genetic sub-structure in lab strains.

Please note that line numbers refer to the *revised text*. We have also updated the GitHub repository (https://github.com/stressedplants/SinglePlantOmics) to contain the additional analysis.

1. The authors performed the analyses suggested in my previous review to check for distinct subpopulations in their sample of individual plants. In my opinion, not all the conclusions they draw from these analyses are equally well-supported however. While I agree that the SNP-based PCA results and the SNP-based phylogeny look less dramatic than in Cruz et al. (2020) (erroneously referred to by the authors as Bhosale et al. (2013)), there still seems to be a clear substructure in both of them.

We apologise for incorrectly citing the paper. Please note that this was cited incorrectly in the *reviewer response letter*, not in the manuscript text itself.

2. Regarding the PCA, the authors bring up that their PC1 only explains 10.8% of the variance, while the PC1 in Cruz et al. (2020) explained 48.7% of the variance. The authors take this as evidence that in their case the clustering seen along PC1 is weak. There's still a clear separation in Supplemental Figure 14A along the first PC however, and that this PC only explains 10.8% of the variance in the SNP dataset primarily indicates that their population structure is more complex than the one in Cruz et al. (2020), not that it is not important or influential.

Please note that the aspect of this discussion that Prof. Maere is concerned about was *in the reviewer response letter* (in arguing why the PCA helped address Prof. Maere's previous concerns), but not in the manuscript itself. However, we decided to add a brief statement in the main manuscript to highlight that the population structure is complex:

Line 287: 'We observed that a low amount of genetic variation was explained by PCA (Supplemental Figure 14), **highlighting the complex population structure**.'

3. That the population structure is more complex is also suggested by the structure of the phylogenetic tree in Supplemental Figure 14B (what is the unit of branch lengths here ?). There's a distinctive clade containing 18/23 bolted plants, which likely corresponds to the left 'bolted' cluster on the PCA plot (although this cannot be assessed with certainty because the PCA plot is unlabeled). On the other hand, the bootstrap support for this clade is really low, which is a bit at odds with the clear signal on the PCA plot. Could the authors elaborate more on how this tree has been made ? From the methods, I got that 'dist.gene' in the ape package was used as a distance, which should be the number of variant differences between samples. Was this dist.gene calculated on the 0-1-2 haplotype profiles, or directly on the VCF data somehow ? I was also a bit confused by the statement on line 288 that 'Bootstrapping analysis of a neighbour-joining tree trained on variants showed that subsets of variants predicted very different phylogenetic structures (Supplemental Figure 14B).', in the sense that bootstrapping is not the same as subsampling.

We thank Prof. Maere for highlighting the ambiguity in our methods and in our explanation of the results. Indeed, we did apply dist.gene to the 0-1-2 coded haplotype profiles (after we had removed all variants that were coded as heterozygous only). We have made this clearer by updating the methods and by including a citation to the paper suggested by the 'ape' package.

Line 619: The neighbour-joining algorithm was performed using the ape package (version 5.7-1) in R, using the commands 'nj', 'dist.gene' to count the number of differences in variants between samples after representation as integers, and 'boot.phylo' (with 'B = 1000') to repeat the analysis using bootstrapping (Efron et al. 1996; Paradis and Schliep 2019).

We believe that the low-support for most branches of the tree is consistent with our other results. Bootstrapping repeats the neighbour-joining method on variants selected *with replacement.* We have shown (in Figure 5) that only a small number of variants have an association with pseudotime (and therefore bolting status). Hence, it makes sense that the majority of resampled sets of variants do not include many pseudotime-associated variants.

We have stated our claim more clearly in terms of the agreement between the bootstrap trees and the original tree. This more closely follows the language from the original bootstrap publication (Efron et al. 1996).

Line 289: Analysis of a neighbour-joining tree, trained on variants, showed that bootstrap trees had low agreement with the original tree (Supplemental Figure 15A).

As explained further below, we have also included Prof. Maere's alternative method for constructing a phylogenetic tree for comparison.

4. The authors state that 'the bootstrap support for having the bolted plants in a separate clade is extremely weak (less than 0.1%)', but I'm not sure what this means. That not all bolted plants belong in a separate clade indeed readily seen already from the PCA, but this is not a strong argument for there being no relationship between population structure and bolting status. Even if not all bolting plants belong to a separate clade, if 18/23 of them do there is likely a relationship between population structure and bolting status. That this clade is not 'a distantly separated lineage' is of little consequence in this matter, as there might be only one or a few causative SNPs involved (possibly along with linked SNPs with no effect). What really matters is whether or not the bolted plants are found clustered in both the PCA and the phylogenetic tree. In order to further assess whether or not there may be significant genetic subpopulations of plants in the data, I performed a number of analyses on the VCF file the authors provide as Supplemental Table S14. A first analysis using TASSEL5 (as in Cruz et al. 2020) using 1-IBS (identity by state) as the distance measure produced a tree (Figure R1) that was even less informative than the one produced by the authors using ape (TASSEL does not provide bootstrap values), but it did show the same separation in two clades, with the top clade in Figure R1 corresponding to the 'bolted' clade in Supplemental Figure 14B. I also tried SNPhylo as an alternative SNP-based tree building program (maximum likelihood), but unfortunately got the message 'VCF file (<your_path>/36604_1_supp_1447730_sbsbx2.vcf) is too small to run this script!'. Figure R1. Archaeopteryx visualization of midpoint-rooted TASSEL5-inferred SNP tree based on filtered VCF file (SNPs that are heterozygous in all individuals removed, distance measure = 1-IBS). The top cluster contains the same samples as the 'bolted' cluster in Supplemental Figure 14B. As an alternative, I used the SNPRelate R package to perform an identity-by-state analysis on the VCF file in Supplemental Table S14 (without filtering) and to perform a clustering analysis on the resulting pairwise IBS distance matrix, which uses permutation Z-scores to automatically determine subgroups (Figure R2). This analysis did identify a significant split in two subgroups, with one of them identical to the 'bolted' clade in Supplemental Table S14. The R commands used for this analysis were the following (see also tutorial at the bottom of the following notebook : https://rpubs.com/adel922/560260) :

```
library(gdsfmt)
library(SNPRelate)

vcf.fn <- "<your_path>/36604_1_supp_1447730_sbsbx2.vcf"
snpgdsVCF2GDS(vcf.fn, "test.gds", method="biallelic.only")
# Summary
snpgdsSummary("test.gds")
genofile <- snpgdsOpen("test.gds")
```

```
set.seed(100)
ibs.hc <- snpgdsHCluster(snpgdsIBS(genofile, num.thread=2))
# Determine groups of individuals automatically
rv <- snpgdsCutTree(ibs.hc)
plot(rv$dendrogram, leaflab="perpendicular", main="SNPRelate tree")
```

Figure R2. SNPRelate hierarchical clustering tree. The Z score of 23.9 indicates that 2 groups are detected based on permutation analysis (red and black leaf nodes, Z threshold = 15, outlier threshold = 5). The black cluster contains the same samples as the 'bolted' cluster in Supplemental Figure 14B. Based on this analysis, I think there's still a real risk that the plant population studied here has significant population substructure.

Thank you for taking the time to provide this additional analysis. We believe that both bootstrapping and the permutation-test based analysis are valuable, due to the different methods used. The first analysis repeats neighbour-joining on bootstrapped sets of variants whereas the second analysis relabels the samples to perform the permutation test (using all the variants each time). Using both approaches shows that there is evidence for two subgroups, but also provides evidence that a small subset of variants may be linked to these subgroups.

We provided a more nuanced discussion about this in the results section: Line 291: An alternative method suggested that two subgroups may be present in the population based on the variant data (Supplemental Figure 15B).

Supplemental Figure 15 contains both methods for tree-building: using the ‘ape’ package and the ‘SNPRelate’ package. The legend now reads: **A:** Neighbour-joining tree on individual plants, followed by bootstrapping with 1000 repeats (Saitou and Nei 1987). Branch lengths represent the pairwise distance between samples – i.e. the number of variants where they differed. The labels on nodes represent how often that branch was observed when repeating the neighbour-joining method. **B:** An alternative approach to population structure analysis, using Identity-by-state analysis, from the SNPRelate package (Zheng et al. 2012). This differs to **A** since the method uses the ‘average’ metric during hierarchical clustering and performs a permutation test to find the number of distinct clusters. The Z-score on the top branch was 23.9, greater than the Z threshold of 15, indicating that two subgroups were present.

We added information about ‘SNPRelate’ in the methods. Line 623: A second analysis of population structure was performed using the SNPRelate package (version 1.32.2) in R, using ‘snpgdsIBS’ to calculate the Identity-by-state distance matrix, ‘snpgdsHCluster’ to perform hierarchical clustering, and ‘snpgdsCutTree’ to determine an appropriate number of subgroups using a permutation test (Zheng et al. 2012).

5. The authors suggest that the PCA of the transcriptome data (Supplemental Figure 6 in previous manuscript version) might show a pronounced separation between bolted and unbolted plants because the transcriptome shifts substantially at the onset of bolting. While this is a reasonable hypothesis, it is a bit at odds with the fact that the 'unbolted' cluster in the PCA also contains some bolted plants, and vice versa. Moreover, in the transcriptome PCA figure in the new manuscript version (Supplemental Figure 1), the left 'bolted' cluster exactly

reflects the 'bolted' SNP-based phylogenetic clade on Supplemental Figure 14 (see relevant figures reproduced below as Figure R3), suggesting again that the transcriptome structure of the data is related to its SNP structure (regardless of whether or not the transcriptome changes substantially at bolting).

Again, this comment refers to the discussion in the *reviewer response letter*, rather than the main manuscript text. However, we have now decided to highlight in the main manuscript Prof. Maere's observation that variants may help explain why some of the bolted / non-bolted plants were found in the wrong cluster, as this helps contextualise our results:

Line 314: Furthermore, the presence of these variants helps to explain why unbolted plants cluster with bolted plants and vice versa.

6. Regarding the links between SNP-subpopulations and bolting status/pseudotime visible in Figure 5B, the authors argue that this is due to the fact that they visualized variants that were found associated with pseudotime and hence bolting status. But the mere fact that such variants are found in sizeable proportions indicates that the genetic variation in the population influences the transcriptome and phenotype data. If these are just random SNPs in random individuals, this would not be an issue, but if the plants concerned form groups with distinct SNP profiles (which is at least indicated by the SNPRelate analysis), this is problematic (from the single-plant point of view at least).

Here Prof. Maere highlights an important point that will be of fundamental importance to Arabidopsis researchers: even near isogenic lab strains will have genetic variants that are associated with physiological characteristics. This point was always present in our original article (i.e. Figure 5 and associated results section). However, we agree that this point should be further highlighted in our abstract, introduction and discussion section, because it may result in a fundamental shift in perspective of the Arabidopsis community and may substantially increase the impact of our manuscript.

Please note that we respectfully disagree that this is problematic from a single-plant-omics point of view, because we are using extremely uniform plants: there are only around 1000 high confidence variants across all 68 individual plants. This work is as near to the ideal conditions of 'single plant omics' study that one can hope to achieve. The fact that there is still a (complex) population structure highlights the often neglected role of intra-population genetic variation in near isogenic lines, and we now propose that the Arabidopsis community ought to take this issue more seriously.

Abstract (line 26): Even in this near isogenic population, we find that some variants are associated with developmental traits.

Introduction (line 77): Additionally, we identified genetic variants that are associated with biological age.

Discussion (line 372): Our work also highlights that near isogenic lab strains may still contain genetic variants that are associated with traits of interest, suggesting that experiments need to be designed to robustly handle heterogeneity between individual plants.

The complex population structure of near isogenic lines of laboratory strains warrants further consideration by the research community.

7. The authors followed my suggestion to also predict the bolting status of plants from the transcriptome, and also made prediction models for leaf size, biomass and bolting status based on the variants. The variant models for leaf size and biomass indeed, as the authors highlight in their rebuttal, perform worse than the transcriptome models (unclear whether the associated R2 values are significantly different from 0). The authors further claim on line 298 that variant-based logistic regression model for bolting status had a comparable performance to gene-expression-based models, but I didn't find any statistics to support this. If variants can predict bolting status better than expected by chance, this may again indicate that population structure is an influential factor.

We thank Prof. Maere for pointing out that we were vague in our discussion of the logistic regression results for variants. When qualitatively looking at the results of the logistic regression, they appeared ‘comparable’, but on closer examination, the logistic regression model for bolting status using the transcriptome did perform *better* than using the variants (which strengthens our argument). Our text now lists the accuracies of the models:

Line 307: The variant-based logistic regression model for bolting status performed slightly worse (76.5% accuracy) than the logistic regression models that used the full transcriptome (80.8% accuracy) or the transcriptome of regulatory genes (85.2% accuracy) (Figure 2, Supplemental Figure 4 and 15).

*This replaces: However, the variant-based logistic regression model for bolting status had a comparable performance to gene-expression-based models (Figure 2, Supplemental Figure 4 and 15).*

8. In summary, the new data generated by the authors still indicates that there may be substantial population structure effects in the data. This is in itself not necessarily a problem, nor does it invalidate e.g. the transcripts uncovered as predictive of certain phenotypes, but it is an issue when framed in the context of a 'single-plant -omics' study. For this reason, Cruz et al. (2020) decided to remove the (linear) SNP effects from their dataset, although the population structure was found to only impact the expression of 211 maize genes, 1 metabolite and 0/5 phenotypes at q <= 0.05 (linear mixed-effect models, Dataset EV2 of Cruz et al. 2020). As the impact of population structure on transcripts and phenotypes in the present study may be substantially higher than in Cruz et al. (2020), judging from the correlations between SNP structure, transcriptome structure and bolting status, I would certainly recommend the authors to similarly remove the systematic SNP effects from their data. Even though the substructure is much less pronounced than in Cruz et al. (2020), it does seem to affect the transcriptome and phenotype data much more than in than in Cruz et al. (2020), which calls for caution.

In Cruz et al. (2020), a mixed effect model was used to correct for SNP structure. We have included a similar linear model-based analysis in the Supplementary materials. This is useful for consistency with Cruz et al. (2020) and gave us an opportunity to mention the association between variants and transcriptomes.

We have highlighted these results in the main text. Line 292: By fitting linear models linking these two subgroups of samples with gene expression, we found that 13545 genes had an association with the variant-based subgroups of samples (adjusted p-value less than 0.05; Supplemental Figure 14; Table S16). This is consistent with the facts that the subgroups largely separate out bolted and non-bolted plants and that 55% of the transcriptome is differentially expressed between the bolted and non-bolted plants.

However, we do not feel like it is appropriate to repeat the analysis on the 'adjusted' data, because it makes the interpretation of the results more convoluted. For instance, we can say in our current manuscript that the expression of certain genes was consistently identified as predictors of biomass and/or leaf size. *This statement is making no claims about the underlying causes of this association between gene expression and physiology (i.e. whether they are genetic or random noise) and so does not need a correction.*

If we correct for gene expression based on population structure, we would inadvertently also be removing biologically-meaningful differences in gene expression associated with bolting status. We are much happier reporting our observed associations, without adding unnecessary complications in interpretation.

This is supported by Prof. Maere's statement that: 'this is in itself not necessarily a problem, nor does it invalidate e.g. the transcripts uncovered as predictive of certain phenotypes'. We are not trying to do exactly what was done in Cruz et al. (2020) – both data analysis philosophies are justifiable and have different strengths and weaknesses, reflecting the different underlying goals of the projects.

We have also made changes associated with the linear models in the Methods and Supplemental Information.

Table S16 contains the relevant statistics for the linear models. Table S16 – Statistics for linear models based on variant subgroups. This Excel sheet contains a summary of the linear model trained per gene. The response variable was the log-transformed gene expression, and the covariates included a constant term (to account for mean gene expression) and the variant subgroups (as defined in Supplemental Figure 14). This Excel sheet contains values produced by 'summary.lm' in R, namely: the estimate and standard error for the subgroup coefficient, and the t-value, two-sided p-value and adjusted p-value (using the Benjamini and Hochberg method to control the false discovery rate) for the associated t-test (Benjamini and Hochberg 1995).

Additional information in Supplemental Figure 14, which now only contains the PCA, explains the two subgroups: The vertical line follows PC1 = -2.25. Samples to the left of the line were assigned to subgroup 1 for the linear model analysis and contained mostly bolting samples (see Methods and 'A small number of variants are associated with pseudotime'), and the other samples were assigned to subgroup 2 and contained mostly non-bolting samples.

The 'variant calling' section of the methods includes information about linear models. Line 629: To assess the relationship between the suggested population subgroups and gene expression, we ran linear models to predict log-transformed gene expression (i.e. log_2(x+1) where x is measured as TPM) from the subgroups of samples, based on variants (Table

S16). This closely follows the linear mixed effects models used to assess the effect of population structure in (Cruz et al. 2020), except that we did not need to account for effects such as harvest date, sequencing batch, or spatial autocorrelation. We separated out the two subgroups, based on the two large clusters from the variant-based PCA (Supplemental Figure 14). Note, these subgroups were consistent with the two subgroups predicted by the SNPRelate analysis (Supplemental Figure 15B). We adjusted the p-values for the associated t-tests, using the Benjamini and Hochberg method to control the false discovery rate (Benjamini and Hochberg 1995).

9. In fact, given the strong relationship between SNP structure, transcriptome, bolting status and pseudotime, I think it would be really worthwhile to look at which genes are affected by (or closely linked to) the SNPs that highly correlate with pseudotime in Figure 5, to check whether there would be any genes in there with known relationships to e.g. the floral transition. It remains rather puzzling to me however that the SNPs highly correlated with pseudotime and bolting status on Figure 5 do not seem to cluster in particular genomic regions. Could the authors elaborate on where the Ws-2 seeds they used came from?

The Ws-2 seed stock that we have has been propagated for many generations in our lab and has been used in prior publications by the Davis group, including the one already cited (Anwer et al, 2020). Our bulking protocol is standard for the discipline: we grow four to five Ws-2 seeds per pot in a greenhouse, and used 3 pots, so the seeds will come from a mix of 15 parent plants. We have included a sentence referring to this in the methods:

Methods (line 404): Seeds from the Arabidopsis ecotype Wassilewskija (Ws-2) (Anwer et al., 2020) **came from a collection of approximately 15 parents. These were surface-sterilised and plated onto…**

Please note that we have initiated a new project in our lab related to: 'which genes are affected by (or closely linked to) the SNPs that highly correlate with pseudotime in Figure 5, to check whether there would be any genes in there with known relationships to e.g. the floral transition.' but that this constitutes a separate story that will take substantial experimental work to fully validate. We have also noticed the same observation as Prof. Maere about the chromosomal distribution of associated variants ('It remains rather puzzling to me however that the SNPs highly correlated with pseudotime and bolting status on Figure 5 do not seem to cluster in particular genomic regions.'), which suggests that this is not a result of linkage disequilibrium but more likely some kind of epistatic interaction. This is precisely why this is a challenging, long-term endeavour, beyond the scope of this project.

Reviewer #3 (Comments for the Author):
Thank you for carefully addressing comments from the previous round.
We thank the reviewer for their support.

2nd Revision - Editorial Decision 20-Jun-2024

Dear Dr. Daphne Ezer:

We have performed an editorial evaluation of your manuscript entitled "Single-plant-omics reveals the cascade of transcriptional changes during the vegetative-to-reproductive transition." The board of reviewing editors would like to accept your manuscript for publication in The Plant Cell. This acceptance is contingent on revision based on consultation with members of our editorial board team that have relevant expertise in the subject area of the manuscript. In particular, please consider the following:

We appreciate your extensive attention to all of the manuscript improvements requested by the reviewers, and understand the remaining disagreement about the use of the phrase "single-plant-omics". We agree that single plant RNA-seq doesn't necessarily have to be done on clonally identical plants in order to be interesting and worthwhile for readers.

We understand that you have included in this new analysis the fact that the seeds used came from a bulk of at least 15 plants. It is certainly possible (and the SNP data suggest but don't conclusively prove) that one or more of those 15 progenitors carried a point mutation that resulted in earlier flowering and the progeny of that/those progenitor(s) tended to flower slightly earlier than the rest of the plants in that batch. Other mutations that were fixed in the lineage will look like they are linked to flowering time, and other mutations that effect expression of one or more genes which are fixed in that lineage will make it look like the expression of that gene is linked to flowering time.

As both you and reviewer Maere have pointed out, this doesn't invalidate the interesting nature of the results. However, it does seem important to mention in the section from 288-302 that the seeds used in this study are the progeny of 15 different plants, as this seems likely to explain why one sees two subpopulations: the seeds used are descended from a small number of multiple parents, and it seems likely those parents constituted two or more lineages with different fixed nucleotide changes.

We agree that it isn't necessary to redo the whole analysis of gene expression using the adjusted data, but it is important to explicitly acknowledge the potential for false positive gene expression-trait associations that result from multiple lineages with different fixed SNPs present within the experiment.

We would like to confirm that these two changes have been made in the manuscript before moving forward with publication.

We also appreciate the suggestion to ask Dr. Maere if he would like to be included in the acknowledgments, and we will ask him about this and let you know the outcome.

Please highlight all changes and include a detailed annotation of changes of the text, with line numbers, and noting your responses to the comments.

To submit your revised manuscript, click:

Link Not Available

If you have any questions about the revision submission procedures, please contact the Editorial Office Staff (tpc-submissions@aspb.org). If you cannot return the revised manuscript within 30 days, please let us know. Otherwise, we will assume that you have elected not to revise the manuscript and withdraw it.

Thank you very much for the privilege of handling this work, and we look forward to receiving the next version.

Sincerely,

The Plant Cell Editorial Board

**Please also note the following:**

-The Plant Cell now requires authors to complete and submit an author revisions checklist upon submission of a revised manuscript. The aim of the checklist is to aid authors in preparing a high-quality manuscript, facilitate the review and assessment of revised manuscripts, and help to ensure that journal standards are maintained across the board. If your manuscript is accepted, the completed checklist will be published as supplemental material attached to the article online. Please download a copy of the checklist (pdf fillable form) at this link, for submission with your revised manuscript:

https://tpc.msubmit.net/html/Author_Revisions_Checklist.pdf.

-Supplemental materials should be restricted to large datasets and tables, presentation of replicates, and validation of reagents, methods, or genotypes. Any data that are used to support the major claims must be in the main manuscript. Supplemental figure legends must indicate what figure in the main manuscript is supported by the supplemental data presented. Please justify how each of the supplemental figures meet the criteria.

-Sampling methods and the nature of "biological replicates" should be described precisely (i.e. different plants, parts of plants, pooled tissue, independent pools of tissue, sampled at different times, etc.), along with a clear description of and rationale for any statistical analyses conducted. The reader should know exactly what was sampled; what forms the basis of the calculation of any means and statistical parameters reported. This is also necessary to ensure that proper statistical analysis was conducted.

Dear Board of Reviewing Editors:

Thank you for accepting our manuscript, subject to these small changes. Both textual changes have been made. We are very pleased with the entire reviewing process and thought that all the communications from the editors have been consistently fair and thoughtful. We are particularly grateful for the flexibility provided to us when the lead author took a leave of absence due to personal circumstances. We are looking forward to sending additional manuscripts to *The Plant Cell* in the future.

Best wishes,
Dr. Daphne Ezer

****************************************

Dear Dr. Daphne Ezer:

We have performed an editorial evaluation of your manuscript entitled "Single-plant-omics reveals the cascade of transcriptional changes during the vegetative-to-reproductive transition." The board of reviewing editors would like to accept your manuscript for publication in The Plant Cell. This acceptance is contingent on revision based on consultation with members of our editorial board team that have relevant expertise in the subject area of the manuscript. In particular, please consider the following:

We appreciate your extensive attention to all of the manuscript improvements requested by the reviewers, and understand the remaining disagreement about the use of the phrase "single-plant-omics". We agree that single plant RNA-seq doesn't necessarily have to be done on clonally identical plants in order to be interesting and worthwhile for readers.

1. We understand that you have included in this new analysis the fact that the seeds used came from a bulk of at least 15 plants. It is certainly possible (and the SNP data suggest but don't conclusively prove) that one or more of those 15 progenitors carried a point mutation that resulted in earlier flowering and the progeny of that/those progenitor(s) tended to flower slightly earlier than the rest of the plants in that batch. Other mutations that were fixed in the lineage will look like they are linked to flowering time, and other mutations that effect expression of one or more genes which are fixed in that lineage will make it look like the expression of that gene is linked to flowering time.

   As both you and reviewer Maere have pointed out, this doesn't invalidate the interesting nature of the results. However, it does seem important to mention in the section from 288-302 that the seeds used in this study are the progeny of 15 different plants, as this seems likely to explain why one sees two subpopulations: the seeds used are descended from a small number of multiple parents, and it seems likely those parents constituted two or more lineages with different fixed nucleotide changes.

**We agree with this sentiment and agree that it helps contextualise the work. We have now made the required changes in the lines suggested, using the wording suggested:**

**Lines 292-294: The seeds used in this experiment are the progeny of 15 different plants and those parents likely constitute two or more lineages with fixed nucleotide changes.**

2. We agree that it isn't necessary to redo the whole analysis of gene expression using the adjusted data, but it is important to explicitly acknowledge the potential for false positive gene expression-trait associations that result from multiple lineages with different fixed SNPs present within the experiment.

   **We also agree that this needs to be stated more explicitly in our text. We used the phrase 'variants' to be consistent with the rest of this section.**
   **On lines 209-302 we now state: 'This analysis also suggests that there is a potential for false positive gene expression-trait associations that result from multiple lineages with different fixed variants present within the experiment.'**

We would like to confirm that these two changes have been made in the manuscript before moving forward with publication.

We also appreciate the suggestion to ask Dr. Maere if he would like to be included in the acknowledgments, and we will ask him about this and let you know the outcome.

Please highlight all changes and include a detailed annotation of changes of the text, with line numbers, and noting your responses to the comments.

To submit your revised manuscript, click:

https://tpc.msubmit.net/cgi-bin/main.plex?el=A5Is4Nbw6C7BDXj5I5A9ftdoBccDdAQvI14UD9aKiVIgQZ

If you have any questions about the revision submission procedures, please contact the Editorial Office Staff (tpc-submissions@aspb.org). If you cannot return the revised manuscript within 30 days, please let us know. Otherwise, we will assume that you have elected not to revise the manuscript and withdraw it.

Thank you very much for the privilege of handling this work, and we look forward to receiving the next version.

Sincerely,

The Plant Cell Editorial Board

3rd Accepted Version - Editorial Decision 01-Jul-2024

Dear Dr. Daphne Ezer:

We are pleased to inform you that your paper entitled "Single-plant-omics reveals the cascade of transcriptional changes during the vegetative-to-reproductive transition" has been accepted for publication in The Plant Cell, pending a final editorial review by a science editor. At this stage, your manuscript will be evaluated by a science editor with respect to its presentation of scientific content, compliance with journal policies, and presentation for a broad readership. The Plant Cell has contracted with Plant Editors (planteditors.com) to provide this service to our authors, and you will soon receive additional information on this process.

We communicated with Steve Maere and he appreciated the idea of being acknowledged for use of his code in the analysis, it would be great to go ahead and include this acknowledgement in the final manuscript.

**Please note that each author needs to link their ORCID identifier to their account in the system before your manuscript can be published. If any authors do not have an ORCID linked to their account, they will receive a message with a link to complete this task. Please ensure that ALL of your coauthors have completed this task as soon as possible.**

ASPB offers an OPEN option that allows authors to have their online articles available for free to all users immediately upon publication. For more information about the ASPB OPEN option, refer to the Final Submission Checklist Form.

The Plant Cell and The Arabidopsis Information Resource (TAIR) are collaborating to collect functional annotation data about Arabidopsis genes from authors. This includes information about the gene's molecular function (e.g., kinase activity, ATP synthetase activity), the biological process/es it is involved in (e.g., endosperm development, threonine biosynthesis), its subcellular location (e.g., nucleus, ER), anatomical or developmental expression pattern (e.g., leaf, ovule, flower stage 10, seedling stage), or its partner in a protein-protein interaction (e.g., AT1G01010 interacts with AT1G01020).

If your paper contains results falling into one or more of these categories for Arabidopsis genes, we request that you now submit these data for inclusion in TAIR by filling in the form provided at the following URL: https://www.arabidopsis.org/doc/submit/functional_annotation/123. If you need further clarification on what types of data can be submitted please contact curator@arabidopsis.org.

Finally, we encourage your submission of artwork for the journal cover. Monthly issues will have an online cover image and selected covers will be used for posters, other promotional items, and "wallpaper" for mobile devices. For more information, refer to Cover Submission in the Instructions for Authors https://tpc.msubmit.net/cgi-bin/main.plex?form_type=display_auth_instructions.

We look forward to seeing your paper published.

Sincerely,

The Plant Cell Editorial Board

=====
IMPORTANT REMINDER: PEER REVIEW REPORTS
=====

If you opted to publish a peer review report along with your article during the original submission process, it will be prepared by the editorial staff and publicly posted with your manuscript, inside the zip file that contains any other supplemental material. As a reminder, the peer review report is a public record of all comments from editors and reviewers, as well as your prior responses, as you received them in the decision letters for each draft of your manuscript. If you agreed to publish this report and have changed your mind, or are not sure if you selected this option, please contact the editorial office as soon as possible before signing the license agreement from our publisher.

----FOR ASPB OFFICE USE ONLY (DO NOT EDIT)----
MSID: 36604
Corresponding Author: Dr. Ezer
